# Strong Stabilization of Co Nanoparticles by CeO_2‐x_ Clusters in Inverse CeO_x_/Co Catalysts for Enhanced CO_2_ Methanation

**DOI:** 10.1002/adma.202510593

**Published:** 2025-09-25

**Authors:** Yu Gao, Valery Muravev, Yonghui Fan, Hao Zhang, Jorden Wagemakers, Alexander Parastaev, Nikolay Kosinov, Emiel J. M. Hensen

**Affiliations:** ^1^ Laboratory of Inorganic Materials and Catalysis Department of Chemical Engineering and Chemistry Eindhoven University of Technology Eindhoven 5600 MB The Netherlands

**Keywords:** ceria, CO_2_ methanation, cobalt, inverse catalysts, metal‐support interactions

## Abstract

Inverse catalysts, where metal oxide species are dispersed over metallic nanoparticles, represent a promising class of materials for accelerating various chemical reactions. However, stabilizing metal nanoparticles with a small amount of oxide clusters remains a significant challenge, as the metallic phase tends to sinter under reaction conditions due to insufficient immobilization. In this study, flame spray pyrolysis is employed to synthesize uniformly sized inverse CeO_x_/Co catalysts for CO_2_ methanation (Sabatier reaction). It is found that small, highly reducible CeO_2‐x_ clusters effectively stabilize metallic cobalt nanoparticles, thereby preventing sintering even during hydrogen reduction at 500 °C and during CO_2_ hydrogenation. Detailed *operando* characterization demonstrates that this stabilization leads to a high density of metallic Co sites interfaced with CeO_2‐x_ clusters, which facilitates CO_2_ activation into carbonyl (CO^*^) intermediates, resulting in significantly enhanced CH_4_ formation rates. Notably, an inverse CeO_x_/Co catalyst containing 20 mol% Ce exhibits a methanation rate an order of magnitude higher than that of a CeO_2_‐free Co catalyst. These findings highlight the dual role of CeO_2‐x_ clusters in both stabilizing Co nanoparticles and enhancing catalytic performance, offering a robust strategy for improving CO_2_ hydrogenation performance.

## Graphical abstract

Inverse CeO_x_/Co catalysts are promising new materials for CO_2_ methanation. Operando spectroscopy reveals that small, reducible CeO_2‐x_ clusters effectively stabilize Co nanoparticles from sintering during H_2_ reduction and promote CO_2_ activation at the Co‐CeO_2_ interface. This leads to abundant metallic Co surface sites and a high coverage of surface carbonyl intermediates, thereby facilitating CH_4_ formation.

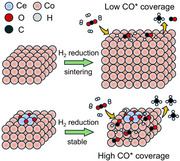

## 1 Introduction

Transition metal nanoparticles will play an essential role in the energy transition as they form the active phase in catalysts for CO_2_ hydrogenation, biomass valorization, and plastic recycling.^[^ ^1^, ^2^, ^3^ ^]^ One of the main drawbacks of metal nanoparticle catalysts is sintering under reaction conditions.^[^ ^4^, ^5^ ^]^ Dispersing metal nanoparticles on high‐surface‐area metal oxide support materials can significantly hinder sintering. Reducing the active metal loading is a common way to suppress sintering, but it usually results in lower mass‐specific activity (i.e., activity per unit of catalyst weight).^[^ ^6^ ^]^ Inverse catalysts, where the metal surface is modified with a small amount of metal oxides, have attracted considerable attention in recent years.^[^ ^7^, ^8^, ^9^ ^]^ Small metal oxide clusters are often more defective than metal oxide nanoparticles and interact more strongly with metals, explaining the distinct reactivity at interfacial metal‐metal oxide sites.^[^ ^10^, ^11^ ^]^ Previous studies by Rodríguez et al. have highlighted the potential of inverse catalysts for various applications, such as water‐gas shift (WGS) and CO_2_ hydrogenation reactions.^[^ ^8^, ^12^, ^13^ ^]^ These studies also highlighted the potential for tuning reactivity through the use of various metal oxides. The Sabatier reaction, which transforms CO_2_ into CH_4_, has recently attracted significant interest as a promising strategy for reducing CO_2_ emissions and enabling renewable energy storage, particularly when employing transition metal‐based inverse catalysts.^[^ ^14^, ^15^, ^16^, ^17^ ^]^ Despite the growing body of research, a comprehensive mechanistic understanding of how inverse catalysts operate during the Sabatier reaction remains elusive.

Previously, we demonstrated that enhanced metal‐support interactions (MSI) in a CeZrO_x_‐supported Co catalyst effectively stabilized Co nanoparticles, leading to high CO_2_ methanation rates.^[^ ^18^ ^]^ Based on this insight, we hypothesized that a small proportion of CeO_2_ clusters could similarly stabilize Co nanoparticles. In this study, we prepared inverse CeO_x_/Co catalysts containing 1–20 mol% Ce (relative to the total Ce + Co molar content) through a single‐step flame spray pyrolysis (FSP) process. Variations in the MSI were investigated by temperature‐programmed H_2_ reduction measurements of the inverse catalysts. Electron microscopy and in situ X‐ray diffraction studies revealed that small, reducible CeO_2‐x_ clusters formed on the surface of Co nanoparticles during reduction in H_2_. These clusters effectively stabilized Co nanoparticles against sintering, even at elevated temperatures up to 500 °C in H_2_ and during CO_2_ hydrogenation. Mechanistic studies indicated that the Co‐CeO_2_ interactions enhanced CO_2_ activation, leading to increased formation of surface carbonyls (CO^*^) on the metallic Co surface, which are the key intermediates for CH_4_ formation. Consequently, the inverse CeO_x_/Co catalysts demonstrated higher Co weight‐normalized CH_4_ formation rates than a CeO_2_‐free Co catalyst. Moreover, their performance surpassed that of state‐of‐the‐art Co catalysts reported in the literature for CO_2_ methanation.

## 2 Results and Discussion

### 2.1 Structure of Inverse CeO_x_/Co Catalysts

**Figure 1:**
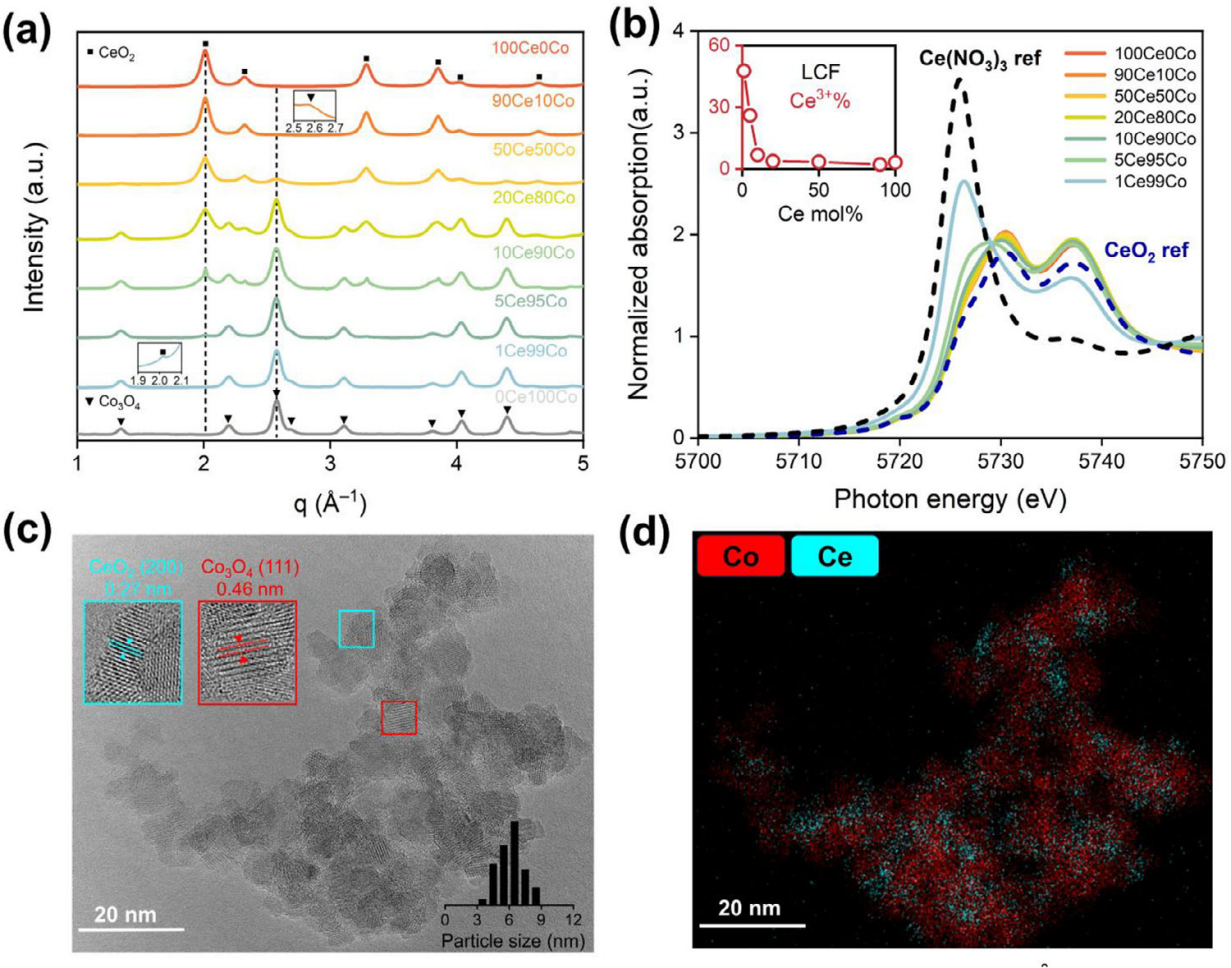
a) Synchrotron XRD patterns of as‐prepared samples recorded at λ = 0.1653 Å; b) Ce *L*_3_‐edge XANES spectra of as‐prepared samples. Inset: linear combination fitting (LCF) results of the XANES spectra using bulk CeO_2_ and Ce(NO_3_)_3_ as references; c) HRTEM image of as‐prepared 10Ce90Co. Inset: the particle size distribution, CeO_2_(200) and Co_3_O_4_(111) phases and d) HAADF‐STEM‐EDX map of as‐prepared 10Ce90Co.

Catalysts were prepared by FSP in one step from cobalt acetylacetonate and cerium acetate precursors (xCeyCo, in which x and y represent the molar fractions (%) of Ce and Co, with x + y = 100, x = 0, 1, 5, 10, 20, 50, 90, and 100). Samples with Ce fractions between 0 and 20 mol% are considered inverse catalysts. Synchrotron XRD patterns of the as‐prepared samples reveal diffraction lines of Co_3_O_4_ and CeO_2_ (**Figure** **1**a; Figure S1, Supporting Information). Inverse samples contain small crystalline CeO_2_ clusters, as evidenced by the broadening of the CeO_2_ diffraction lines. The absence of shifts in the position of the diffraction peaks of CeO_2_ and Co_3_O_4_ when varying the Co and Ce loading suggests no isomorphous substitution between Co and Ce in the as‐prepared samples. X‐ray absorption near‐edge structure (XANES) was used to determine the oxidation states of Co and Ce in these samples. Figure 1b demonstrates a gradual evolution in the shape of Ce *L*_3_‐edge XANES, shifting from spectra resembling the Ce^4+^ reference (CeO_2_) to the Ce^3+^ reference (Ce(NO_3_)_3_) with decreasing Ce content. Linear combination fitting (LCF) confirms this trend (Table S2, Supporting Information). The 1Ce99Co and 5Ce95Co samples with a low Ce content contain ≈48% and ≈26% Ce^3+^, respectively. In contrast, samples with a high Ce content, typical for standard Co/CeO_2_ catalysts, 90Ce10Co and 100Ce0Co contain less than 5% Ce^3+^. These findings are consistent with previous studies, which show that small CeO_2_ clusters are more defective than their bulk counterparts.^[^ ^19^, ^20^ ^]^ In the following, such defective clusters will be denoted as CeO_2‐x_. Co *K*‐edge XANES spectra and corresponding *k* ^3^‐weighted Fourier transforms of extended X‐ray absorption fine structure (FT‐EXAFS) demonstrate that Co_3_O_4_ is the dominant Co‐oxide phase in all as‐prepared samples, except for 90Ce10Co, in which a CoO feature is observed as well (Figure S2, Supporting Information). Given that XAS provides mainly bulk information, X‐ray photoelectron spectroscopy (XPS) was employed to characterize the surface of the samples. The fitting results of Ce 3d core‐line XPS spectra exhibit a similar trend with a higher fraction of Ce^3+^ in the inverse catalysts (Figures S3 and S4, Table S3, Supporting Information). In line with XAS data, Co 2p core‐line XPS results indicate a higher concentration of Co^2+^ in the 90Ce10Co and 50Ce50Co samples (Figures S3 and S5, Supporting Information), which can be attributed to CoO stabilization by CeO_2_ particles.^[^ ^21^, ^22^ ^]^ High‐resolution transmission electron microscopy (HRTEM) images and high‐angle annular dark‐field scanning transmission electron microscopy‐energy dispersive X‐ray spectroscopy (HAADF‐STEM‐EDX) results for 10Ce90Co and 20Ce80Co show the presence of Co_3_O_4_ nanoparticles with an average size of 6–7 nm, along with CeO_2‐x_ clusters (Figure 1c,d; Figures S6 and S7, Supporting Information). These findings are consistent with the particle sizes determined from the XRD data (Figure S1, Supporting Information).

### 2.2 Strong Co‐CeO_2_ Interactions in Inverse Catalysts

**Figure 2:**
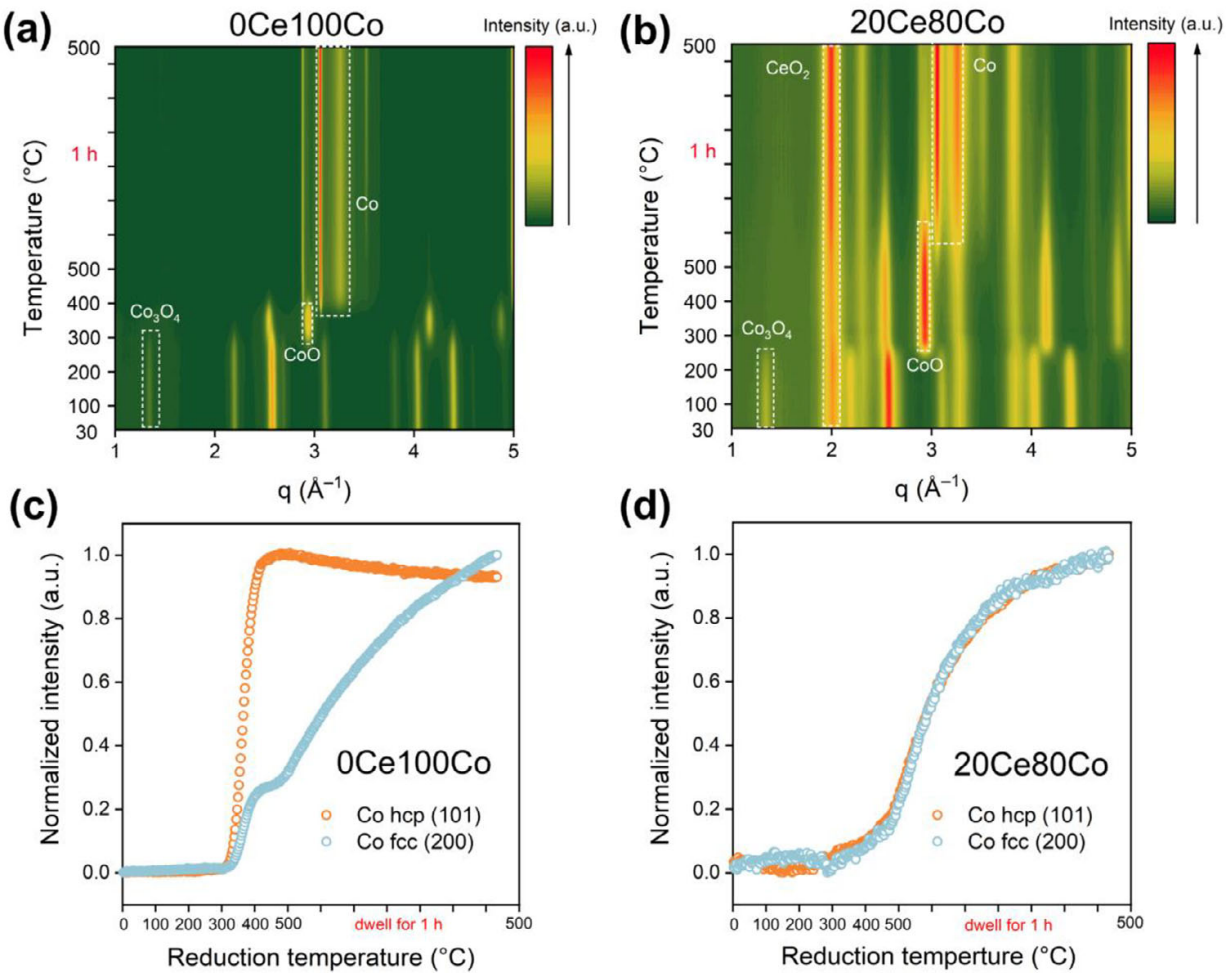
In situ XRD patterns of (a) 0Ce100Co and b) 20Ce80Co during H_2_ reduction. The temperature increased from room temperature to 500 °C followed by a dwell for 1 h; evolution of Co hcp and fcc phases in (c) 0Ce100Co and d) 20Ce80Co during the in situ XRD experiment (normalized to the highest intensity of a particular phase).

H_2_‐temperature programmed reduction (H_2_‐TPR) was used to study the reducibility of the FSP‐derived samples. As shown in Figure S8 (Supporting Information), the reduction of Co^2+^ to Co^0^ occurs at higher temperatures in 5Ce95Co, 10Ce90Co, and 20Ce80Co than in the other samples, indicative of stronger interactions between Co and CeO_2_ in the inverse catalysts (Note S1, Supporting Information). In situ synchrotron‐XRD was employed to study these interactions better, viz., by studying the evolution of the Co species during reduction. 0Ce100Co and 20Ce80Co were reduced in 10 vol% H_2_ in He (50 mL min^−1^) from room temperature to 500 °C, followed by a dwell at 500 °C of 1 h. The reduction of Co_3_O_4_ to CoO was completed before 300 °C in both samples, although the CoO species in 20Ce80Co were converted to metallic Co much more slowly than those in 0Ce100Co (**Figure** **2**a,b). A small amount of CoO species persisted in 20Ce80Co, even after reduction at 500 °C for 1 h (Figure S9, Supporting Information). The results indicate that CeO_2‐x_ clusters stabilized the CoO phase, preventing its rapid reduction in H_2_. Further analysis revealed that CeO_2‐x_ clusters also altered the formation kinetics of the hcp and fcc Co metal allotropes, suggesting a crucial role of CeO_2‐x_ in stabilizing and modulating the catalyst structure. Typically, the hcp‐Co phase is obtained by reduction of Co‐oxides in H_2_ at lower reduction temperatures, while it transforms into fcc‐Co at temperatures above 400 °C.^[^ ^23^ ^]^ Such behavior was observed by XRD for the 0Ce100Co sample, where the intensity of the hcp‐Co diffraction features reached their maximum at ≈400 °C, followed by a gradual decline with increasing temperature concomitant with an increasing intensity of fcc‐Co features (Figure 2c; Figure S10, Supporting Information). The role of CeO_2‐x_ clusters is evident from the slower and nearly equal formation of hcp‐Co and fcc‐Co in the 20Ce80Co sample (Figure 2d). The evolution of the Co and CeO_2_ crystallite sizes in 20Ce80Co and 0Ce100Co was analyzed following the fcc‐Co(101) and CeO_2_(111) diffraction peaks during H_2_ reduction (Figure S11, Supporting Information). The metallic Co particles in 20Ce80Co exhibited a strong resistance to sintering, with the fcc‐Co crystallite size derived from the fcc‐Co(101) remaining stable at ≈8.5 nm after its formation at ≈450 °C, regardless of subsequent reduction at 500 °C. The crystallite size derived from the CeO_2_(111) diffraction line in 20Ce80Co showed a minor increase from ≈3.0 to 4.8 nm over the entire reduction process. In contrast, fcc‐Co formed already at ≈350 °C in 0Ce100Co, and its crystallite size increased from ≈13.0 to 25.5 nm as the reduction temperature was increased to 500 °C.

**Figure 3:**
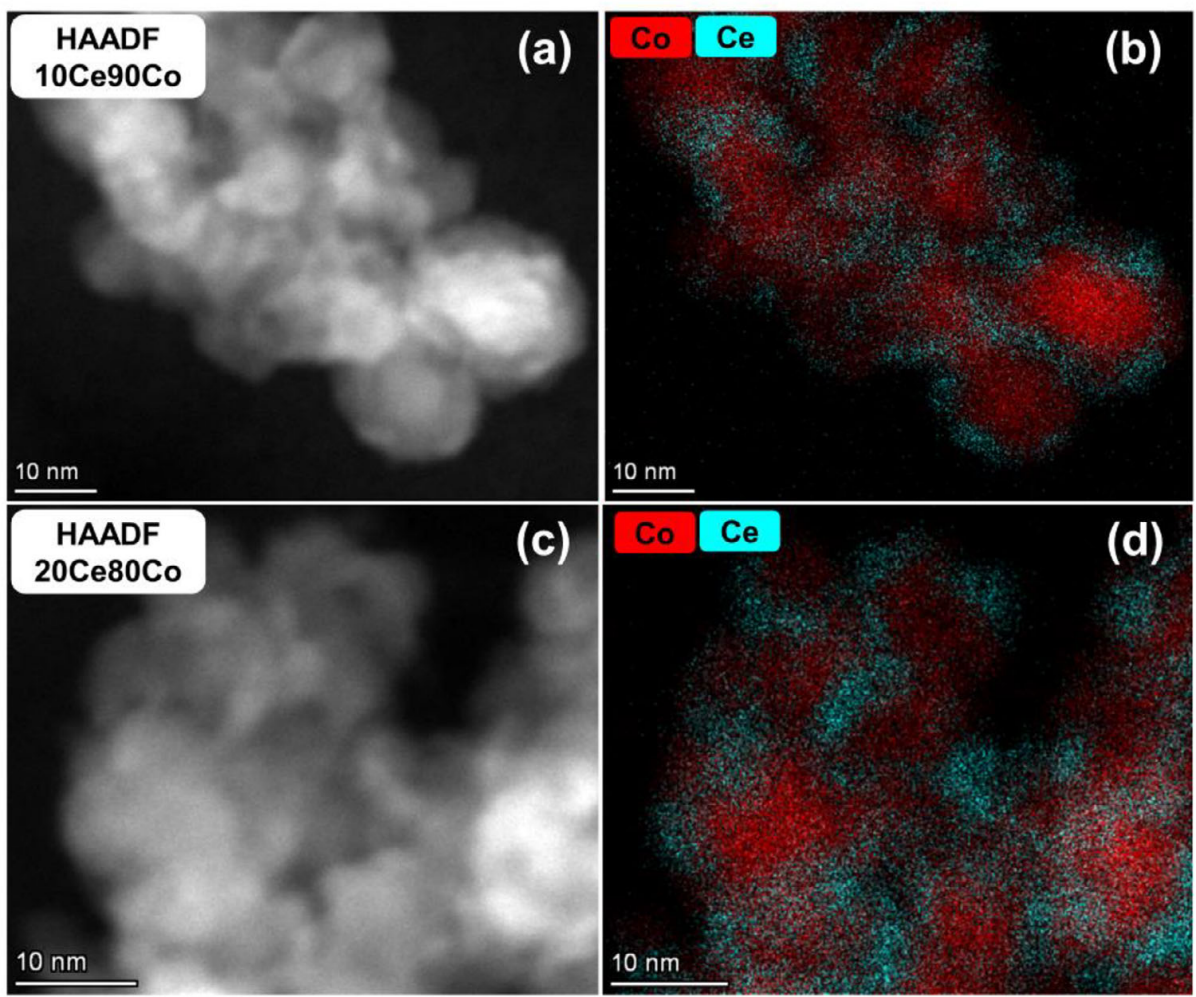
HAADF‐STEM images and corresponding STEM‐EDX mapping analysis of (a, b) 10Ce90Co and c, d) 20Ce80Co after reduction in 10% vol H_2_ in He (50 mL min^−1^) at 300 °C for 4 h. Samples were exposed to air after reduction.

TEM images of 0Ce100Co after reduction at 300 °C in H_2_ for 4 h indicate strong sintering, with particles becoming larger than 50 nm (Figure S12, Supporting Information). Notably, the 1Ce99Co sample treated similarly contains much smaller particles of ≈30 nm. When the Ce content is increased, the Co particle sizes decrease to ≈16 and ≈13 nm in 10Ce90Co and 20Ce80Co (Figure S13, Supporting Information), respectively. These results highlight the critical role of CeO_2‐x_ clusters in stabilizing Co nanoparticles. HAADF‐STEM images and corresponding STEM‐EDX mapping analysis of 10Ce90Co and 20Ce80Co reveal abundant CeO_2‐x_ patches on the Co surface after H_2_ reduction (**Figure** **3**), stabilizing Co nanoparticles and providing abundant interfacial sites.

### 2.3 CO_2_ Hydrogenation

**Figure 4:**
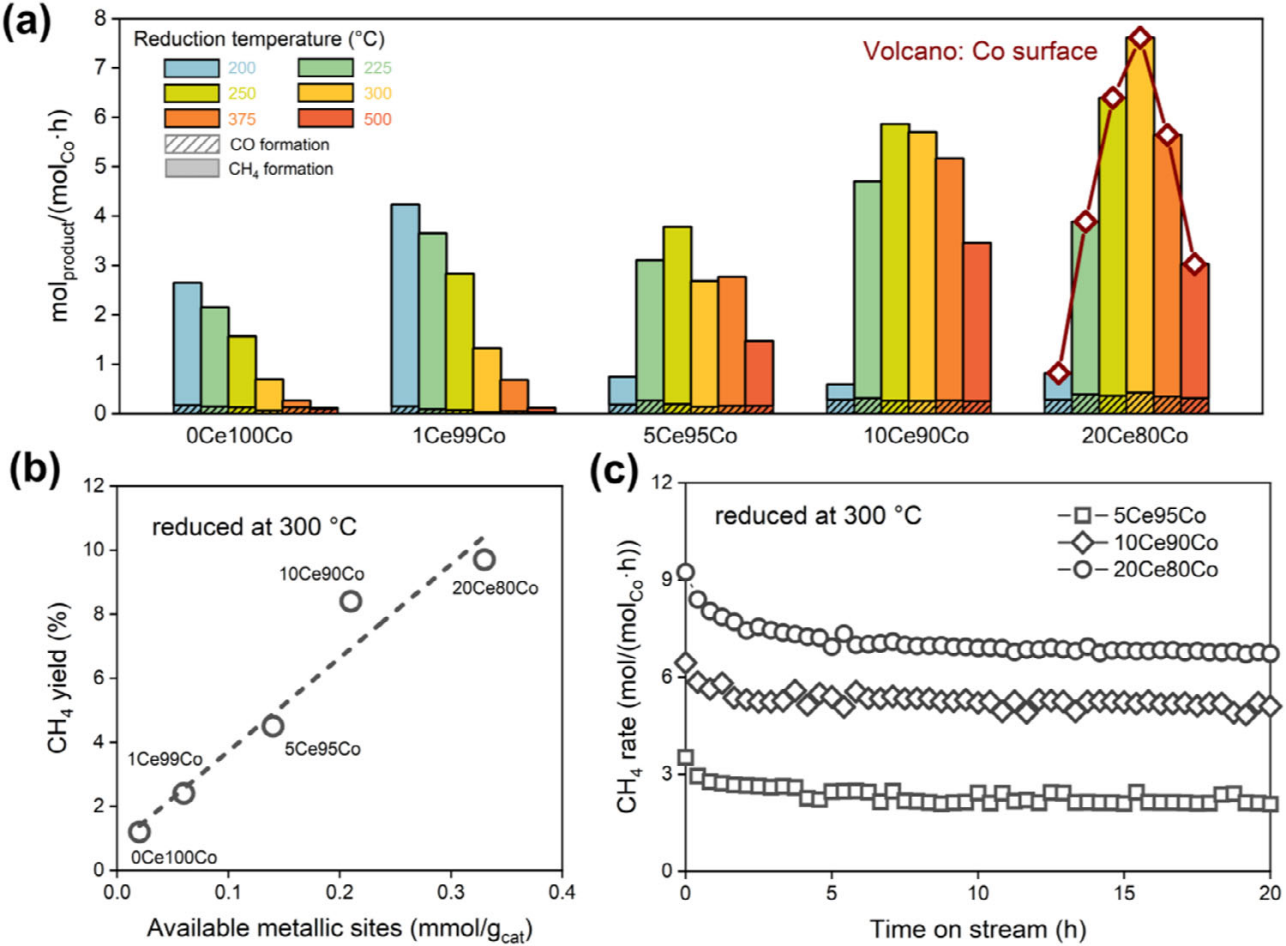
a) Catalytic CO_2_ hydrogenation performance of inverse catalysts after 3 h on stream. Reaction conditions: 200 °C, CO_2_/H_2_/He = 1/4/15, 50 mL min^−1^, samples were pre‐reduced in 10 vol% H_2_ in He (50 mL min^−1^) at different temperatures for 4 h; b) the CH_4_ yield as a function of the amount of metallic Co probed by CO chemisorption after reduced at 300 °C for 4 h and c) prolonged catalytic test of inverse catalysts after reduction at 300 °C for 4 h.

The catalytic performance of the reduced samples in CO_2_ hydrogenation was evaluated at 200 °C and 1 bar with a feed of 5 vol% CO_2_ and 20 vol% H_2_ in He (50 mL min^−1^ total flow). Before the reaction, the samples were reduced in a flow of 10 vol% H_2_ in He for 4 h at different temperatures in the 200–500 °C range. CH_4_ and CO were the dominant reaction products for all catalysts, with the selectivity to C_2_^+^ hydrocarbons below 1% (Figure S14, Supporting Information). Increasing the reduction temperature from 200 to 500 °C results in decreasing intrinsic CH_4_ formation rates from 2.50 to 0.02 mol (mol_Co_·h)^−1^ for 0Ce100Co and from 4.20 to 0.08 mol (mol_Co_·h)^−1^ for 1Ce99Co (**Figure** **4**a). CO chemisorption confirms that the metallic surface in 1Ce99Co decreases when the reduction temperature increases (Figure S15, Supporting Information). Despite the sintering, 1Ce99Co showed an appreciable increase in CO_2_ activity compared to the Ce‐free sample 0Ce100Co, suggesting that even a minimal amount of CeO_2‐x_ enhanced the CO_2_ hydrogenation performance. Further increasing the Ce content led to volcano‐like trends of the CH_4_ formation rates as a function of reduction temperature for the 10Ce90Co and 20Ce80Co catalysts. The highest CH_4_ rates were 5.60 mol (mol_Co_·h)^−1^ for 10Ce90Co after reduction at 250 °C and 7.20 mol (mol_Co_·h)^−1^ for 20Ce80Co after reduction at 300 °C. The CO formation rates were ≈0.26 and ≈0.35 mol (mol_Co_·h)^−1^ for 10Ce90Co and 20Ce80Co, independent of the reduction temperature. Notably, 10Ce90Co and 20Ce80Co exhibited low CH_4_ rates (<0.50 mol (mol_Co_·h)^−1^) and CH_4_ selectivity (<50%) after reduction at 200 °C (Figure S14, Supporting Information). At reduction temperatures above 200 °C, the CH_4_ rates in both catalysts increased by at least sevenfold, while the CH_4_ selectivity was above 90% for all catalysts. CO chemisorption revealed that both weight‐ and surface area‐normalized amounts of CO adsorbed increased with higher Ce content (Figure S16, Table S4, Supporting Information), indicating the presence of abundant metallic surface area on Co nanoparticles resulting from effective stabilization by CeO_2‐x_ clusters. The number of metallic Co sites (mmol g_cat_^−1^) strongly correlates with the CH_4_ yield for the inverse CeO_x_/Co catalysts (Figure 4b). A similar correlation was observed in 20Ce80Co after reduction at different temperatures (Figures S17 and S18, Supporting Information). Combined with the activity loss associated with the decreased metallic surface in 1Ce99Co, we infer that metallic Co sites are essential for CH_4_ formation in these catalysts.^[^ ^24^ ^]^

The reduction temperature is a critical parameter for tuning metal–support interactions to optimize catalytic performance in CO_2_ hydrogenation.^[^ ^24^, ^25^, ^26^ ^]^ However, excessively high reduction temperature can induce strong metal‐support interactions (SMSI), often leading to encapsulation of metal nanoparticles by a support‐derived overlayer, which may hinder catalytic activity.^[^ ^27^ ^]^ As discussed above, Co species in 20Ce80Co resist sintering during the H_2_ reduction step, even at a high temperature of 500 °C (Figure S11, Supporting Information). Therefore, the lower metallic surface and lower activity of 20Ce80Co obtained after reduction at 375 and 500 °C are most likely due to SMSI, rendering less Co species accessible.^[^ ^25^ ^]^ This is supported by quasi‐in situ XPS data of 20Ce80Co, which demonstrate a notable increase in the surface Ce/Co ratio from 0.57 after reduction at 300 °C to 1.05 and 1.10 after reduction at 375 and 500 °C, respectively (Figures S19–S21, Table S5, Supporting Information). This trend indicates a higher surface coverage of CeO_2‐x_ species at elevated reduction temperatures, supporting the hypothesis of SMSI overlayer formation. Notably, *quasi‐*in situ XPS shows that 20Ce80Co reduced at 300 °C contains 28% of metallic Co, while nearly all Co was reduced to the metallic form at 375 and 500 °C. Given that CH_4_ formation rates in these catalysts strongly correlate with the metallic surface, it is likely that metallic Co dominated the outermost surface layer after reduction at 300 °C.^[^ ^24^ ^]^ This can be further supported by the IR spectroscopy measurements of adsorbed CO, which revealed the characteristic features of CO adsorbed on metallic Co sites in inverse CeO_x_/Co catalysts after H_2_ reduction at 300 °C (Figure S22, Supporting Information). Furthermore, 10Ce90Co and 20Ce80Co catalysts show the highest CH_4_ STY among the prepared catalysts (Figure S23, Supporting Information). They also demonstrated higher CH_4_ STYs than any Co‐containing catalyst previously reported in the literature (Table S6, Supporting Information). The stability of the inverse catalysts was also evaluated during 20 h on stream. After the initial stabilization of ≈5 h, no significant deactivation was observed (Figure 4c; Figure S24, Supporting Information). An additional 110‐h stability test on the 10Ce90Co catalyst further confirmed its robustness in CO_2_ hydrogenation (Figure S25, Supporting Information). Moreover, *operando* XRD studies confirmed that, under harsh conditions (e.g., a high H_2_O pressure of ≈5 kPa), the 10Ce90Co catalyst was structurally stable, as no significant changes were observed in the XRD patterns during the reaction (Figure S26, Supporting Information).

### 2.4 CeO_2‐x_ Promotion of CO_2_ Activation

**Figure 5:**
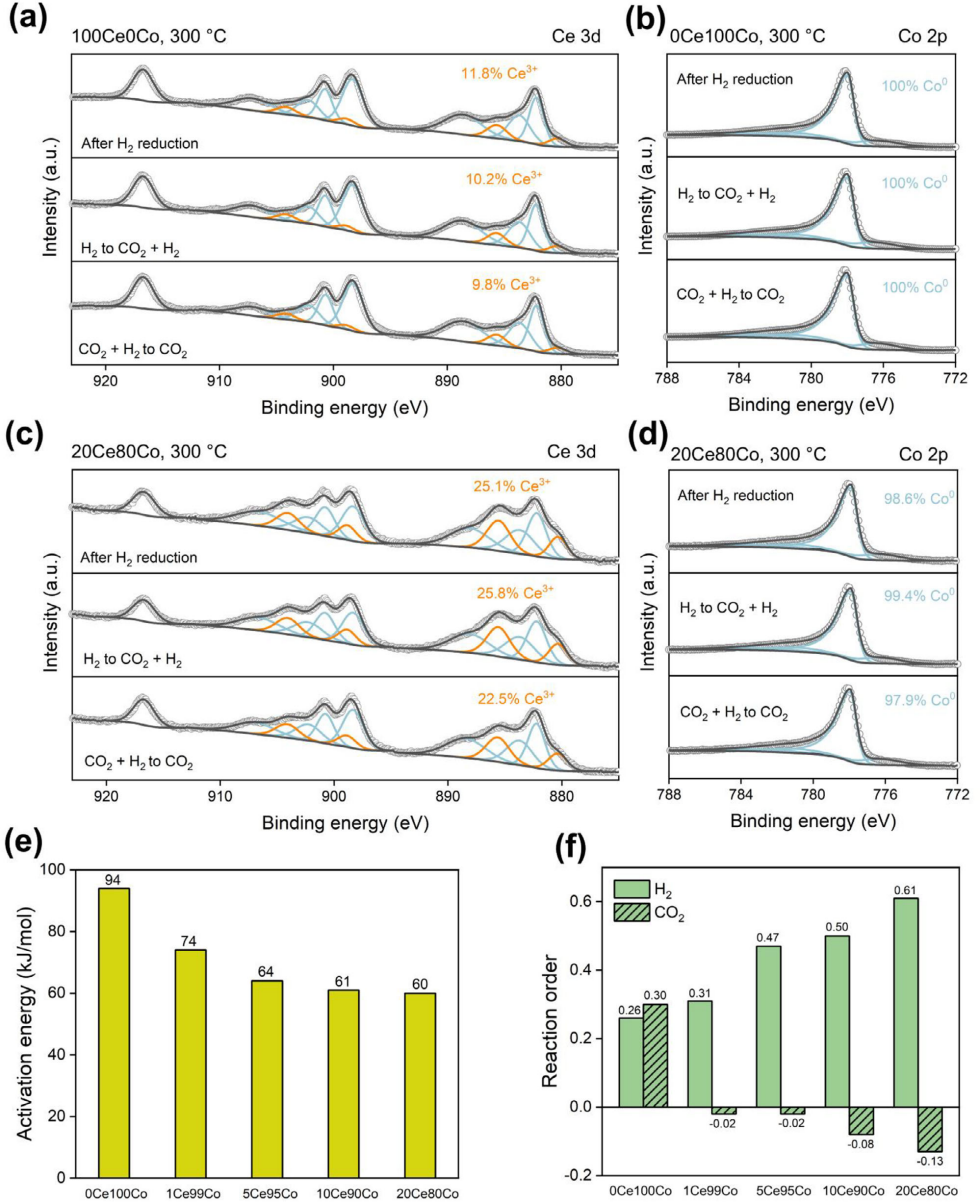
Ce 3d core‐line NAP‐XPS spectra of (a) 100Ce0Co and c) 20Ce80Co and Co 2p core‐line NAP‐XPS spectra of (b) 0Co100Co and d) 20Ce80Co; e) apparent activation energy of the catalysts. Samples were reduced in 10 vol% H_2_ in He (50 mL min^−1^) at 300 °C for 4 h. Reaction conditions: 170–200 °C, CO_2_/H_2_/He = 1/4/15, 50 mL min^−1^ and f) CO_2_ and H_2_ reaction orders. Samples were reduced in 10 vol% H_2_ in He (50 mL min^−1^) at 300 °C for 4 h. Reaction conditions: 200 °C, CO_2_ partial pressure between 0.045 and 0.06 bar, and H_2_ partial pressure between 0.12 and 0.2 bar. The total CO_2_/H_2_/He flow rate is maintained at 50 mL min^−1^.

Near‐ambient pressure (NAP)‐XPS was used to investigate the surface changes in response to the reaction conditions. After the reduction in H_2_/Ar at 500 °C overnight, the sample was cooled to 300 °C in H_2_/Ar and sequentially exposed to mixtures of CO_2_/H_2_/Ar and CO_2_/Ar. The degree of Ce reduction in 20Ce80Co was higher (25% Ce^3+^) than in the 100Ce0Co sample (12% Ce^3+^) (**Figure** **5**a,c). Moreover, the Ce^3+^ content decreased from 26% to 23% in 20Ce80Co after switching from CO_2_/H_2_ to CO_2_, unlike 100Ce0Co, where Ce^3+^ oxidation was minimal (from 10.2% to 9.8%). Our previous NAP‐XPS studies on a conventional Co/CeO_2_ catalyst also indicated minor changes in the Ce^3+^ content upon switching the gas feed from CO_2_/H_2_ to CO_2_.^[^ ^28^ ^]^ These results confirm the enhanced redox properties of the Ce species in the inverse 20Ce80Co sample. Further decreasing the Ce content leads to even stronger redox behavior in 5Ce95Co, as 41%, 37%, and 29% of Ce^3+^ species were obtained after H_2_ reduction, exposure to CO_2_/H_2_, and CO_2_, respectively (Figure S27, Supporting Information). The electronic state of Co species remained unchanged in these catalysts after H_2_ reduction, regardless of the Ce content and reaction atmosphere, as evidenced by the absence of observable changes in Co 2p XPS spectra measured under various atmospheres (Figure 5b,d).

CeO_2_‐supported metal catalysts are effective for CO_2_ hydrogenation, owing to the facile formation of abundant oxygen vacancies on CeO_2_ and unique metal‐support interactions.^[^ ^29^, ^30^ ^]^ CO_2_‐temperature programmed desorption (CO_2_‐TPD) profiles demonstrate that CO_2_ adsorption on 0Ce100Co is minimal (Figure S28, Supporting Information), while 100Ce0Co shows a high concentration of weak adsorption sites (CO_2_ desorption below 250 °C) along with small amounts of medium (250–500 °C) and strong adsorption sites (above 500 °C). In contrast, 20Ce80Co contains abundant medium CO_2_ adsorption sites, which are typically considered suitable for CO_2_ activation.^[^ ^31^, ^32^ ^]^ We propose that the observed strong Co‐CeO_2_ interactions and enhanced redox properties of CeO_2‐x_ clusters in inverse CeO_x_/Co catalysts promote CO_2_ activation and conversion. Kinetic studies support this proposition (Figure 5e,f; Figure S29, Supporting Information). The apparent activation energy (E_a_) decreased from 94 to 60 kJ mol^−1^ upon increasing the Ce content from 0 to 20%, implying a different composition of the surface adsorbed layer or a change in the reaction pathways in the inverse catalysts.^[^ ^33^ ^]^ Compared to the CO_2_ reaction order of 0.3 in 0Ce100Co, the negative CO_2_ reaction orders in the inverse catalysts indicate enhanced CO_2_ activation and high surface coverage of species derived from CO_2_.^[^ ^28^ ^]^ Consequently, converting these abundant species to CH_4_ requires a higher H_2_ pressure, explaining the higher H_2_ reaction orders for the inverse catalysts (Figure 5f).

### 2.5 Reaction Mechanism

**Figure 6:**
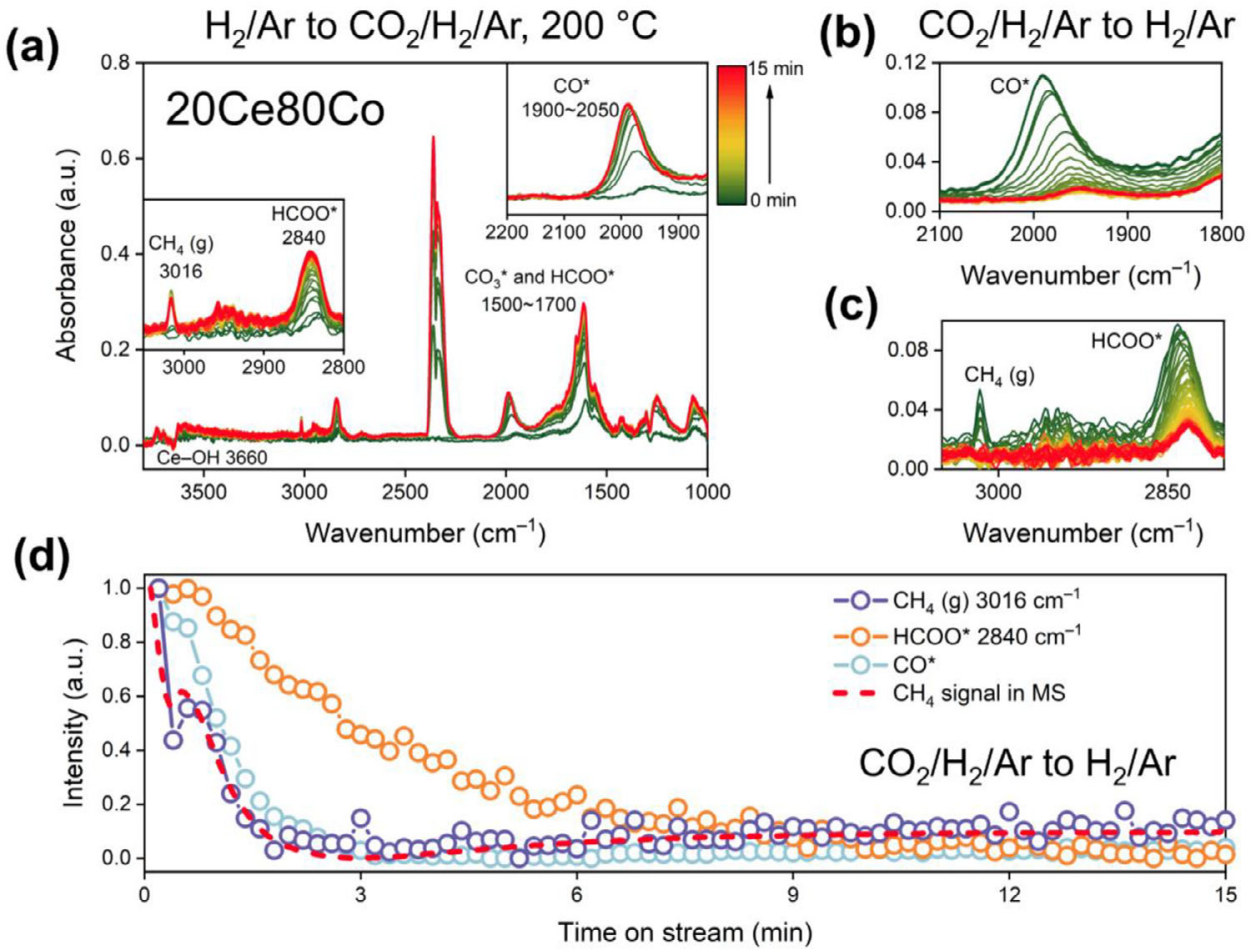
Transient *operando* DRIFTS‐MS results of 20Ce80Co: a) IR spectra after switching from H_2_/Ar (pretreatment) to CO_2_/H_2_/Ar; b, c) IR spectra of carbonyl region and C─H stretching region after switching from CO_2_/H_2_/Ar to H_2_/Ar; d) correlation between CH_4_ signal and HCOO^*^ intensity after switching from CO_2_/H_2_/Ar to H_2_/Ar. Reaction temperature: 200 °C.

Transient *operando* diffuse reflectance infrared Fourier transform spectroscopy‐mass spectrometry (DRIFT‐MS) was used to gain insight into the mechanism of CO_2_ hydrogenation on these catalysts (Scheme S1, Supporting Information). Samples were reduced at 300 °C in H_2_/Ar and then cooled to 200 °C. The gas mixture was sequentially switched from H_2_/Ar (pretreatment) to CO_2_/H_2_/Ar, H_2_/Ar, CO_2_/H_2_/Ar, and CO_2_/Ar. Upon switching from H_2_/Ar mixture to CO_2_/H_2_/Ar, no carbon‐containing adsorbates were observed for 0Ce100Co (Figure S30, Supporting Information), except for the vibrational bands of H_2_O (1300–1900 and 3600–3800 cm^−1^) and gaseous CH_4_ (3016 cm^−1^).^[^ ^34^ ^]^ An explanation can be that the strong absorption of IR light by bulk Co particles obscured the other signals.^[^ ^35^ ^]^ A large amount of gaseous CO, evident from its typical vibrational‐rotational IR spectral signature in Figure S30 (Supporting Information), was also observed upon switching from CO_2_/H_2_/Ar to CO_2_/Ar (Figure S31, Supporting Information), indicating CO_2_ readily dissociates on the metallic Co surface. However, surface carbonyl (CO^*^) species were not observed, likely due to the low surface area of this sample. Differently, the IR experiments for 20Ce80Co show the formation of carbonate (CO_3_^*^), formate (HCOO^*^), and CO^*^ species (**Figure** **6**a).^[^ ^24^, ^28^ ^]^ Transient switches between CO_2_/H_2_/Ar and H_2_/Ar demonstrate a strong correlation between the decaying gaseous CH_4_ signal and the CO^*^ intensity (Figure 6b–d; Figures S32–S34, Supporting Information), indicating that the hydrogenation of CO^*^ to CH_4_ is the primary reaction pathway (dissociative mechanism) over the inverse catalyst.^[^ ^36^ ^]^ The CO_2_ methanation mechanism on conventional Co/CeO_2_ catalysts has been extensively discussed in the literature, with HCOO^*^ frequently proposed as a reaction intermediate in the associative mechanism.^[^ ^24^, ^28^, ^37^, ^38^, ^39^, ^40^ ^]^ Our DRIFTS study on the conventional 90Ce10Co catalyst suggested that HCOO^*^ is likely a relevant intermediate (Figures S35–S38, Note S2, Supporting Information).

## 3 Conclusion

Inverse catalysts broaden the catalyst design toolkit, enabling new approaches to optimize catalytic performance. In this study, we employed FSP to synthesize uniform inverse CeO_x_/Co catalysts for CO_2_ hydrogenation. The resulting Co nanoparticles were effectively stabilized by small CeO_2‐x_ clusters, which enhanced metal‐support interactions and conferred strong resistance to sintering during H_2_ reduction. Beyond structural stabilization, the highly reducible CeO_2‐x_ clusters facilitated CO_2_ adsorption and activation. The reduction temperature was pivotal in tuning Co‐CeO_2_ interactions to optimize catalytic activity. Notably, moderate H_2_ reduction temperatures led to the highest metallic Co surface area and significantly enhanced CH_4_ formation rates. In contrast, higher reduction temperatures induced strong metal‐support interactions (SMSI), which diminished catalytic activity. Mechanistic studies revealed that CO^*^ species were the predominant intermediates on the inverse catalysts, which were readily hydrogenated to CH_4_, while HCOO^*^ species appeared to act as spectators. In comparison, HCOO^*^ was the most likely reaction intermediate in conventional Co/CeO_2_ catalysts. These findings offer valuable insights into the structure‐performance relationships in inverse CeO_x_/Co catalysts, guiding the rational design of improved materials for CO_2_ hydrogenation and related catalytic processes.

## Conflict of Interest

The authors declare no conflict of interest.

## Supporting information



Supporting Information

## Data Availability

The data that support the findings of this study are available from the corresponding author upon reasonable request.

## References

[1] A. Beck , M. Zabilskiy , M. A. Newton , O. Safonova , M. G. Willinger , J. A. van Bokhoven , Nat. Catal. 2021, 4, 488.

[2] Z. Luo , C. Liu , A. Radu , D. F. de Waard , Y. Wang , B. de Bueren , T. J. , P. D. Kouris , M. D. Boot , J. Xiao , H. Zhang , R. Xiao , J. S. Luterbacher , E. J. M. Hensen , Nat. Chem. Eng. 2024, 1, 61.

[3] Z. Cen , X. Han , L. Lin , S. Yang , W. Han , W. Wen , W. Yuan , M. Dong , Z. Ma , F. Li , Y. Ke , J. Dong , J. Zhang , S. Liu , J. Li , Q. Li , N. Wu , J. Xiang , H. Wu , L. Cai , Y. Hou , Y. Cheng , L. L. Daemen , A. J. Ramirez‐Cuesta , P. Ferrer , D. C. Grinter , G. Held , Y. Liu , B. Han , Nat. Chem. 2024, 16, 871. 38594366 10.1038/s41557-024-01506-z PMC11164678

[4] P. Munnik , P. E. De Jongh , K. P. De Jong , Chem. Rev. 2015, 115, 6687. 26088402 10.1021/cr500486u

[5] T. W. Hansen , A. T. Delariva , S. R. Challa , A. K. Datye , Acc. Chem. Res. 2013, 46, 1720. 23634641 10.1021/ar3002427

[6] S. Higashi , K. Miwa , M. Yokoi , N. Takahashi , S. Kosaka , D. Urushihara , T. Asaka , D. Teschner , J. Vélez , D. Cruz , R. Blume , R. Schloegl , B. R. Cuenya , A. Knop‐Gericke . Increased Catalytic Activity of the Sabatier Reaction for Space Applications, 09 July 2024, PREPRINT (Version 1) available at Research Square, 10.21203/rs.3.rs-4670357/v1.

[7] J. Zhang , J. W. Medlin , Surf. Sci. Rep. 2018, 73, 117.

[8] J. A. Rodriguez , S. Ma , P. Liu , J. Hrbek , J. Evans , M. Pérez , Science 2007, 318, 1757. 18079397 10.1126/science.1150038

[9] J. Fu , S. Liu , W. Zheng , R. Huang , C. Wang , A. Lawal , K. Alexopoulos , S. Liu , Y. Wang , K. Yu , J. A. Boscoboinik , Y. Liu , X. Liu , A. I. Frenkel , O. A. Abdelrahman , R. J. Gorte , S. Caratzoulas , D. G. Vlachos , Nat. Catal. 2022, 5, 144.

[10] Y. Li , Y. Zhang , K. Qian , W. Huang , ACS Catal. 2022, 12, 1268.

[11] V. Muravev , A. Parastaev , Y. Van Den Bosch , B. Ligt , N. Claes , S. Bals , N. Kosinov , E. J. M. Hensen , Science 2023, 380, 1174. 37319196 10.1126/science.adf9082

[12] S. D. Senanayake , P. J. Ramírez , I. Waluyo , S. Kundu , K. Mudiyanselage , Z. Liu , Z. Liu , S. Axnanda , D. J. Stacchiola , J. Evans , J. A. Rodriguez , J. Phys. Chem. C 2016, 120, 1778.

[13] J. A. Rodríguez , J. Hrbek , Surf. Sci. 2010, 604, 241.

[14] C. Song , J. Liu , R. Wang , X. Tang , K. Wang , Z. Gao , M. Peng , H. Li , S. Yao , F. Yang , H. Lu , Z. Liao , X.‐D. Wen , D. Ma , X. Li , L. Lin , Nat. Chem. Eng. 2024, 1, 638.

[15] Y. Zang , T. Wei , J. Qu , F. Gao , J. Gu , X. Lin , S. Zheng , Appl. Surf. Sci. 2025, 685, 161945.

[16] J. Tian , P. Zheng , T. Zhang , Z. Han , W. Xu , F. Gu , F. Wang , Z. Zhang , Z. Zhong , F. Su , G. Xu , Appl. Catal. B 2023, 339, 123121.

[17] X. Tang , C. Song , H. Li , W. Liu , X. Hu , Q. Chen , H. Lu , S. Yao , X. N. Li , L. Lin , Nat. Commun. 2024, 15, 3115. 38600102 10.1038/s41467-024-47403-4 PMC11006838

[18] A. Parastaev , V. Muravev , E. Huertas Osta , A. J. F. van Hoof , T. F. Kimpel , N. Kosinov , E. J. M. Hensen , Nat. Catal. 2020, 3, 526.

[19] L. Wu , H. J. Wiesmann , A. R. Moodenbaugh , R. F. Klie , Y. Zhu , D. O. Welch , M. Suenaga , Phys. Rev. B 2004, 69, 125415.

[20] X. Hao , A. Yoko , C. Chen , K. Inoue , M. Saito , G. Seong , S. Takami , T. Adschiri , Y. Ikuhara , Small 2018, 14, 1802915. 10.1002/smll.201802915 30260567

[21] S. Zhang , Y. Li , J. Huang , J. Lee , D. H. Kim , A. I. Frenkel , T. Kim , J. Phys. Chem. C 2019, 123, 7166.

[22] T. Y. Chen , J. Su , Z. Zhang , C. Cao , X. Wang , R. Si , X. Liu , B. Shi , J. Xu , Y. F. Han , ACS Catal. 2018, 8, 8606.

[23] W. Li , X. Nie , H. Yang , X. Wang , F. Polo‐Garzon , Z. Wu , J. Zhu , J. Wang , Y. Liu , C. Shi , C. Song , X. Guo , Appl. Catal. B 2022, 315, 121529.

[24] A. Parastaev , V. Muravev , E. H. Osta , T. F. Kimpel , J. F. M. Simons , A. J. F. van Hoof , E. Uslamin , L. Zhang , J. J. C. Struijs , D. B. Burueva , E. V. Pokochueva , K. V. Kovtunov , I. V. Koptyug , I. J. Villar‐Garcia , C. Escudero , T. Altantzis , P. Liu , A. Béché , S. Bals , N. Kosinov , E. J. M. Hensen , Nat. Catal. 2022, 5, 1051.

[25] M. Xu , X. Qin , Y. Xu , X. Zhang , L. Zheng , J.‐X. Liu , M. Wang , X. Liu , D. Ma , Nat. Commun. 2022, 13, 6720. 36344530 10.1038/s41467-022-34463-7 PMC9640681

[26] Z. Shi , Q. Tan , C. Tian , Y. Pan , X. Sun , J. Zhang , D. Wu , J. Catal. 2019, 379, 78.

[27] B. Han , Y. Guo , Y. Huang , W. Xi , J. Xu , J. Luo , H. Qi , Y. Ren , X. Liu , B. Qiao , T. Zhang , Angew. Chem., Int. Ed. 2020, 59, 11824. 10.1002/anie.202003208 32302045

[28] J. J. C. Struijs , V. Muravev , M. A. Verheijen , E. J. M. Hensen , N. Kosinov , Angew. Chem., Int. Ed. 2022, 135, 202214864. 10.1002/anie.202214864 PMC10107782 36464648

[29] K. Chang , H. Zhang , M. J. Cheng , Q. Lu , ACS Catal. 2020, 10, 613.

[30] J. Ma , Q. Jiang , S. Li , W. Chu , H. Qian , S. Perathoner , G. Centi , Y. Liu , Chem. Eng. J. 2024, 479, 147453.

[31] Y. Bian , C. Xu , X. Wen , L. Xu , Y. Cui , S. Wang , C. e. Wu , J. Qiu , G. Cheng , M. Chen , Fuel 2023, 331, 762.

[32] R. P. Ye , Q. Li , W. Gong , T. Wang , J. J. Razink , L. Lin , Y. Y. Qin , Z. Zhou , H. Adidharma , J. Tang , A. G. Russell , M. Fan , Y. G. Yao , Appl. Catal. B 2020, 268, 100854.

[33] S. Kattel , P. Liu , J. G. Chen , J. Am. Chem. Soc. 2017, 139, 9739. 28650651 10.1021/jacs.7b05362

[34] J. Weiß , Q. Yang , U. Bentrup , E. V. Kondratenko , A. Brückner , C. Kubis , ChemCatChem 2022, 14, 576.

[35] J. M. Olinger , P. R. Griffiths , Anal. Chem. 1998, 60, 2427.

[36] J. F. M. Simons , T. J. de Heer , R. C. J. van de Poll , V. Muravev , N. Kosinov , E. J. M. Hensen , J. Am. Chem. Soc. 2023, 145, 3787. 10.1021/jacs.3c04284 PMC10515628 37677099

[37] K. Deng , L. Lin , N. Rui , D. Vovchok , F. Zhang , S. Zhang , S. D. Senanayake , T. Kim , J. A. Rodriguez , Catal. Sci. Technol. 2020, 10, 6468.

[38] B. Li , F. Wang , K. Li , P. Ning , M. Chen , C. Zhang , J. Rare Earths 2023, 41, A5378.

[39] X. Zou , Z. Shen , X. Li , Y. Cao , Q. Xia , S. Zhang , Y. Liu , L. Jiang , L. Li , L. Cui , Y. Wang , J. Colloid Interface Sci. 2022, 620, 3236. 10.1016/j.jcis.2022.04.001 35421755

[40] T. H. Nguyen , H. B. Kim , E. D. Park , Catalysts 2022, 12, 212.

