# Supplementary material for: Strong Stabilization of Co Nanoparticles by CeO2‐x Clusters in Inverse CeOx/Co Catalysts for Enhanced CO2 Methanation
Source: Adv Mater. 2025 Sep 25;38(2):e10593. doi: 10.1002/adma.202510593 (PMC12783866; doi:10.1002/adma.202510593)
Supplement: Supplementary file 1 — Supporting Information [file ADMA-38-e10593-s001.docx]

**Supporting information**

**Strong Stabilization of Co Nanoparticles by CeO_2-x_ Clusters in Inverse CeO_x_/Co Catalysts for Enhanced CO_2_ Methanation**

Yu Gao, Valery Muravev, Yonghui Fan, Hao Zhang, Jorden Wagemakers, Alexander Parastaev, Nikolay Kosinov* and Emiel J. M. Hensen*

Laboratory of Inorganic Materials and Catalysis, Department of Chemical Engineering and Chemistry, Eindhoven University of Technology, 5600 MB Eindhoven, The Netherlands.

*Email: [n.a.kosinov@tue.nl](mailto:n.a.kosinov@tue.nl); e.j.m.hensen@tue.nl

**Methods**

**Chemicals**

Cobalt acetylacetonate (Sigma Aldrich, 97%), cerium acetate hydrate (Tokyo Chemical Industry, >98%), acetic acid (Sigma Aldrich, glacial, ≥99%) and 2-ethylhexanoic acid (Sigma Aldrich, 99%) were used in this study to prepare all catalysts.

**Catalyst preparation**

Flame spray pyrolysis with a commercial TETHIS NPS10 apparatus was used to prepare catalysts with different Ce and Co amounts. The precursor solutions were prepared by dissolving appropriate amounts of cobalt acetylacetonate and cerium acetate in a 1:1 (vol%) solvent mixture of acetic and 2-ethylhexanoic acid at room temperature. The total metal (Co and Ce) concentration was kept at 0.15 M. The solution was then injected into the nozzle of the FSP setup using a syringe pump at a rate of 5 mL/min. CH_4_ (1.5 L/min) and O_2_ (3 L/min) gases were fed into the setup to generate a flame with an O_2_ dispersion flow (5 L/min) around it. The as-prepared sample was collected using a quartz fiber filter placed on top of the setup. The as-prepared samples are denoted as xCeyCo, in which *x* and *y* represent the molar fractions (%) of Ce and Co (*x* + *y* = 100, *x* = 0, 1, 5, 10, 20, 50, 90, and 100), respectively.

**Characterization**

**X-ray diffraction (XRD).** XRD patterns of as-prepared samples were recorded at the ID-31 beamline of the European Synchrotron Radiation Facility (ESRF, Grenoble, France). The incident X-ray energy was λ = 0.1653 Å. Before the measurements, samples were sealed in Kapton tubes with an external diameter of 3 mm diameter and a wall thickness of 0.03 mm. ***In situ* XRD** patterns were collected at the P02.1 beamline of the Deutsches Elektronen-Synchrotron (DESY, Hamburg, Germany) with an incident X-ray energy of λ = 0.2073 Å. Before the measurements, 10 mg of sample was loaded into a quartz tube (100 mm length, 3 mm external diameter, and 100 μm thickness) and kept in place by two layers of quartz wool. Then, the tube was horizontally mounted into a home-made reactor and heated by a gas blower in 10 vol% H_2_ in He (50 mL/min) from 30 °C to 500 °C, followed by a dwell of 1 h. XRD patterns were recorded every 30 s during temperature-programmed H_2_ reduction.

**Inductively coupled plasma optical emission spectrometry (ICP-OES).** ICP-OES elemental analysis was done using an AMETEK ICP optical emission spectrometer (Spectroblue). Aliquots of catalyst samples were dissolved in a mixture of 4 mL H_2_SO_4_ (65 wt%) and 2 mL HNO_3_ (97 wt%), assisted by microwave.

**X-ray photoelectron spectroscopy** **(XPS).** XPS spectra of as-prepared samples were recorded on a K-Alpha X-ray photoelectron spectrometer (Thermo Scientiﬁc) with an Al anode (1486.68 eV). The samples were dispersed on a double-sided carbon tape.. The CasaXPS software was used for data processing, and the C 1s peak of adventitious carbon was used for energy calibration (binding energy set to 284.8 eV) .

**Quasi*-in situ* X-ray photoelectron spectroscopy** **(XPS).** Quasi*-in situ* XPS results were obtained on a Kratos AXIS Ultra 600 spectrometer with a monochromatic Al Kα X-ray source (1486.68 eV). An amount of 10 mg catalyst was pressed into a self-supporting wafer and placed onto a sample holder. The sample was reduced in a high-temperature reaction cell (Kratos, WX-530) using 10 vol% H_2_ in Ar (50 mL/min) at different temperatures for 4 h. After evacuation, the sample was transferred to the analysis chamber. The XPS spectra were energy-calibrated by the U^III^ component in Ce 3d core-line spectrum with a characteristic binding energy of 916.7 eV. The CasaXPS software was used for data processing.

**Near-ambient pressure (NAP)-XPS.** NAP-XPS results were recorded on a SPECS PHOIBOS NAP XPS system, equipped with a PHOIBOS 150 hemispherical energy analyzer and a an Al anode (1486.68 eV). An amount of 70 mg of catalyst was pressed into a self-supporting wafer and placed onto a stainless-steel sample holder. The sample was reduced in a flow of 1 mbar H_2_ and 2 mbar Ar at 500 °C overnight, followed by cooling to 300 °C. Two different reaction mixtures were used: (i) a mixture of 0.3 mbar CO_2_, 1.2 mbar H_2_, and 1.5 mbar Ar (CO_2_/H_2_/Ar feed), and (ii) a mixture of 2 mbar CO_2_ and 1 mbar Ar (CO_2_/Ar feed). XPS spectra were energy-calibrated by setting the U^III^ component in the Ce 3d core-line spectrum to its characteristic binding energy of 916.7 eV. The CasaXPS software was used for data processing.

**X-ray absorption near-edge structure (XANES).** XANES spectra of as-prepared samples were collected at the ROCK beamline, SOLEIL Synchrotron (Saint-Aubin, France). Samples were either pressed into a self-supporting wafer (diluted with cellulose) or loaded inside a quartz capillary (100 mm length and 10 μm thickness, diluted with boron nitride). Data was collected at the Co *K*-edge (7708.9 eV) and the Ce *L*_3_-edge (5723.4 eV) in transmission mode. A Co foil was used for energy calibration. The Larch software package was used to normalize the XANES spectra and to carry out linear combination fitting (LCF).

**CO chemisorption.** CO chemisorption experiments were carried out on a Micromeritics ASAP 2010C instrument. Before chemisorption measurements, the sample was dehydrated at 110 °C and reduced in H_2_ at various temperatures (rate 10 °C/min to 200-500 °C, followed by a dwell of 4 h). The sample was then evacuated for 1 h at the final temperature. CO chemisorption measurements were performed at 35 °C. The amount of adsorbed CO was obtained by extrapolating the irreversible adsorption isotherm to zero pressure. The metallic surface was calculated assuming a hemispherical shape of the particles and a CO/Co_surface_ stoichiometry of 1.5.

**CO IR spectroscopy.** CO IR spectroscopy was carried out on a Bruker Vertex 70v FTIR spectrometer. Before measurements, approximately 5 mg of catalyst diluted with SiO_2_ was pressed into a thin self-supporting wafer (diameter 13 mm) and placed in a controlled-environment transmission IR cell. The sample was reduced in a flow of 10 vol% H_2_ in He at 300 °C for 4 h, followed by cooling to 50 °C and evacuation until the pressure was below 10^−6^ mbar. Before dosing CO, a background spectrum was collected. The CO pressure was stepwise increased from 0 to 1 mbar by dosing small CO pulses. A total of 64 scans were averaged to obtain an IR spectrum, with a resolution of 4 cm^−1^ in the 400~4000 cm^−1^ range.

**N_2_ physisorption.** N_2_ physisorption tests were performed on a Micromeritic 3FLEX apparatus at −196 °C. Before physisorption measurements, the reduced samples were pretreated at 300 °C under vacuum for 2 h. The specific surface area was determined by the Brunauer-Emmett-Teller (BET) method.

**H_2_ temperature-programmed reduction (H_2_-TPR).** H_2_-TPR experiments were conducted using a Micromeritics ASAP II 2920 instrument. An amount of 50 mg of catalyst was loaded into a tubular quartz reactor. After dehydration in He at 300 °C for 1 h, the temperature was cooled to 40 °C. H_2_ reduction was performed in a flow of 4 vol % H_2_ in N_2_, heating to 600 °C at a rate of 10 °C/min.

**CO_2_ temperature-programmed desorption (CO_2_-TPD).** CO_2_-TPD experiments were performed in a fixed-bed microreactor connected to a mass spectrometer (MS, Pfeiffer Omnistar). An amount of 10 mg of catalyst was reduced in a 50 mL/min flow of 10 vol% H_2_ in He at 300 °C for 4 h, followed by cooling to 50 °C. The catalyst was purged in a He flow, until no H_2_ (*m/z* = 2) was detected in the effluent by a mass spectrometer. The reduced sample was exposed to a flow of 10 vol% CO_2_ in He (50 mL/min) for 30 min, followed by a switch to a He flow to purge the catalyst until no CO_2_ (*m/z* = 44) was detected in the effluent. Finally, CO_2_ desorption was studied by heating the catalyst at a rate of 10 °C/min from 50 to 700 °C in a He flow (50 mL/min).

**Transmission electron microscopy (TEM).** TEM (TEM), high-angle annular dark-field scanning transmission electron microscopy (HAADF-STEM) and scanning transmission electron microscopy-energy dispersive X-ray spectroscopy (STEM-EDX) were carried out on a FEI cubed Titan Cs-corrected transmission electron microscope operating at 300 KV. Sample preparation involved the dispersion of the catalyst in absolute ethanol by ultrasonic treatment, followed by deposition onto a holey carbon film on a Cu grid.

***Operando* diffuse reflectance infrared Fourier transform spectroscopy (DRIFTS).** *Operando* DRIFTS experiments were conducted at 1 bar using a Vertex 70v infrared spectrometer (Bruker) with an MCT detector. Before IR measurements, an amount of 40 mg of catalyst was reduced in a flow of 10 vol% H_2_ in Ar (50 mL/min) at 300 °C for 4 h, followed by cooling to 200 °C. A background IR spectrum was collected, followed by switching the gas flow to a CO_2_/H_2_/Ar (2.5/10/37.5 mL/min) mixture. The effluent gas was analyzed by a mass spectrometer (Pfeiffer Omnistar). The detailed procedure of the experiment is presented in **Scheme S1**.

**Catalytic performance evaluation**

The catalytic performance in CO_2_ hydrogenation was evaluated in a 10-tube parallel high-throughput setup at 1 bar. Typically, 10 mg of catalyst (125~250 um) diluted with 290 mg SiC was loaded into a quartz reactor. Before starting the reaction, the catalyst was reduced in a 50 mL/min flow of 10 vol% H_2_ in He at various temperatures (200-500 °C) for 4 h. Then, the temperature was lowered to 200 °C and the feed was switched to the H_2_/CO_2_/He (10/2.5/37.5 mL/min) reaction mixture. The effluent gas was sampled and analyzed by an online gas chromatograph (Interscience, CompactGC), equipped with three columns and three detectors (Rt-Q Bond with an FID, Rt-U Bond with a TCD, and Molsieve 5A with a TCD). The CO_2_ conversion and the product selectivity and formation rates were calculated as follows:

CO_2_ conversion (%), $\text{X =}\frac{\text{F}\left( \text{CO}_{\text{2 }}\text{in} \right)\text{ }\text{–}\text{ }\text{F}\left( \text{CO}_{\text{2 }}\text{out} \right)}{\text{F}\text{ }\left( \text{CO}_{\text{2 }}\text{in} \right)}\text{ ×100\% (1)}$

Selectivity (%), $\text{S}_{\text{i}}\text{= }\frac{\text{F}_{\text{i}}}{\text{}\text{F}_{\text{i}}}\text{ × 100\% }\text{ }\text{(2)}$

Formation rate (mol_product_/(mol_Co_·h)), $\text{r(}\text{i}\text{)=}\frac{\text{F(}\text{i}\text{)}}{\text{V}_{\text{m}}\text{×}\text{N}_{\text{i}\text{C}\text{o}}\text{ }}\text{ }\text{ }\text{ }\text{ }\text{(}\text{3}\text{)}$

where F(CO_2_ in) and F(CO_2_ out) are the CO_2_ flow rates (mL/h) of the influent and effluent gas, respectively. F(*i*) represents the flow rate (mL/h) of component *i* in the effluent gas. V_m_ is the molar volume of ideal gas at standard temperature and pressure, and N*_i_*_Co_ is the Co content of the sample. For kinetic studies, a temperature range of 170-200 °C, a CO_2_ partial pressure range of 0.045-0.06 bar (at 200 °C), and a H_2_ partial pressure range of 0.12-0.2 bar (at 200 °C) were used to determine the apparent activation energy, CO_2_ reaction orders, and H_2_ reaction orders, respectively.

**Table S1.** Composition of the samples determined by ICP elemental analysis.

| Samples | Co wt% measured | Ce wt% measured | Atomic Co/(Co+Ce)  (%, nominal) | Atomic Co/(Co+Ce)  (%, measured) |
| --- | --- | --- | --- | --- |
| 0Ce100Co | 73.4 | 0 | 100 | 100 |
| 1Ce99Co | 62.8 | 1.6 | 99 | 98.9 |
| 5Ce95Co | 58.8 | 6.7 | 95 | 95.4 |
| 10Ce90Co | 52.6 | 11.0 | 90 | 91.9 |
| 20Ce80Co | 41.9 | 23.3 | 80 | 81.1 |
| 50Ce50Co | 20.5 | 43.2 | 50 | 53.0 |
| 90Ce10Co | 3.6 | 69.4 | 10 | 10.9 |
| 0Ce100Co | 0 | 81.4 | 0 | 0 |


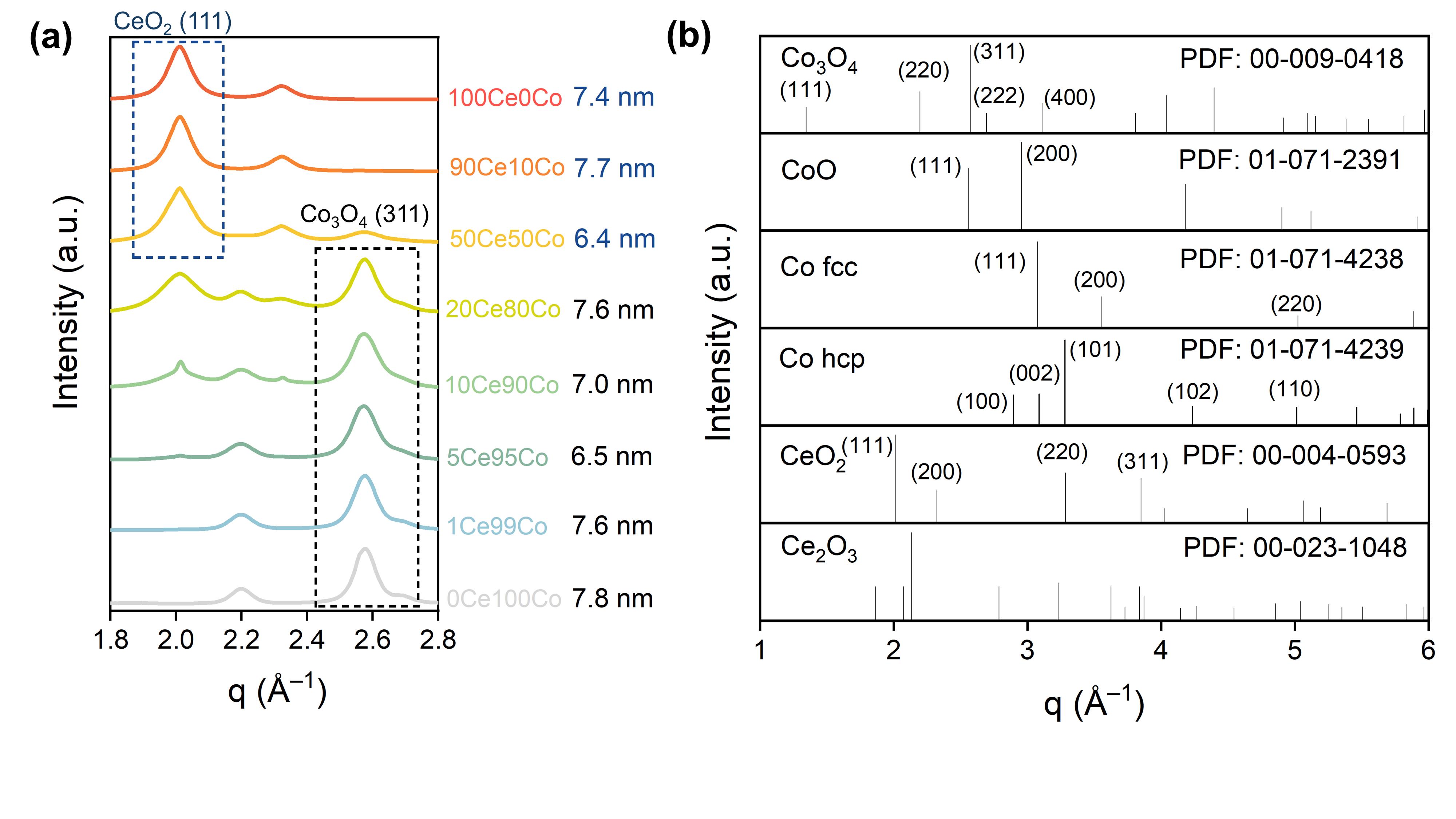


**Figure S1.** (a) Synchrotron XRD patterns of as-prepared samples. The Scherrer equation was used to calculate the particle size of the crystallites, with the shape factor K set to 0.94. This value was chosen based on the assumption that the crystallites are spherical in shape with cubic symmetry. The CeO_2_(111) diffraction peak was chosen to determine the particle sizes of 100Ce0Co, 90Ce10Co and 50Ce50Co. The Co_3_O_4_(311) diffraction peak was chosen to determine the particle sizes of 20Ce80Co, 10Ce90Co, 5Ce95Co, 1Ce99Co and 0Ce100Co; (b) XRD patterns of reference compounds from the PDF database.

**Table S2.** Linear combination fitting results of Ce L_3_-edge and Co K-edge XANES spectra of as-prepared samples. CeO_2_ and Ce(NO_3_)_3_ were used as the Ce^4+^ and Ce^3+^ references, respectively.

| Sample | Ce^3+^ (%) | Ce^4+^ (%) | Co_3_O_4_ (%) | CoO (%) |
| --- | --- | --- | --- | --- |
| 0Ce100Co | − | − | 100 | 0 |
| 1Ce99Co | 47.7 | 52.3 | 100 | 0 |
| 5Ce95Co | 26.1 | 73.9 | 100 | 0 |
| 10Ce90Co | 6.8 | 93.2 | 100 | 0 |
| 20Ce80Co | 3.8 | 96.2 | 100 | 0 |
| 50Ce50Co | 3.5 | 96.5 | 100 | 0 |
| 90Ce10Co | 2.2 | 97.8 | 78.2 | 21.8 |
| 100Ce0Co | 3.2 | 96.8 | − | − |


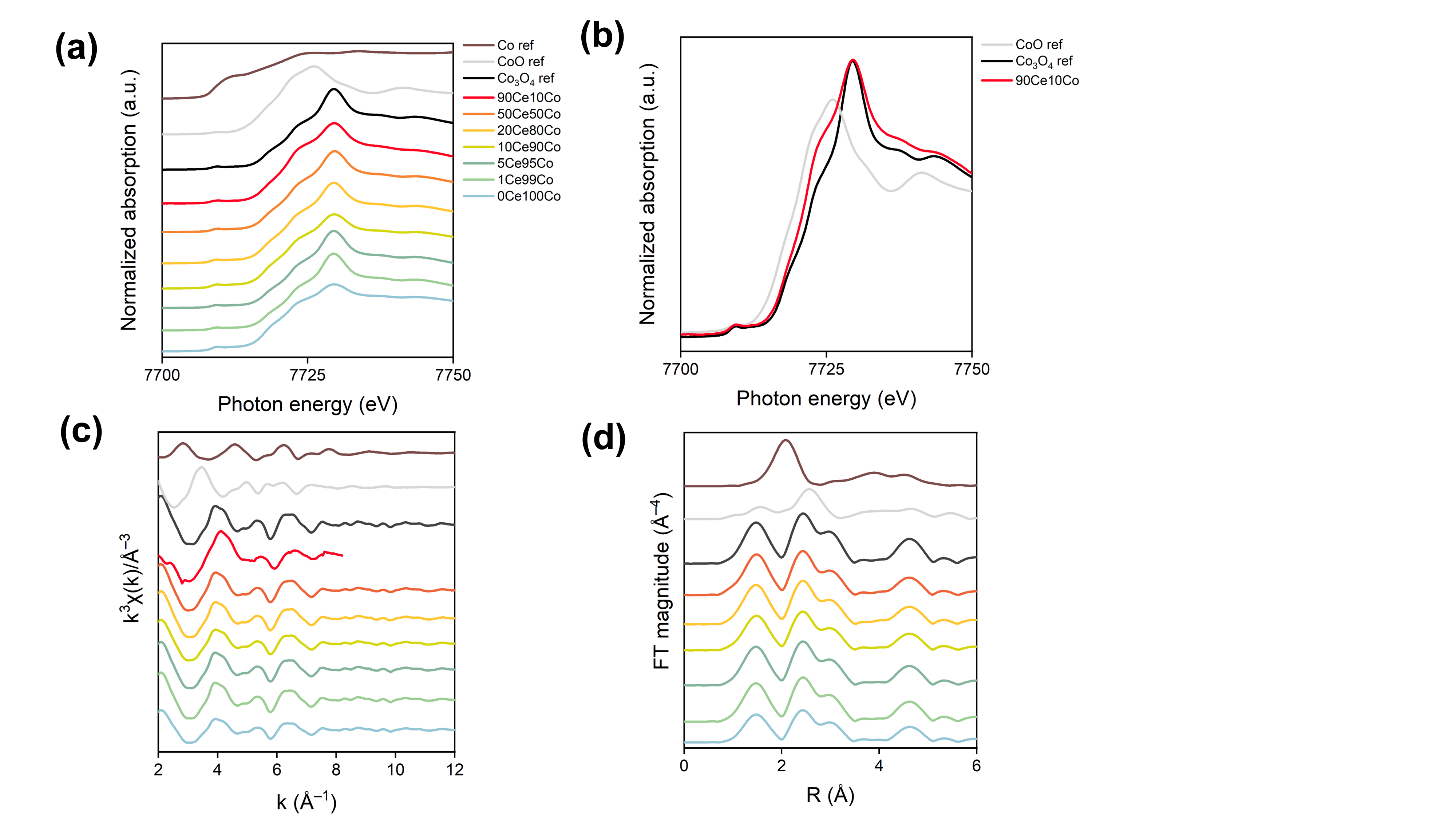


**Figure S2.** (a, b) Co K-edge XANES spectra; (c) Co K-edge k^3^-weighted EXAFS and (d) their Fourier transforms of as-prepared samples and references. Data for 0Ce100Co, 10Ce90Co, 20Ce80Co, 50Ce50Co and 90Ce10Co were measured by diluting the sample (sieve fraction 125~250 μm) with boron nitride in a quartz capillary. The 1Ce99Co, 5Ce95Co and the Co references were measured using self-supported wafers (note that the FT-EXAFS of 90Ce10Co is omitted in panel d due to the low signal-to-noise ratio at high k values).


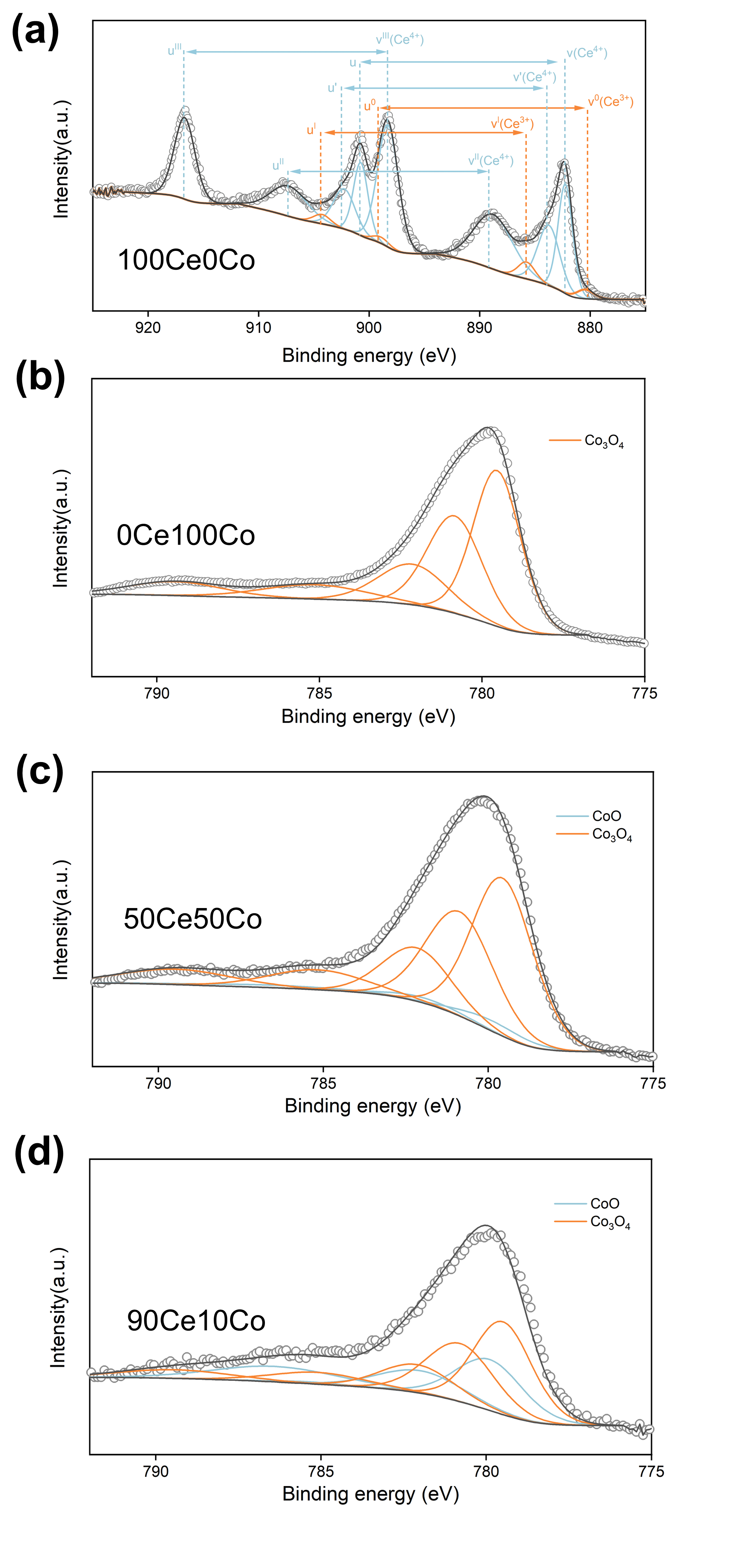


**Figure S3.** Representative fit of (a) Ce 3d core-line XPS spectra with 100Ce0Co as the reference and Co 2p core-line XPS spectra with (b) 0Ce100Co, (c) 50Ce50Co and (d) 90Ce10Co as the references (Ce 3d XPS fit model by Skála et al. and Kato et al,^[1,2]^ and the Co 2p fit model by Biesinger et al.^[3]^)

***Table S3****. XPS spectral fitting parameters for 0Ce100Co, 90Ce10Co, and Co metal.*

| Component | Line shape | FWHM | FWHM constraint | Position (eV) | Position  constraint (eV) |
| --- | --- | --- | --- | --- | --- |
| v^0^ (Ce^3+^) | GL(30) | 1.8 | 1.3, 2.4 | 880.5 | 880.7, 880.4 |
| u^0^ (Ce^3+^) | GL(30) | 1.8 | 1.8, 2.4 | 899.0 | 899.2, 898.9 |
| v^I^ (Ce^3+^) | GL(30) | 2.4 | 1.8, 2.4 | 885.6 | 885.9, 885.2 |
| u^I^ (Ce^3+^) | GL(30) | 2.4 | 1.8, 2.4 | 904.2 | 904.4, 903.7 |
| v (Ce^4+^) | GL(30) | 1.6 | 0.4, 10.2 | 882.2 | 932.1, 876.1 |
| u (Ce^4+^) | GL(30) | 1.6 | 0.4, 10.2 | 900.8 | 933.6, 877.6 |
| v' (Ce^4+^) | GL(30) | 2.4 | 0.6, 15.4 | 883.7 | 932.1, 876.1 |
| u' (Ce^4+^) | GL(30) | 2.4 | 0.6, 15.4 | 902.3 | 933.6, 877.6 |
| v^II^ (Ce^4+^) | GL(30) | 4.1 | 3.5, 4.5 | 888.6 | 932.1, 876.1 |
| u^II^ (Ce^4+^) | GL(30) | 4.1 | 3.5, 4.5 | 907.1 | 933.6, 877.6 |
| v^III^ (Ce^4+^) | GL(30) | 2.2 | 0.5, 13.1 | 898.3 | 932.1, 876.1 |
| u^III^ (Ce^4+^) | GL(30) | 2.2 | 0.5, 13.1 | 916.7 | 932.1, 876.1 |
| Co_3_O_4__1 | GL(30) | 2.1 | 1.5, 2.2 | 779.5 | 779.7, 779.3 |
| Co_3_O_4__2 | GL(30) | 2.4 | 1.6, 2.4 | 780.8 | 781.0, 780.6 |
| Co_3_O_4__3 | GL(30) | 2.6 | 1.9, 2.6 | 782.1 | 782.3, 781.9 |
| Co_3_O_4__4 | GL(30) | 3.3 | 3.3, 4.5 | 785.1 | 785.3, 784.9 |
| Co_3_O_4__5 | GL(30) | 4.0 | 3.1, 4.0 | 789.4 | 789.6, 789.2 |
| CoO_1 | GL(30) | 2.4 | 2.2, 2.4 | 779.9 | 780.1, 779.7 |
| CoO_2 | GL(30) | 3.0 | 2.3, 3.0 | 782.0 | 782.2, 781.8 |
| CoO_3 | GL(30) | 2.3 | 2.2, 2.5 | 785.4 | 785.6, 785.2 |
| CoO_4 | GL(30) | 4.4 | 4.4, 5.4 | 786.4 | 786.6, 786.2 |
| Co_1 | LA (1.2, 5.5) | 0.9 | 0.5, 1.2 | 778.0 | 778.2, 777.8 |
| Co_2 | GL(30) | 2.5 | 2.5, 3.5 | 781.0 | 781.2, 780.8 |
| Co_3 | GL(30) | 3.1 | 2.5, 3.5 | 783.0 | 783.2, 782.8 |
| Co_LMM | GL(30) | 2.4 | 0.5, 2.5 | 776.3 | 776.3, 776.1 |


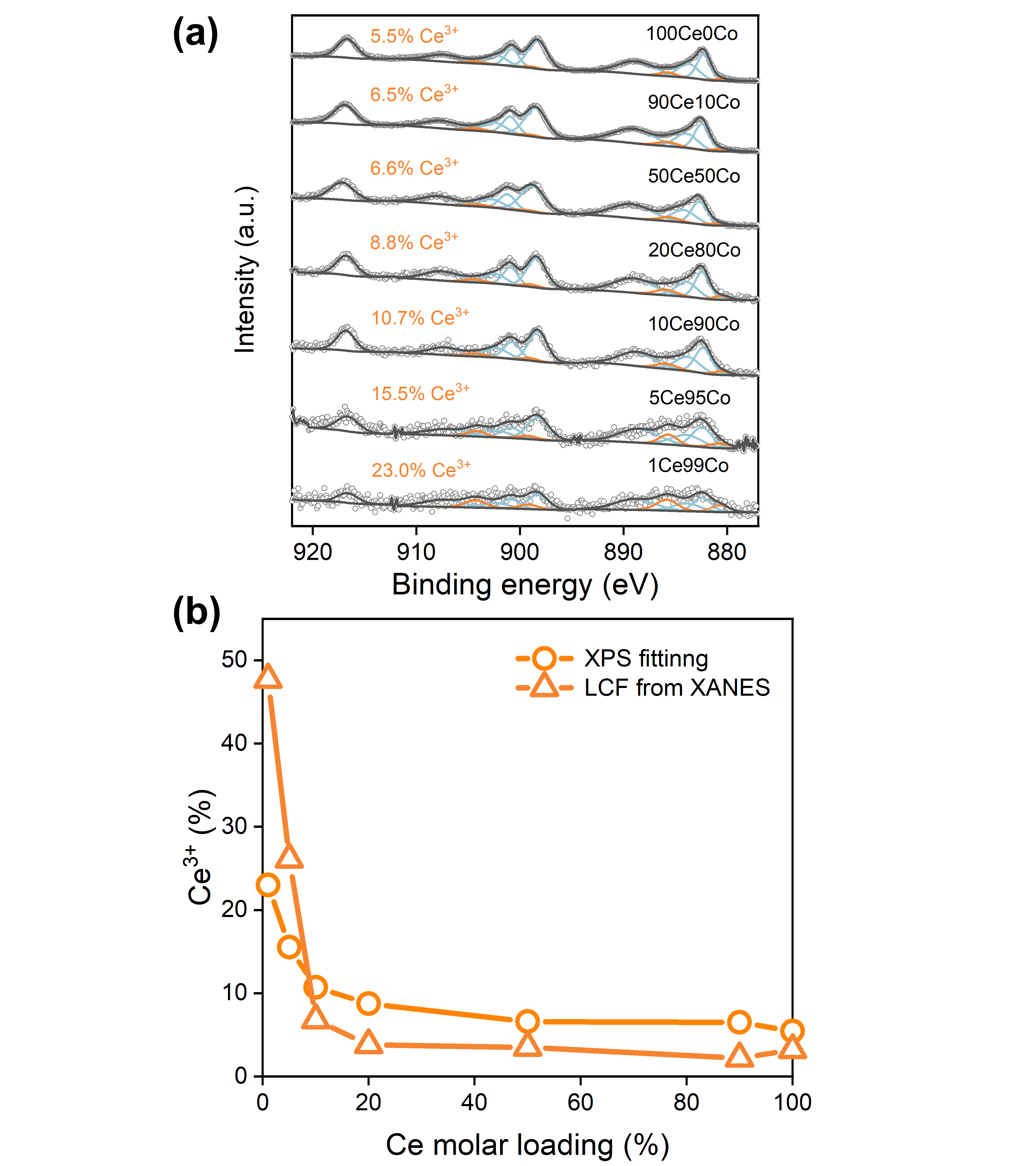


**Figure S4.** (a) Ce 3d core-line XPS spectra and (b) Ce^3+^ content determined from these XPS spectra and from XANES spectra of the as-prepared samples.


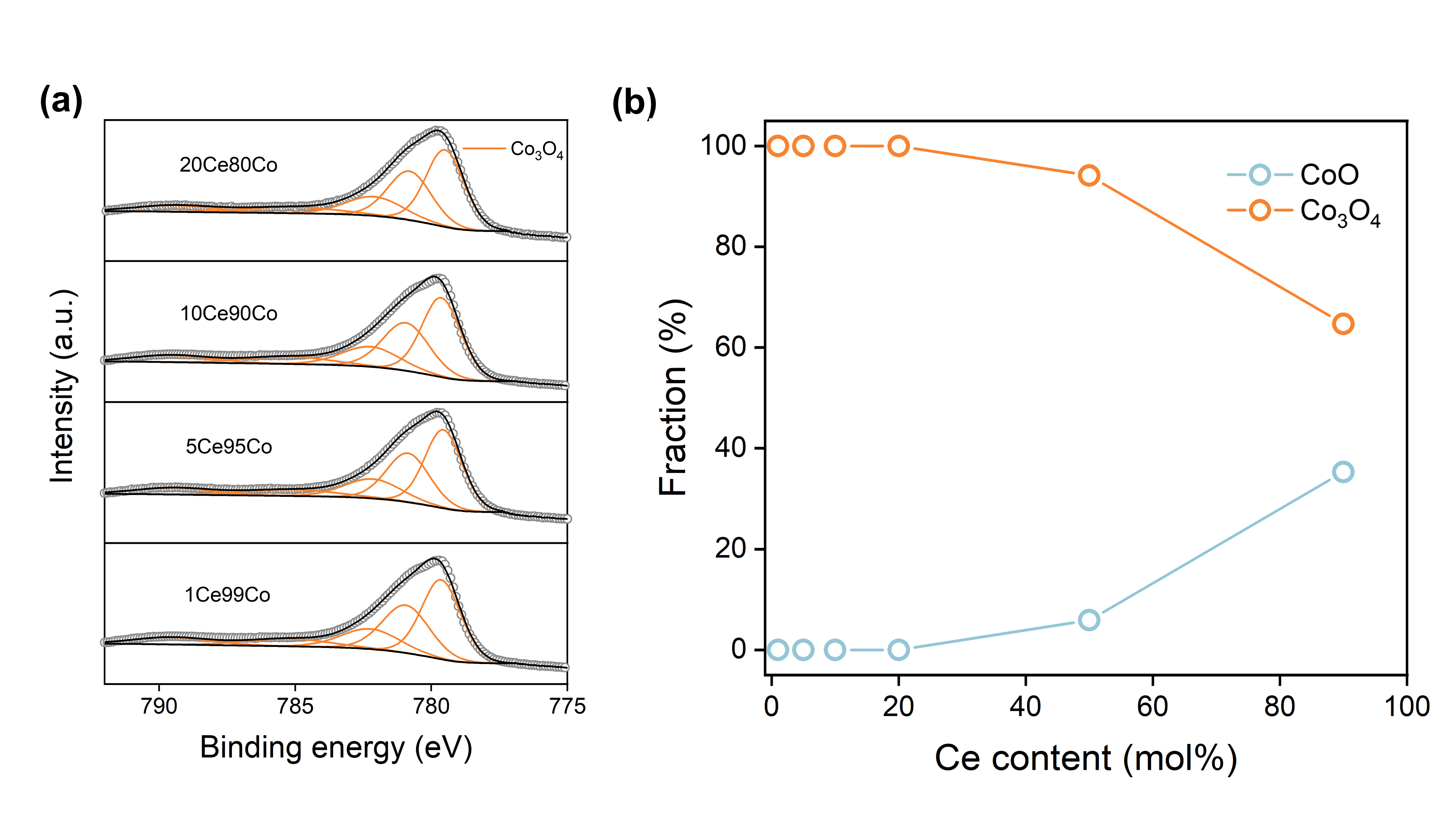


**Figure S5.** (a) Co 2p core-line XPS spectra of as-prepared samples and (b) CoO and Co_3_O_4_ fractions determined from spectra fit. The fit of 0Ce100Co, 90Ce10Co, and 50Ce50Co are shown in **Figure S3**.


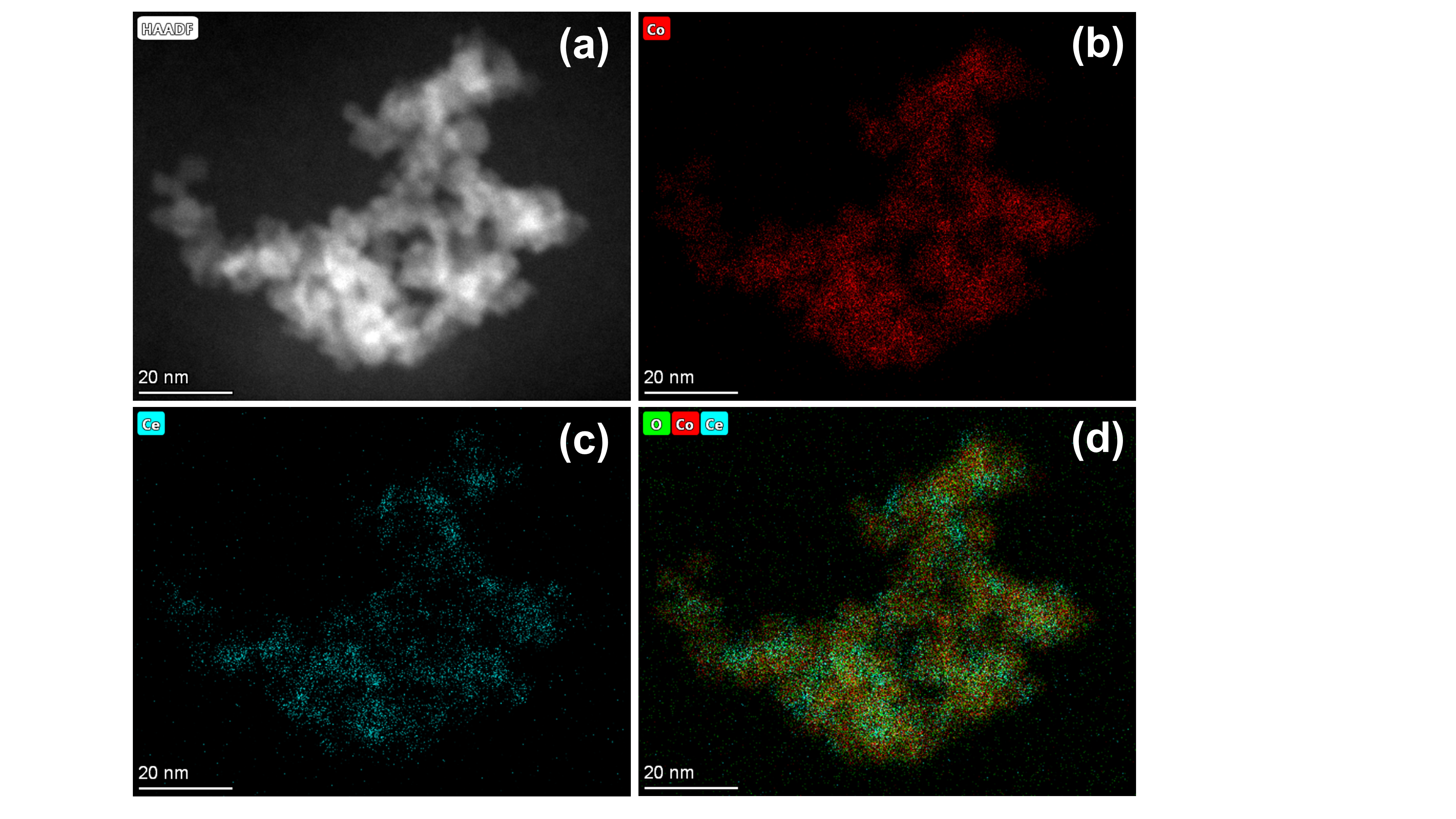


**Figure S6.** (a) HAADF-STEM image and (b, c, d) EDX maps of 10Ce90Co.


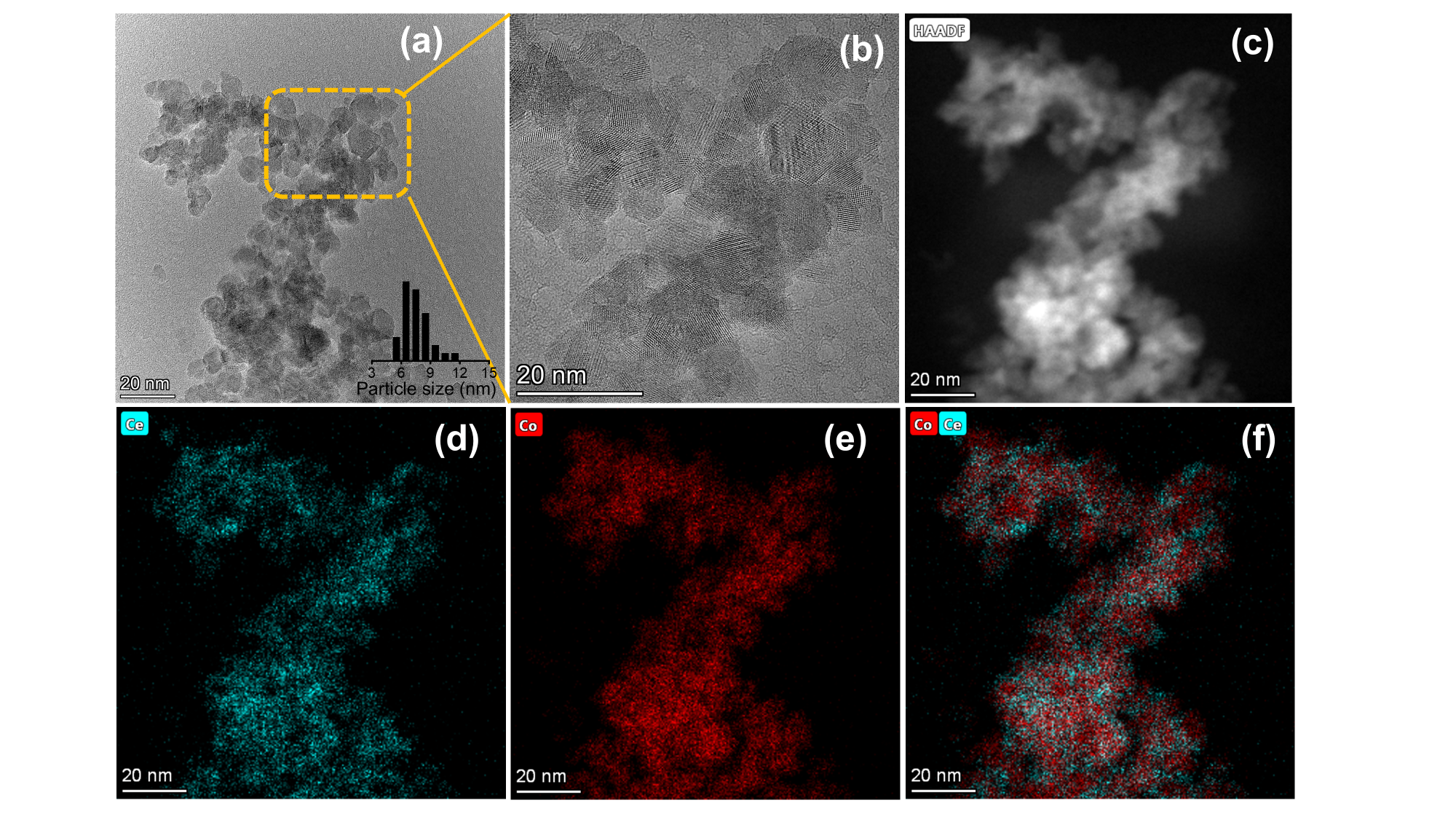


**Figure S7.** (a, b) HRTEM image (inset: particle size distribution), (c) HAADF-STEM image and (d, e, f) EDX maps of 20Ce80Co.


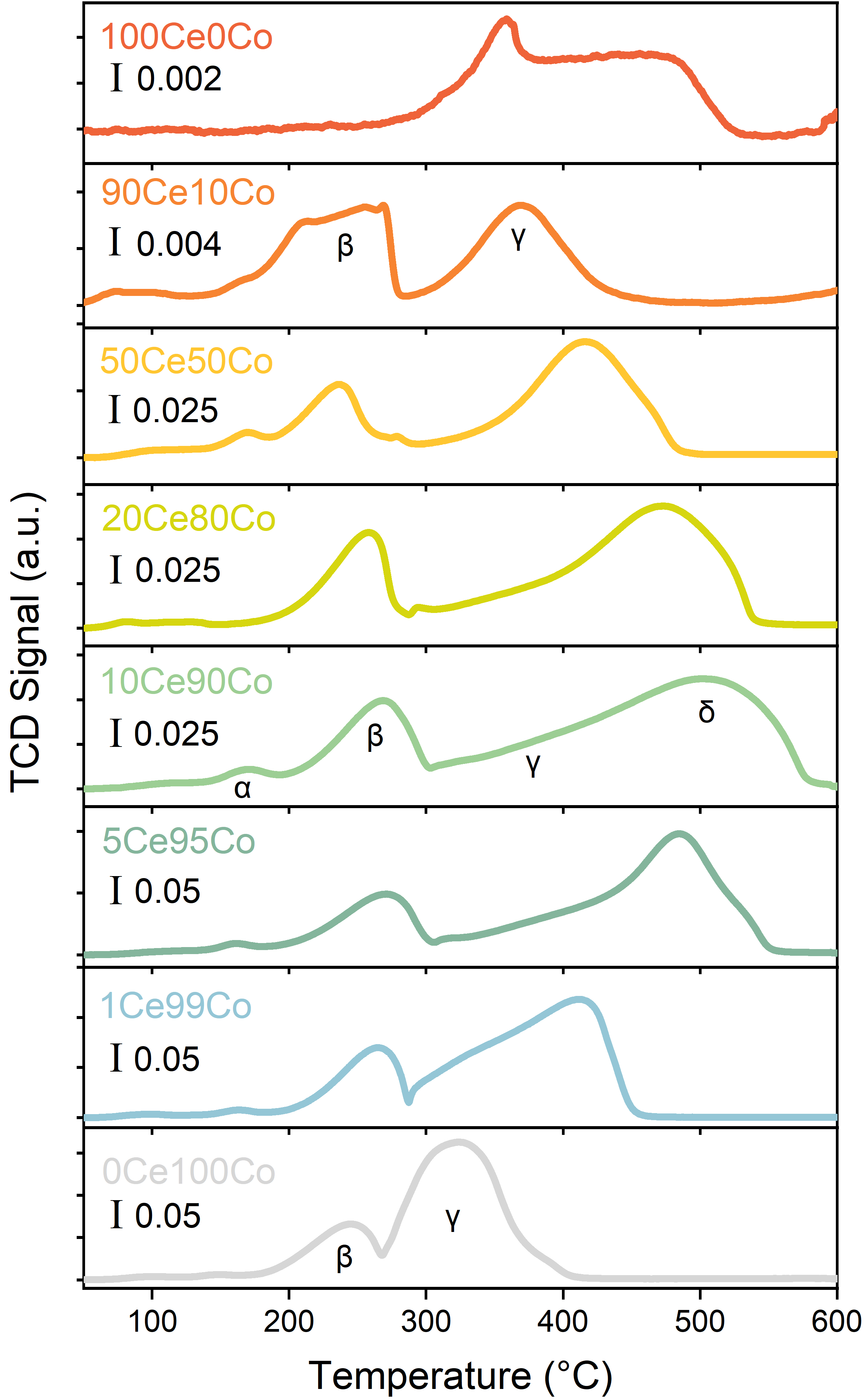


**Figure S8.** H_2_-TPR profiles of as-prepared samples.

***Note S1.***

Four features are observed in H_2_-TPR profiles: features α and β can be ascribed to surface CeO_x_ reduction and reduction from Co^3+^ to Co^2+^, respectively.^[4]^ The symmetric γ feature in 90Ce10Co and 0Ce100Co, which can be assigned to the reduction of Co^2+^ to Co^0^, shifts to a slightly higher temperature in 90Ce10Co compared to 0Ce100Co, indicating an interaction between small Co clusters and CeO_x_ support, stabilizing CoO during H_2_ reduction. The δ feature appears at much higher temperatures, implying a stronger interaction between Co nanoparticles and small CeO_x_ clusters.


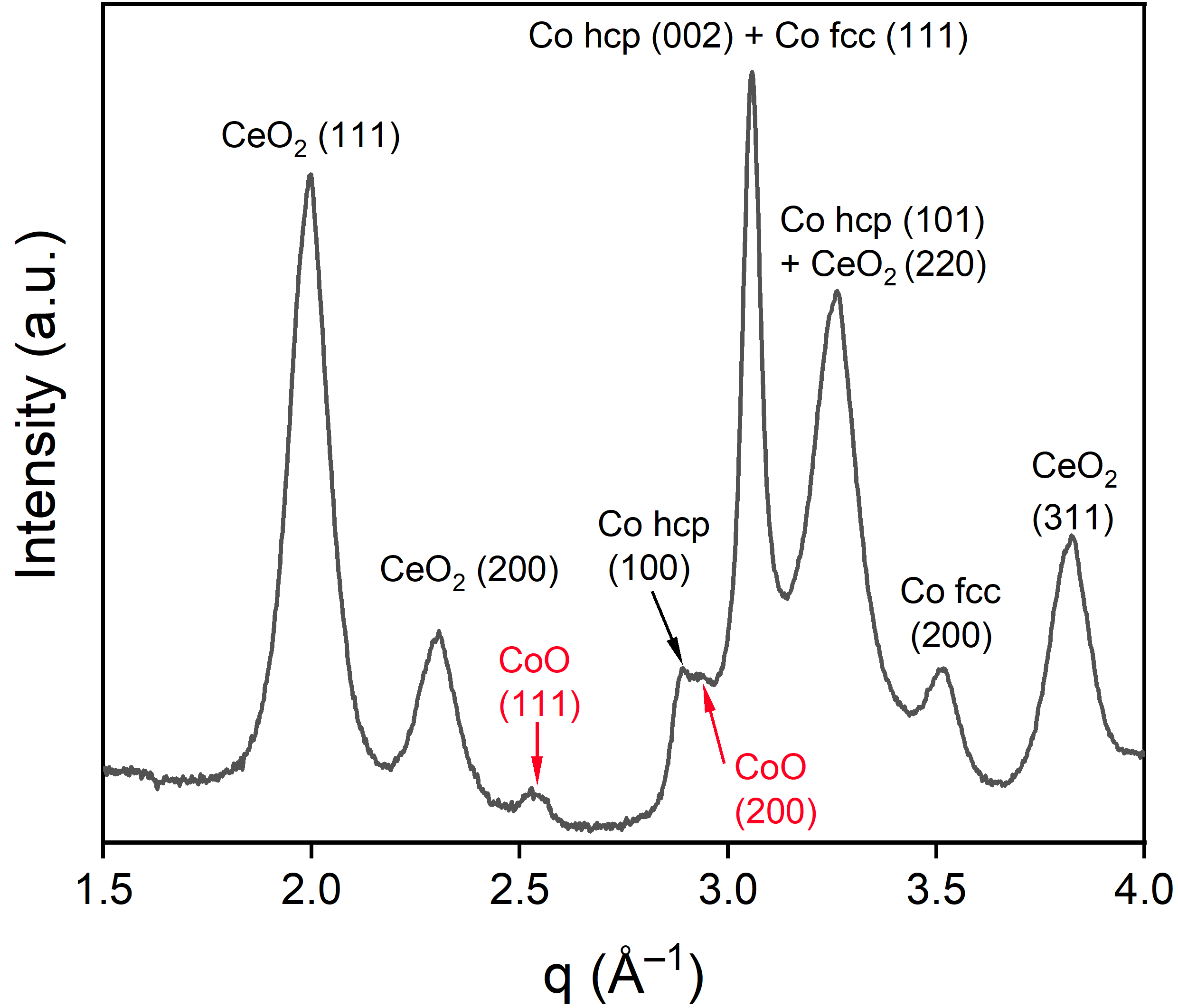


**Figure S9.** In situ XRD patterns of 20Ce80Co after reduction in H_2_ from room temperature to 500 °C followed by a dwell of 1 h.


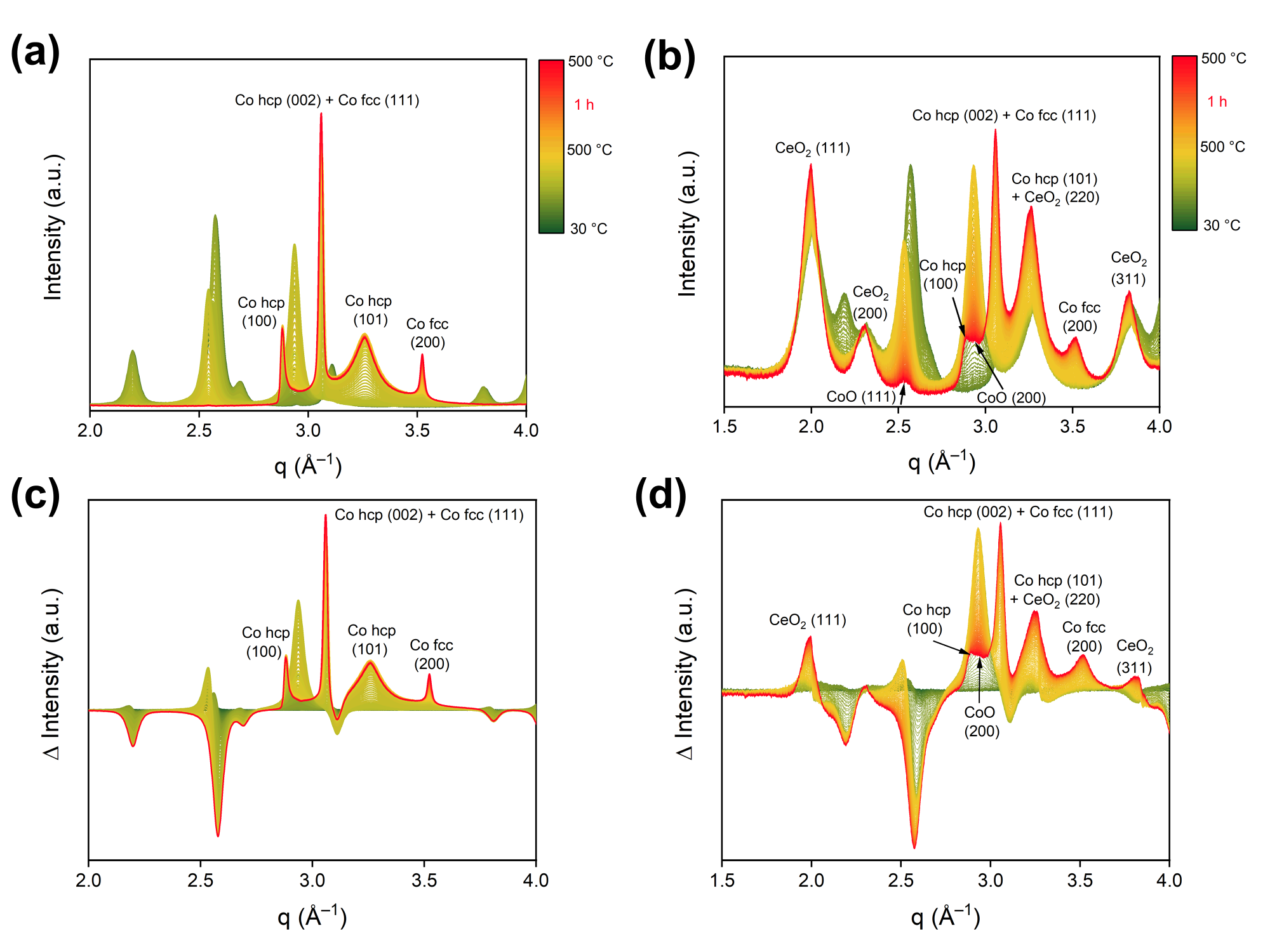


**Figure S10.** In situ XRD patterns (top) and the corresponding background-substrated patterns (bottom) of (a, c) 0Ce100Co and (b, d) 20Ce80Co during reduction in H_2_. The background-subtracted patterns were obtained by subtracting the initial pattern from subsequent ones. The temperature was increased from room temperature to 500 °C followed by a dwell of 1 h. Background-substrated patterns were used to analyze the formation rates of the hcp-Co(101) and fcc-Co(200) diffraction lines.


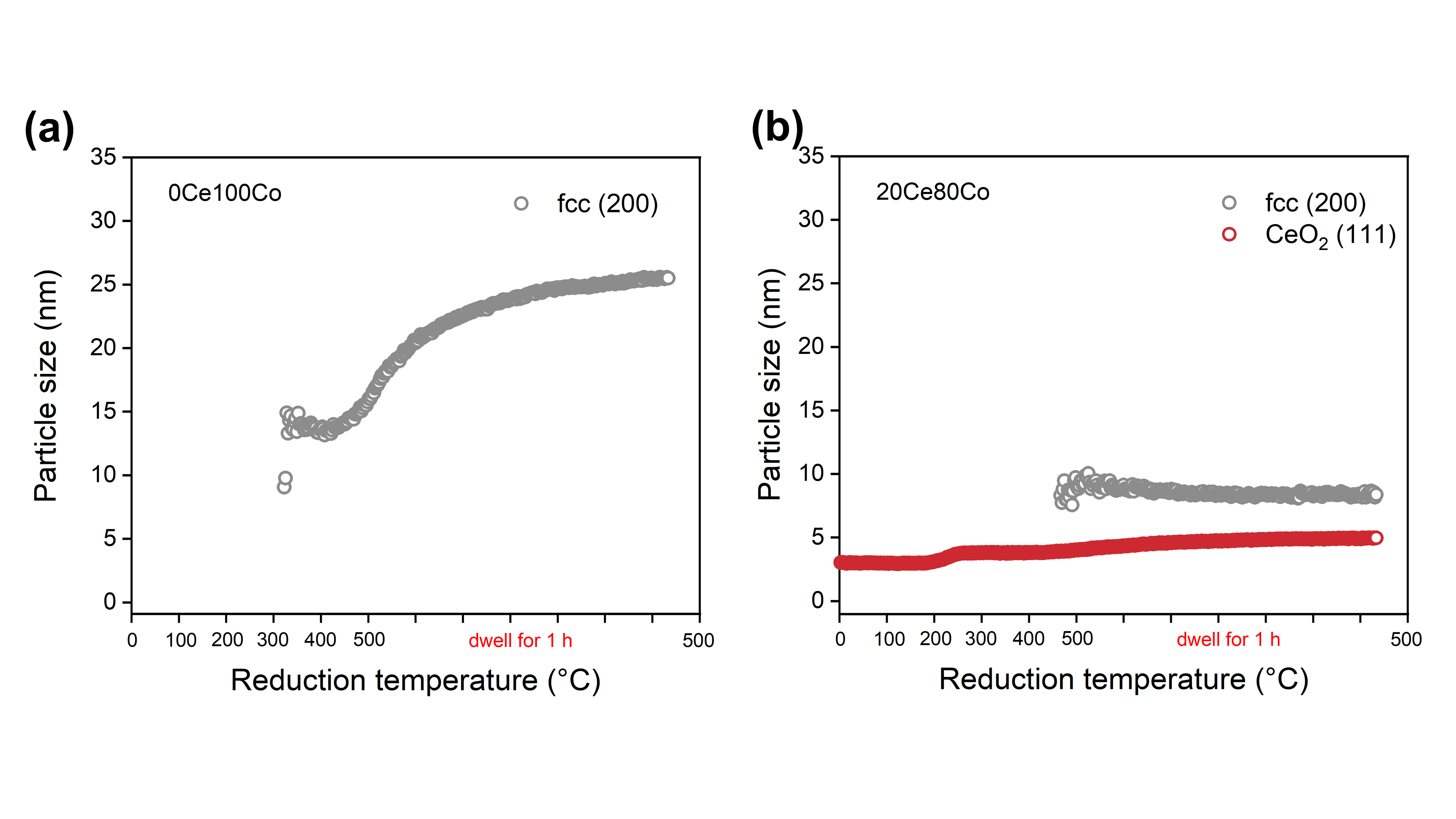


**Figure S11.** Calculated particle size for (a) 0Ce100Co and (b) 20Ce80Co during H_2_ reduction. The Scherrer equation was used to calculate the particle size of the crystallites, with the shape factor K set to 0.94. This value was chosen based on the assumption that the crystallites are spherical in shape with cubic symmetry. The hcp-Co phases were not analyzed due to the overlap between the hcp(001) and fcc(200), hcp(101) and CeO_2_ (220) diffraction lines.


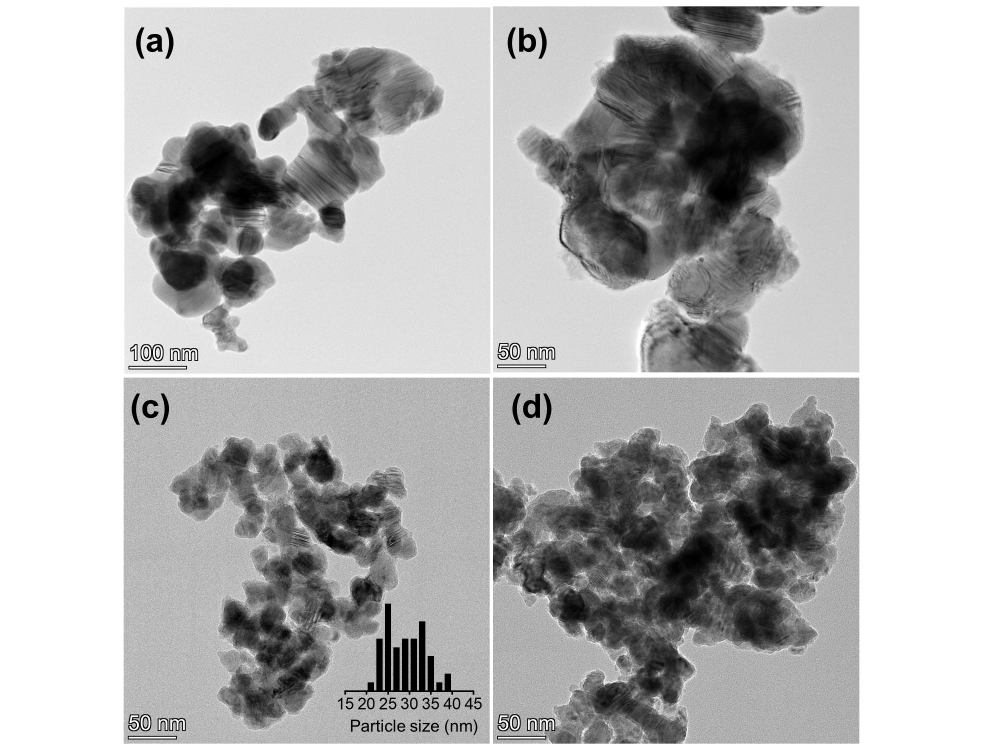


**Figure S12.** TEM images of (a, b) 0Ce100Co and (c, d) 1Ce99Co after reduction in 10 vol% H_2_ in He (50 mL/min) at 300 °C for 4 h (inset in (c) shows the particle size distribution of 1Ce99Co after reduction).


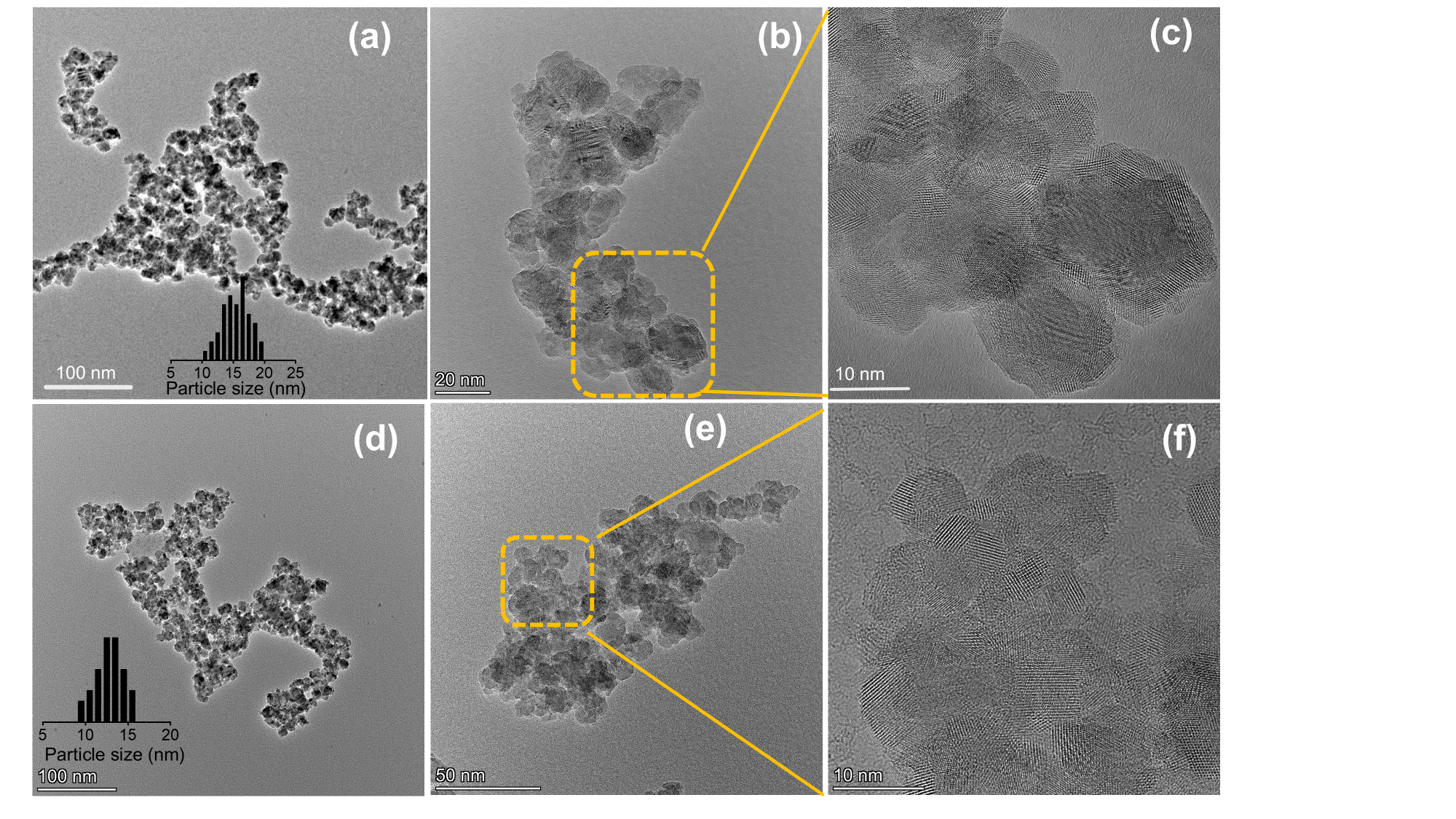


***Figure S13.*** *HRTEM images of (a, b, c) 10Ce90Co and (d, e, f) 20Ce80Co after being reduced in 10 vol% H_2_ in He (50 mL/min) at 300 °C for 4 h (insets in (a) and (d) show the particle size distributions of 10Ce90Co and 20Ce80Co, respectively).*


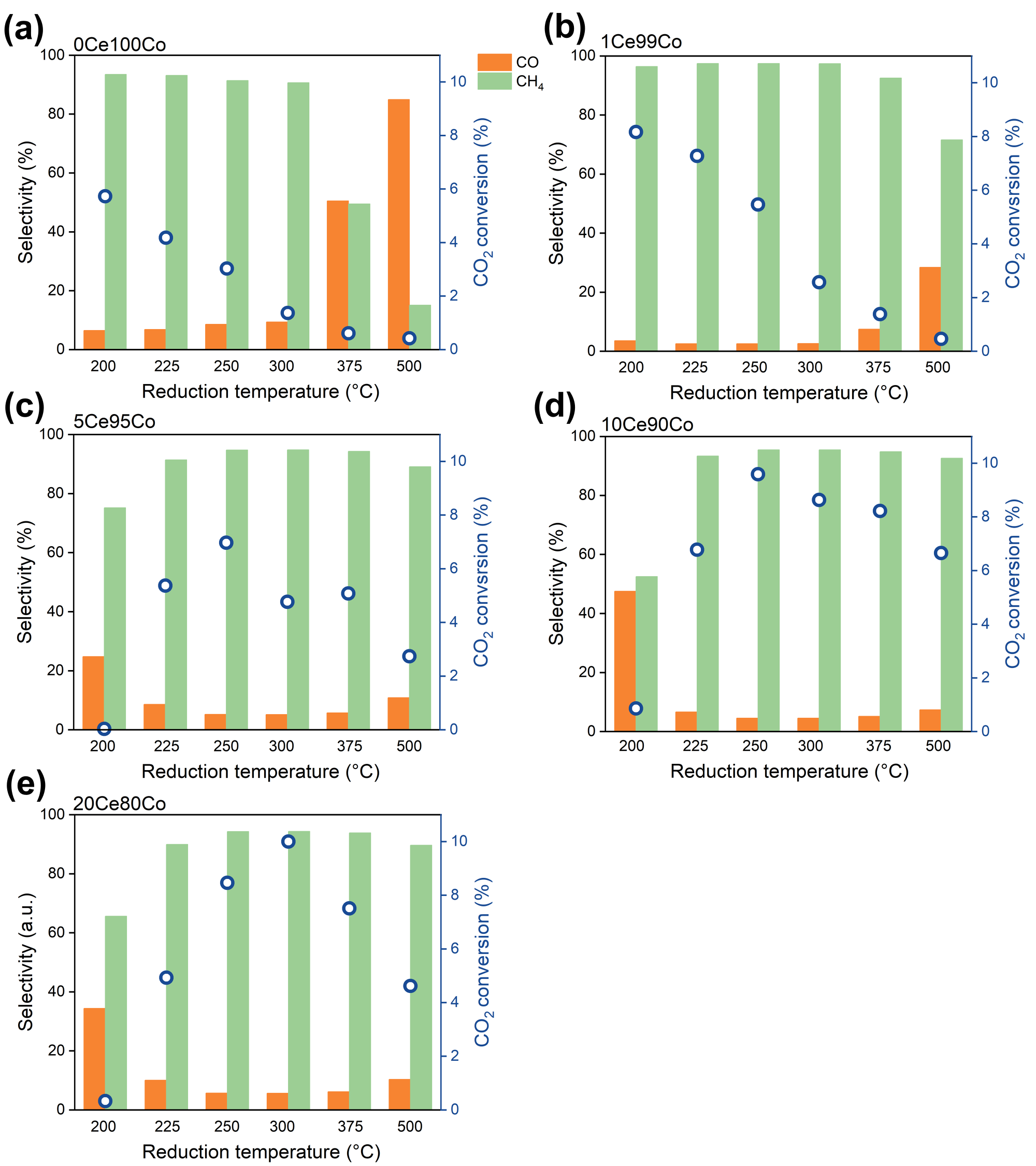


**Figure S14.** CO_2_ conversion and product selectivity over (a) 0Ce100Co; (b) 1Ce99Co; (c) 5Ce95Co; (d) 10Ce90Co and (e) 20Ce80Co after reduction at various temperatures in 10 vol% in H_2_ in He (50 mL/min) for 4 h. Reaction conditions: 200 °C, CO_2_/H_2_/He = 1/4/15, 50 mL/min. The C_2_^+^ hydrocarbons selectivity is below 1%. The CO_2_ conversion of 100Ce0Co was below the detection limit.


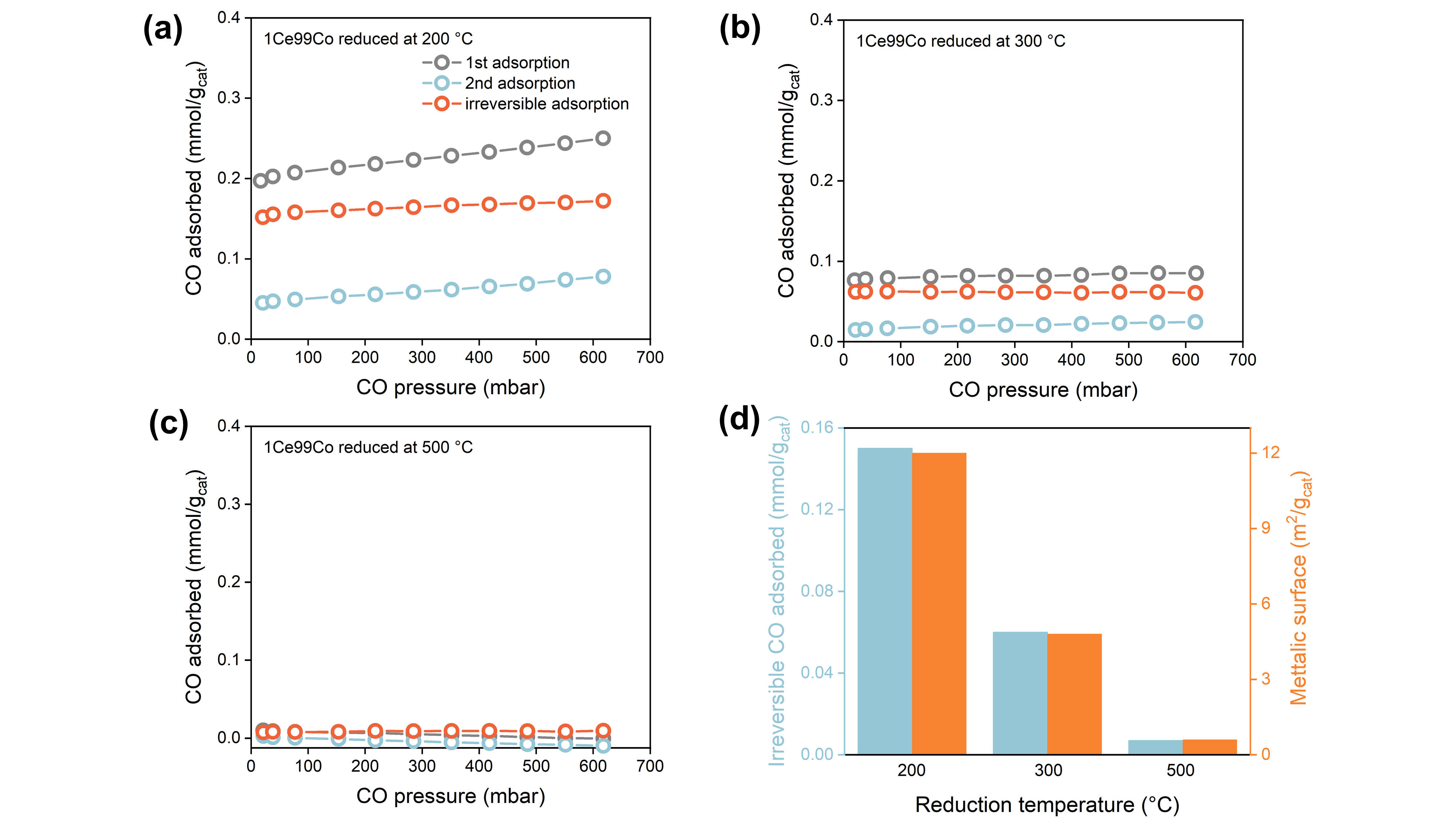


**Figure S15.** CO chemisorption results of 1Ce99Co after reduction at (a) 200 °C; (b) 300 °C; (c) 500 °C for 4 h and (d) amount of irreversible CO adsorbed and the computed metallic surface.


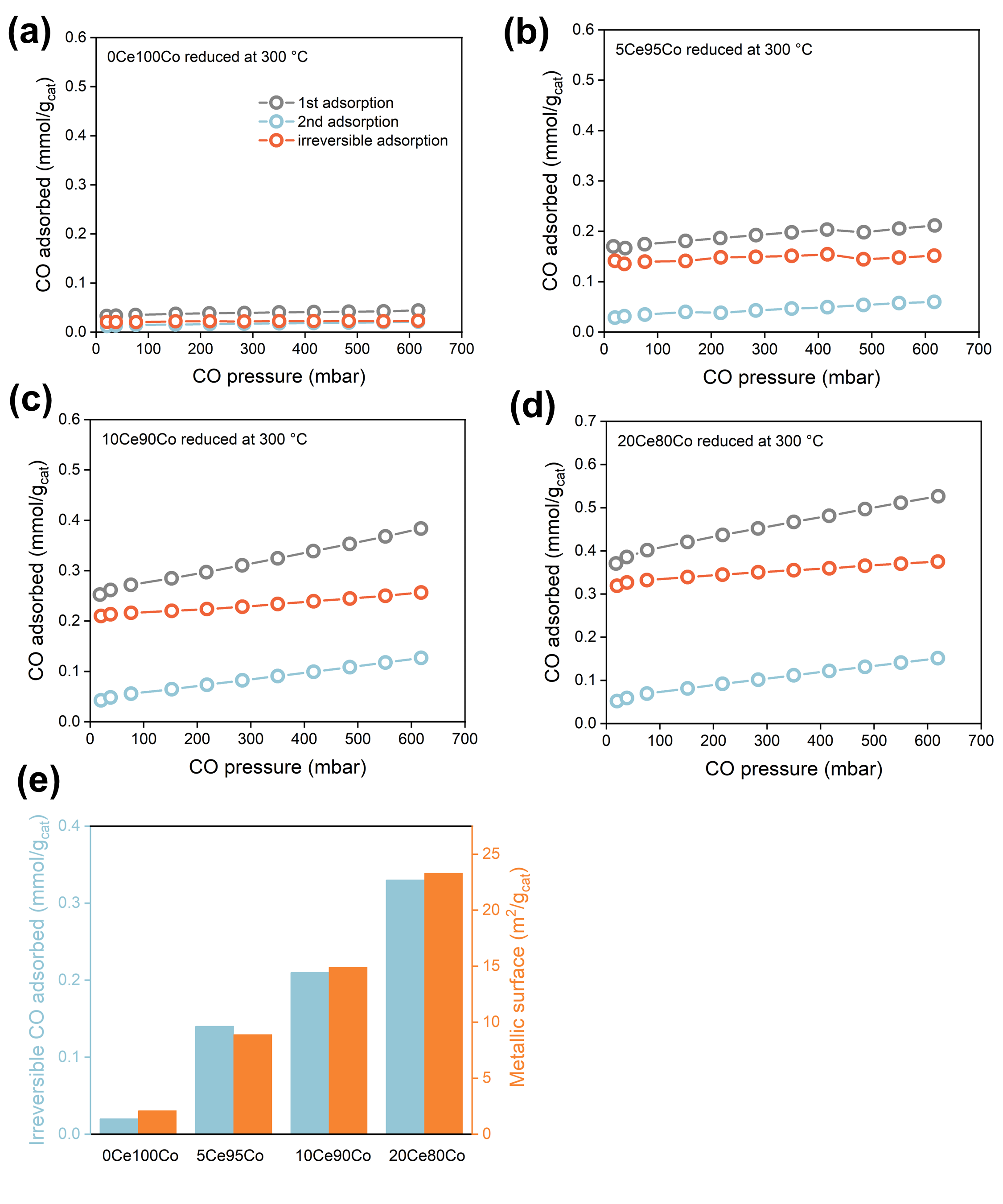


**Figure S16.** CO chemisorption results of (a) 0Ce100Co; (b) 5Ce90Co; (c) 10Ce90Co; (d) 20Ce80Co after reduction at 300 °C for 4 h and (e) amount of irreversibly adsorbed CO and the metallic surface (results for 1Ce99Co in **Figure S15**).


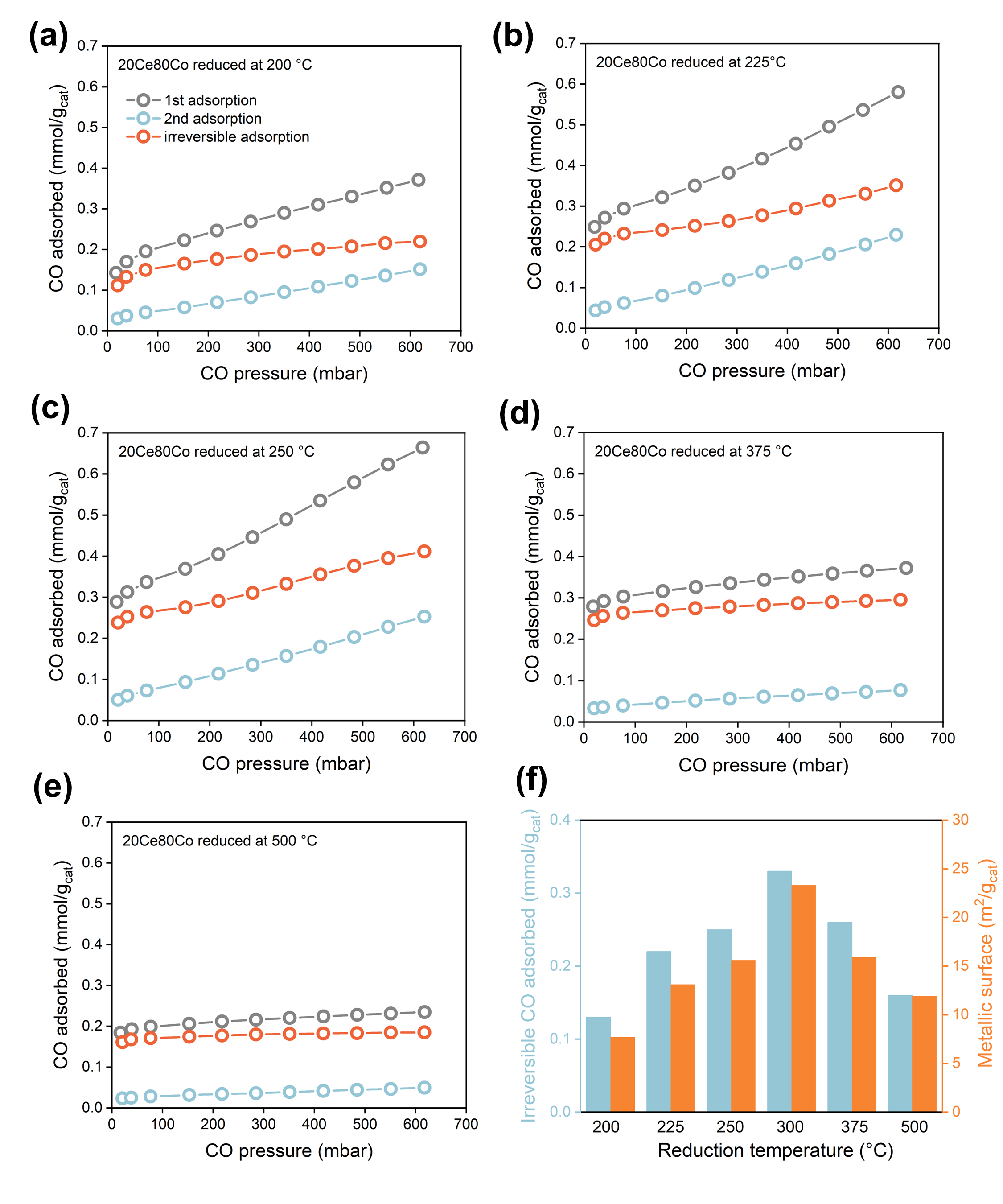


**Figure S17**. CO chemisorption results of 20Ce80Co after reduction at (a) 200 °C; (b) 225 °C; (c) 250 °C; (d) 375 °C; (e) 500 °C for 4 h and (f) amount of irreversibly adsorbed CO and the metallic surface (results for 20Ce80Co reduced at 300 °C in **Figure S16**).


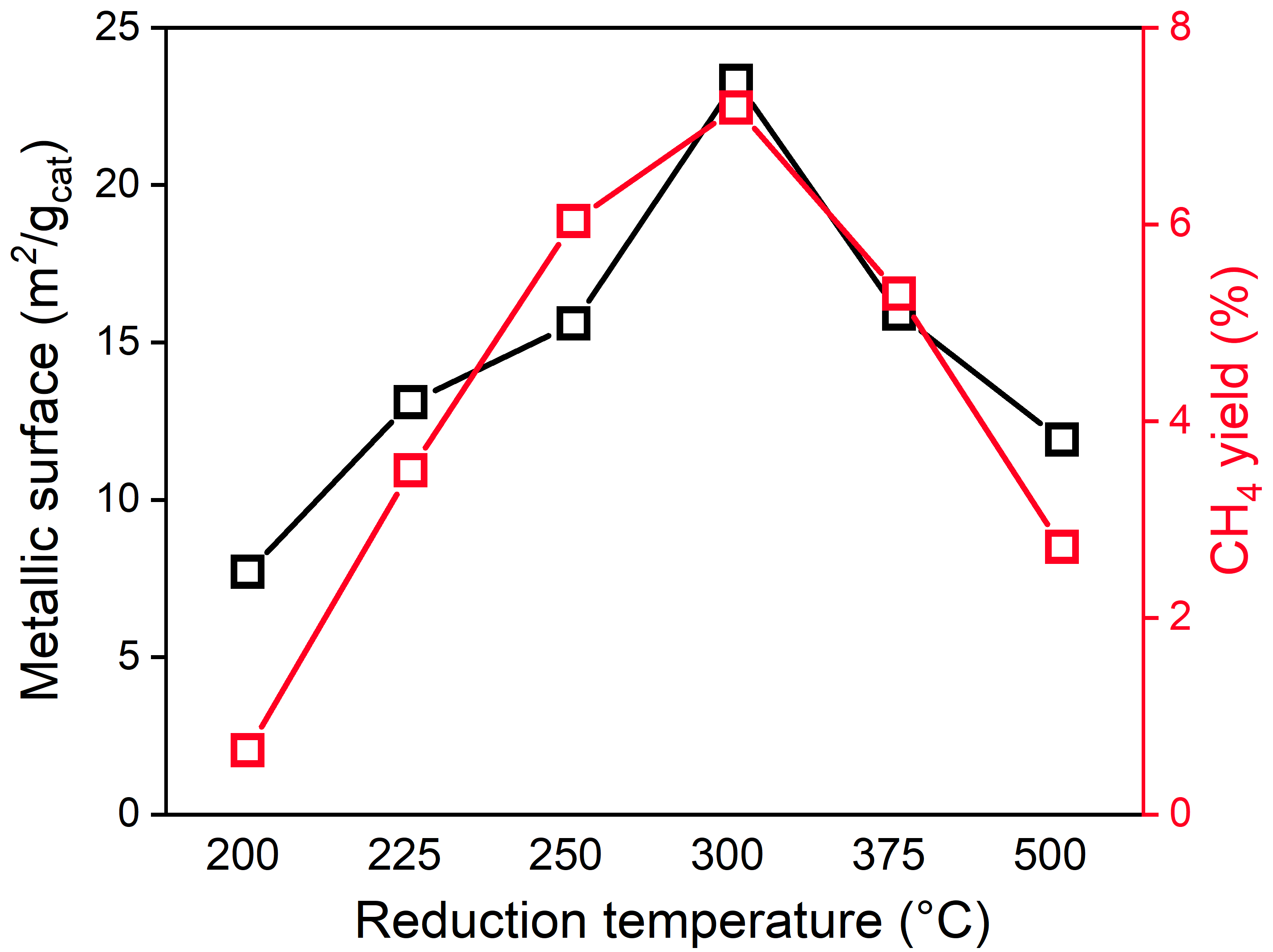


**Figure S18.** Correlation between the metallic surface and the CH_4_ yield for 20Ce80Co reduced at various temperatures.

***Table S4****. CO uptake and specific surface area for inverse CeO_x_/Co samples.*

| Samples | CO adsorbed (mmol/g) | BET surface area (m^2^/g) | CO adsorbed  (μmol/m^2^) |
| --- | --- | --- | --- |
| 0Ce100Co  reduced at 300 °C | 0.02 | 17.70 | 1.13 |
| 1Ce99Co  reduced at 200 °C | 0.15 | – | – |
| 1Ce99Co  reduced at 300 °C | 0.06 | 73.40 | 1.09 |
| 1Ce99Co  reduced at 500 °C | 0.01 | – | – |
| 5Ce95Co  reduced at 300 °C | 0.14 | 76.40 | 1.96 |
| 10Ce90Co  reduced at 300 °C | 0.22 | 82.20 | 2.80 |
| 20Ce80Co  reduced at 200 °C | 0.13 | – | – |
| 20Ce80Co  reduced at 225 °C | 0.22 | – | – |
| 20Ce80Co  reduced at 250 °C | 0.26 | – | – |
| 20Ce80Co  reduced at 300 °C | 0.33 | 85.30 | 4.10 |
| 20Ce80Co  reduced at 375 °C | 0.26 | – | – |
| 20Ce80Co  reduced at 500 °C | 0.17 | – | – |


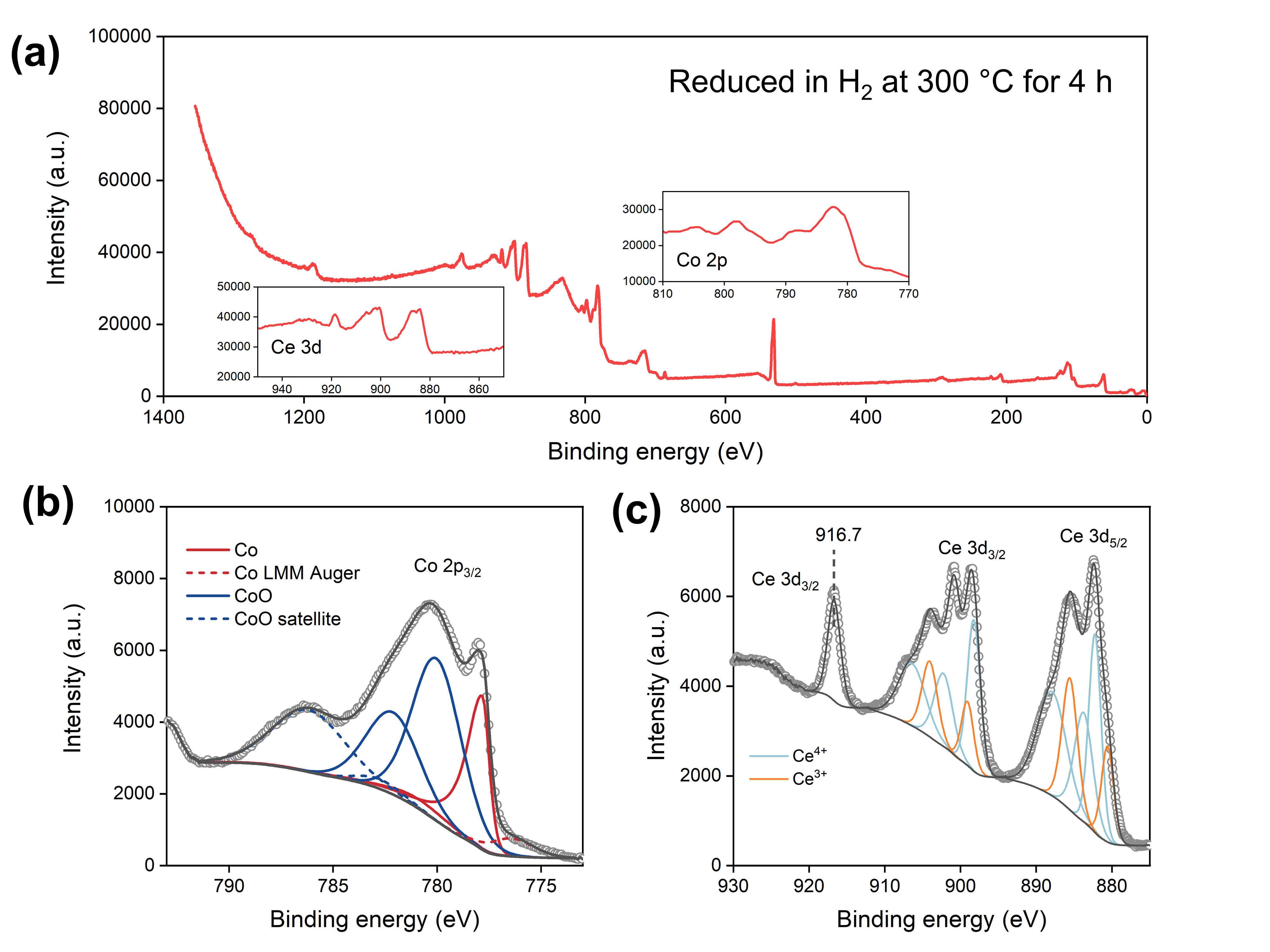


**Figure S19.** Quasi-in situ XPS: (a) survey scan; (b) Co 2p region and (c) Ce 3d region of 20Ce80Co after reduction at 300 °C for 4 h. The XPS fitting models are shown in **Figure S3**.


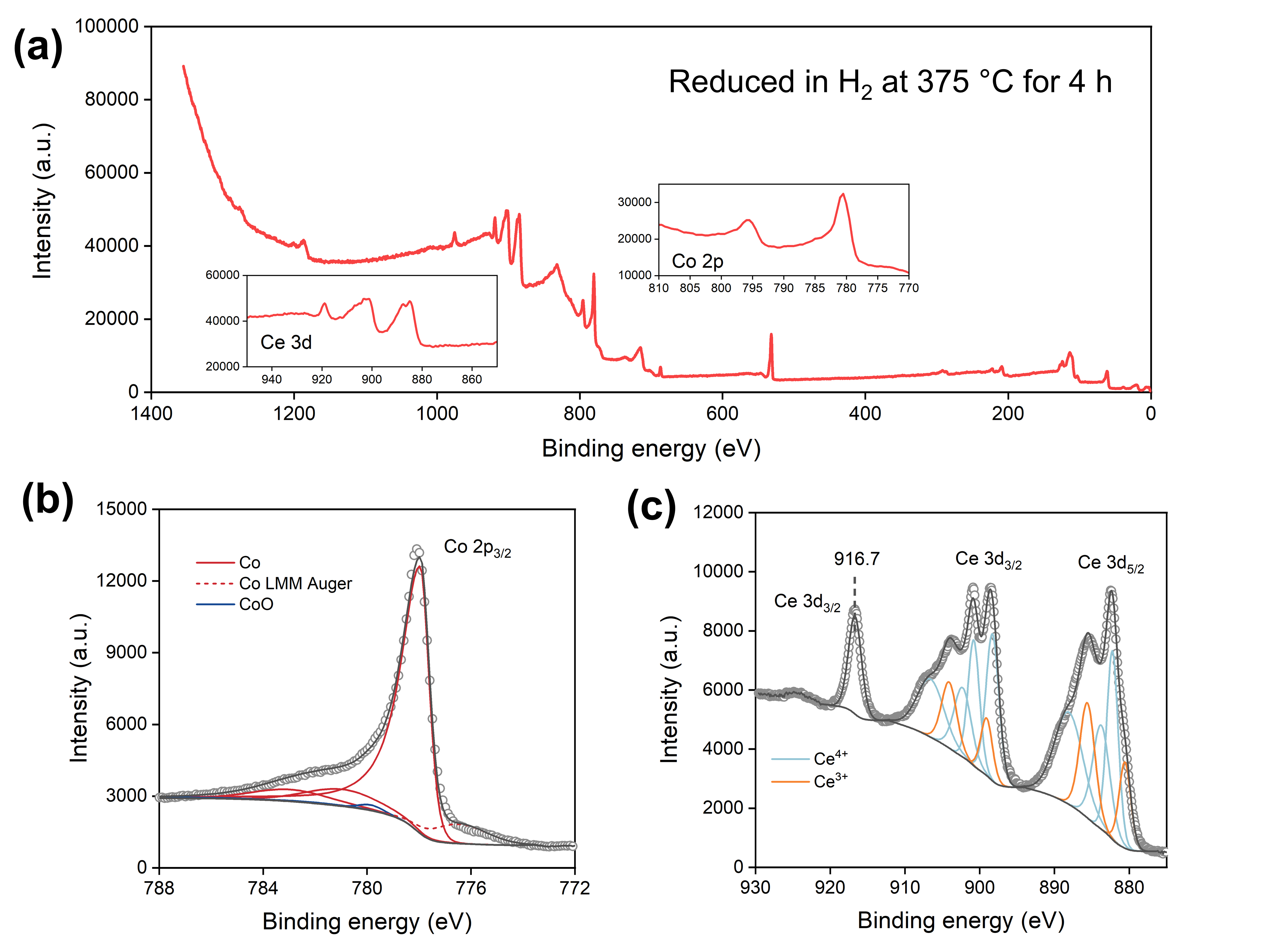


**Figure S20.** Quasi-in situ XPS: (a) survey scan; (b) Co 2p region and (c) Ce 3d region of 20Ce80Co after reduction at 375 °C for 4 h. The XPS fitting models are shown in **Figure S3**.


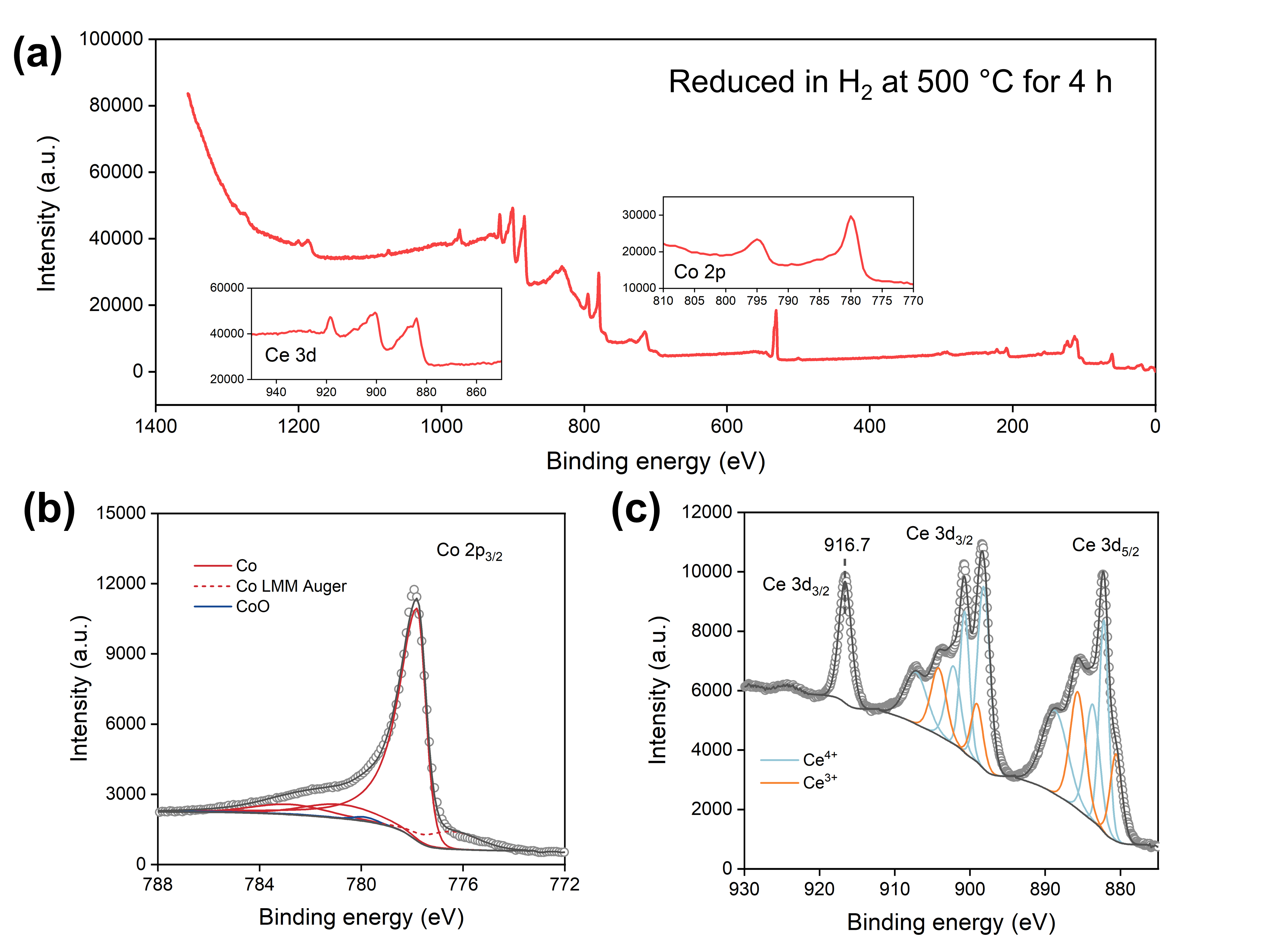


**Figure S21.** Quasi-in situ XPS: (a) survey scan; (b) Co 2p region and (c) Ce 3d region of 20Ce80Co after reduction at 300 °C for 4 h. The XPS fitting models are shown in **Figure S3**.

| Reduction conditions | Surface Ce/Co (%) | Metallic Co (%) | Ce^3+^ (%) |
| --- | --- | --- | --- |
| 300 °C for 4 h | 0.57 | 28.4 | 25.0 |
| 375 °C for 4 h | 1.05 | 97.6 | 24.0 |
| 500 °C for 4 h | 1.10 | 97.7 | 25.4 |

**Table S5**. Quasi-in situ XPS results of the 20Ce80Co sample after H_2_ reduction.

Surface Ce/Co ratios calculated using the areas of Co 2p_3/2_ region and Ce 3d_5/2_ region with relative sensitivity factors of 2.393 and 5.282, respectively. Co LMM Auger peak and CoO satellite peak were excluded for the calculation.


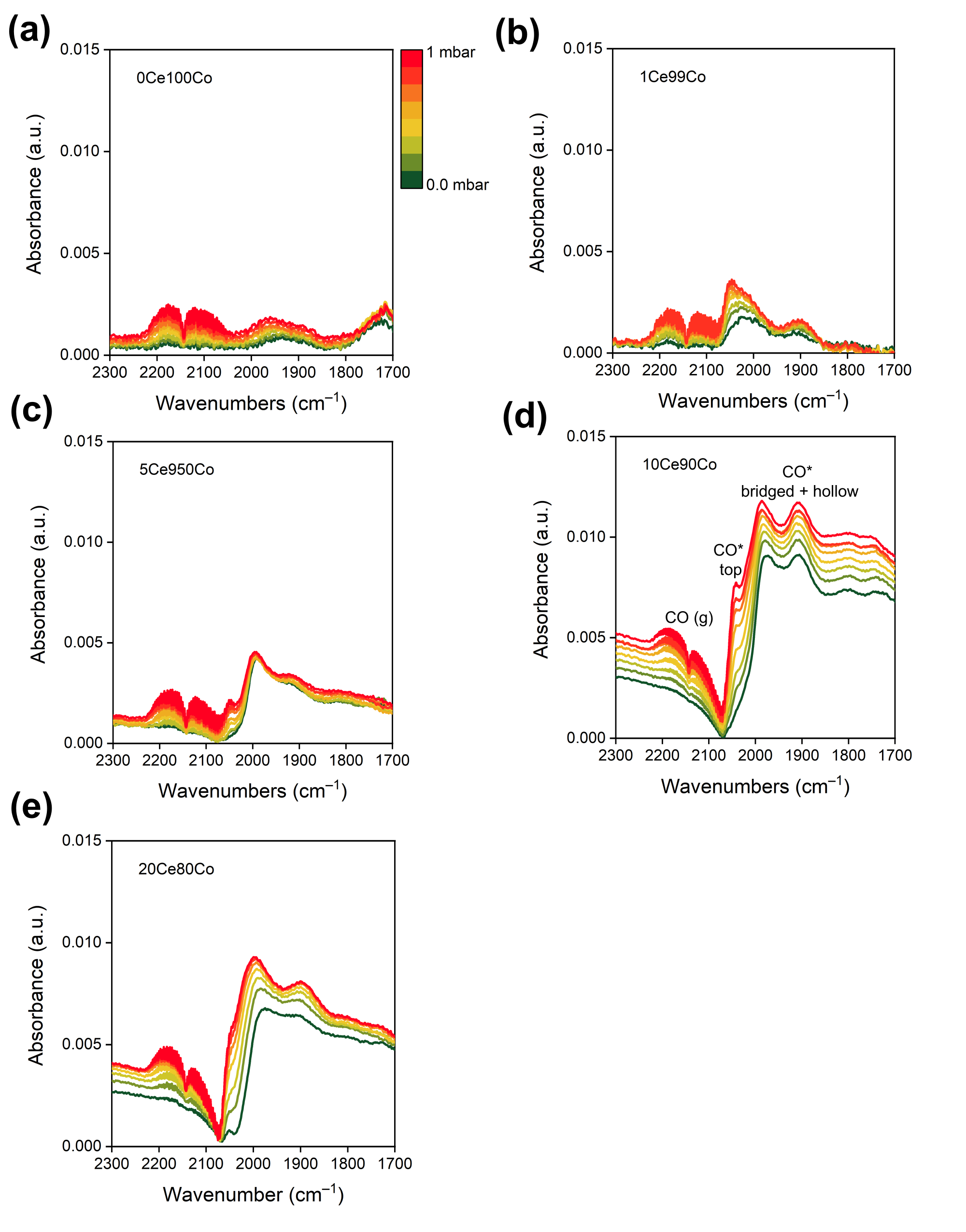


***Figure S22***. *CO IR spectra of (a) 0Ce100Co; (b) 1Ce99Co; (c) 5Ce95Co; (d) 10Ce90Co and (e) 20Ce80Co. Samples were reduced in H_2_ at 300 °C for 4 h; the measurements were conducted at 50 °C with CO pressure increasing from 0 to 1 mbar (blue to red).*


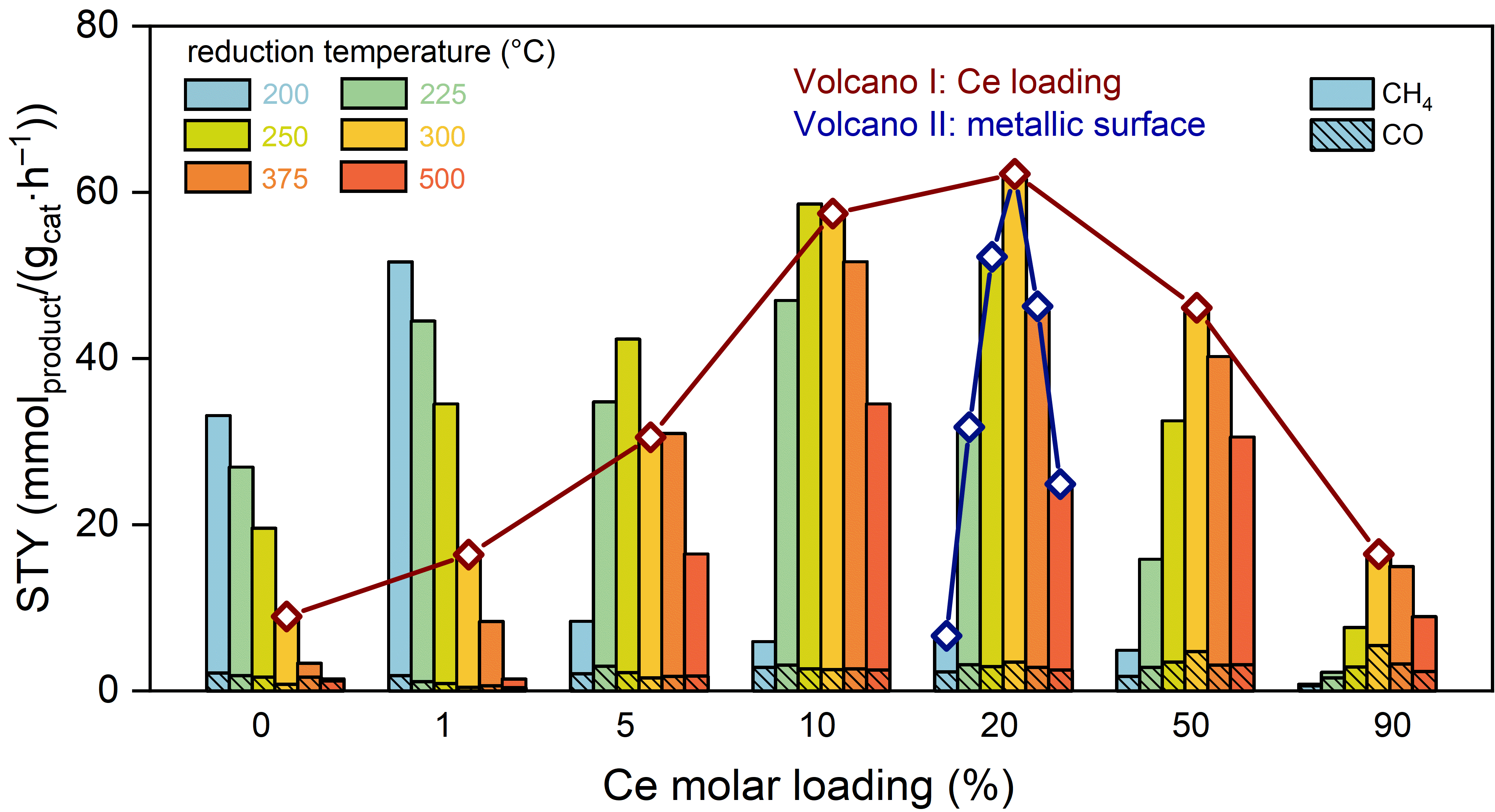


**Figure S23.** Space-time yield of the products of CO_2_ hydrogenation. Reaction conditions: 200 °C, CO_2_/H_2_/He = 1/4/15, 50 mL/min. The C_2_^+^ hydrocarbons selectivity was below 1%. The CO_2_ conversion of 100Ce0Co was below the detection limit.

**Table S6.** Catalytic performance of Co-containing catalysts in CO_2_ methanation from literature.

| Catalysts | Reaction conditions | CH_4_ STY  (mmol/(g_cat_·h)) | References |
| --- | --- | --- | --- |
| 20Ce80Co | 10 mg, CO_2_/H_2_ = 2.5/10 mL/min, 200 °C, 1 bar | 58.3 | This work |
| Ni/CeO_2_-Co_3_O_4_ | 500 mg, CO_2_/H_2_ = 6/24 mL/min, 200 °C, 1 bar | 13.7 | ^[5]^ |
| CoZn-Z | 40 mg, CO_2_/H_2_ = 5/20 mL/min,  225 °C, 1 bar | 15.1 | ^[6]^ |
| Ni/3DDP-Co_3_O_4_ | 50 mg, CO_2_/H_2_ = 2/8 mL/min,  200 °C, 1 bar | 22.5 | ^[7]^ |
| m-Co_3_O_4_ | 200 mg, CO_2_/H_2_ = 10/40 mL/min,  310 °C, 1 bar | 61.2 | ^[8]^ |
| Pt/m-Co_3_O_4_ | 200 mg, CO_2_/H_2_ = 10/40 mL/min,  310 °C, 1 bar | 75.6 | ^[8]^ |
| Co_8_/ZnO | 500 mg, CO_2_/H_2_ = 20/80 mL/min,  200 °C, 5 bar | 7.5 | ^[9]^ |
| Cu_0.4_Co_2.6_ | 200 mg, CO_2_/H_2_ = 10/40 mL/min,  250 °C, 1 bar | 16.2 | ^[10]^ |
| Mn_0.1_Co_2.9_O_4_ | 150 mg, CO_2_/H_2_ = 10/40 mL/min,  300 °C, 1 bar | 10.8 | ^[11]^ |
| (Co_0.95_Ru_0.05_)_3_O_4_ | 100 mg, CO_2_/H_2_ = 10/40 mL/min,  220 °C, 1 bar | 6.2 | ^[12]^ |
| Co-S | 50 mg, CO_2_/H_2_ = 5/20 mL/min,  260 °C, 1 bar | 18 | ^[13]^ |
| Zr-Co_3_O_4_ | 100 mg, CO_2_/H_2_ = 6/24 mL/min,  200 °C, 5 bar | 88.4 | ^[14]^ |
| Ce-Co_3_O_4_ | 100 mg, CO_2_/H_2_ = 6/24 mL/min,  200 °C, 5 bar | 40.2 | ^[14]^ |
| 0.2Pt1Co/BaZrO_3_ | 200 mg, CO_2_/H_2_ = 3/9 mL/min,  250 °C, 1 bar | 1.44 | ^[15]^ |
| CoCZ700 | 50 mg, CO_2_/H_2_ = 2.5/10 mL/min, 225 °C, 1 bar | 18.4 | ^[4]^ |
| Co/C(L) | 100 mg, CO_2_/H_2_ = 2/8 mL/min, 200 °C, 1 bar | 2.4 | ^[16]^ |
| 0.5Co-NiSil | 100 mg, CO_2_/H_2_ = 6/24 mL/min, 250 °C, 1 bar | 74.8 | ^[17]^ |
| Co/CZ-MOF | 100 mg, CO_2_/H_2_ = 6.25/18.75 mL/min, 220 °C, 15 bar | 34.8 | ^[18]^ |
| Co(93)Fe(7) | 1 g, CO_2_/H_2_ = 2/46 mL/min,  250 °C, 1 bar | 3.6 | ^[19]^ |
| Co/2%Y-TiO_2_ | 100 mg, CO_2_/H_2_ = 1.2/4.8 mL/min, 200 °C, 5 bar | 3.2 | ^[20]^ |
| Co/KIT-6 | 100 mg, CO_2_/H_2_ = 3.7/17 mL/min, 200 °C, 1 bar | 14.9 | ^[21]^ |
| CoNT-1 | 100 mg, CO_2_/H_2_ = 2/8 mL/min, 200 °C, 1 bar | 2.7 | ^[22]^ |
| CoNi | 100 mg, CO_2_/H_2_ = 5/20 mL/min, 200 °C, 1 bar | 6.0 | ^[23]^ |
| Ni-Co-MgO | 150 mg, CO_2_/H_2_ = 10/40 mL/min, 200 °C, 1 bar | 12.1 | ^[24]^ |
| CoPt0.43-Al_2_O_3_ | 50 mg, CO_2_/H_2_ = 6/24 mL/min, 200 °C, 1 bar | 14.5 | ^[25]^ |
| Co-10H_2_/Ar | 120 mg, CO_2_/H_2_ = 18/72 mL/min, 200 °C, 1 bar | 14.9 | ^[26]^ |
| Co/CeO_2_(CCI) | 100 mg, CO_2_/H_2_ = 4/16 mL/min, 200 °C, 1 bar | 5.4 | ^[27]^ |
| CoCe09 | 50 mg, CO_2_/H_2_ = 5/20 mL/min, 240 °C, 1 bar | 38.2 | ^[28]^ |
| CK120(Co/SiO_2_) | 50 mg, CO_2_/H_2_ = 5/20 mL/min, 240 °C, 1 bar | 14.4 | ^[29]^ |
| 25%Co/KIT-6 | 50 mg, CO_2_/H_2_ = 5/20 mL/min, 240 °C, 1 bar | 43.2 | ^[30]^ |
| 12Ni3Co/M-Ce80Zr20 | 50 mg, CO_2_/H_2_ = 4/16 mL/min, 200 °C, 1 bar | 5.4 | ^[31]^ |
| Ni-Co/Al_2_O_3_-ZrO_2_ | 500 mg, CO_2_/H_2_ = 10/40 mL/min, 200 °C, 1 bar | 6.7 | ^[32]^ |
| NiCo-HT | 50 mg, CO_2_/H_2_ = 5/20 mL/min, 240 °C, 1 bar | 18 | ^[33]^ |
| Co/CeO_2_ | 60 mg, CO_2_/H_2_ = 7.5/67.5 mL/min, 210 °C, 1 bar | 7.9 | ^[34]^ |


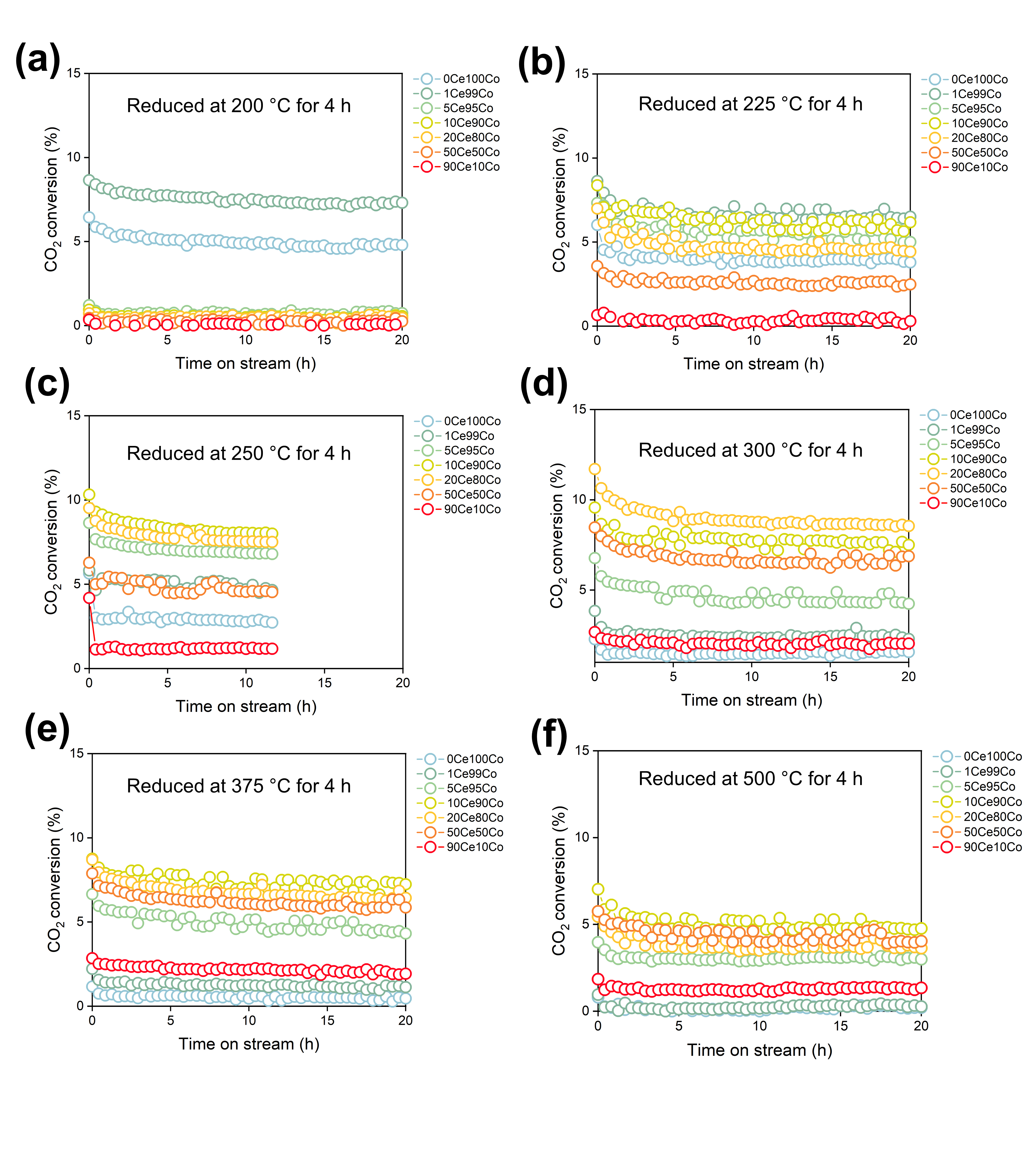


**Figure S24.** Stability test of catalysts after reduction in 10 vol% H_2_ in He (50 mL/min) at (a) 200 °C; (b) 225 °C; (c) 250 °C; (d) 300 °C; (e) 375 °C and (f) 500 °C for 4 h. Reaction conditions: 200 °C, CO_2_/H_2_/He = 1/4/15, 50 mL/min.


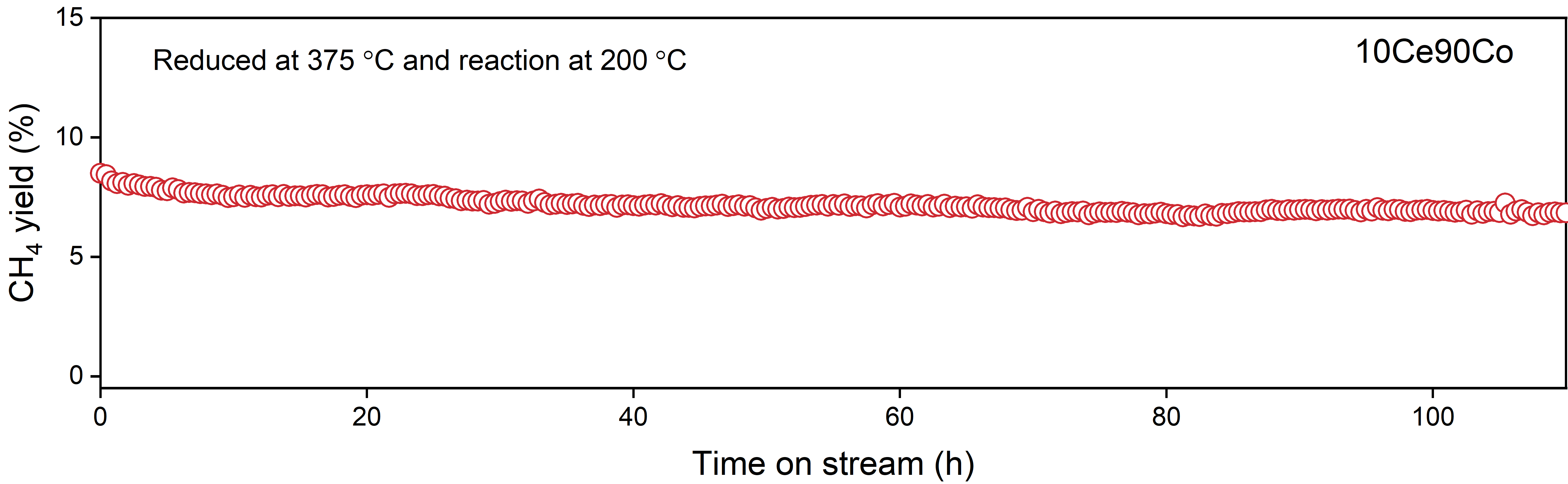


**Figure S25**. CO_2_ hydrogenation performance of 10Ce90Co during a 110-hour stability test. The sample was reduced in 10 vol% H_2_ in He (50 mL/min) at 375 °C for 4 h. Reaction conditions: 200 °C, CO_2_/H_2_/He = 1/4/15, 50 mL/min.


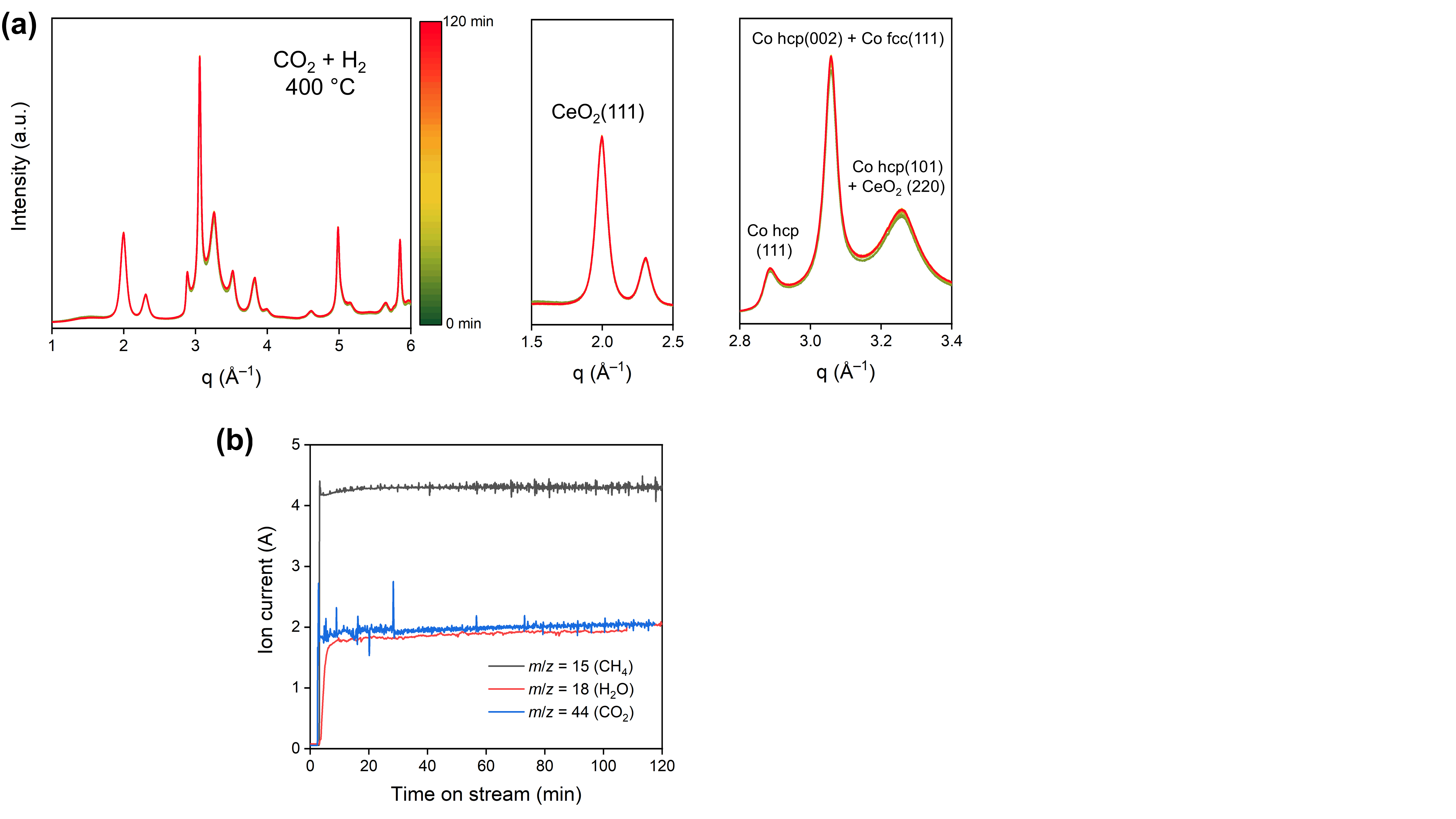


**Figure S26**. (a) Operando XRD patterns during CO_2_ hydrogenation at 400 °C for 10Ce90Co and (b) products analysis from MS. The sample were reduced in H_2_ at 550 °C for 1 h. Reaction conditions: 400 °C, CO_2_/H_2_ =1/4.


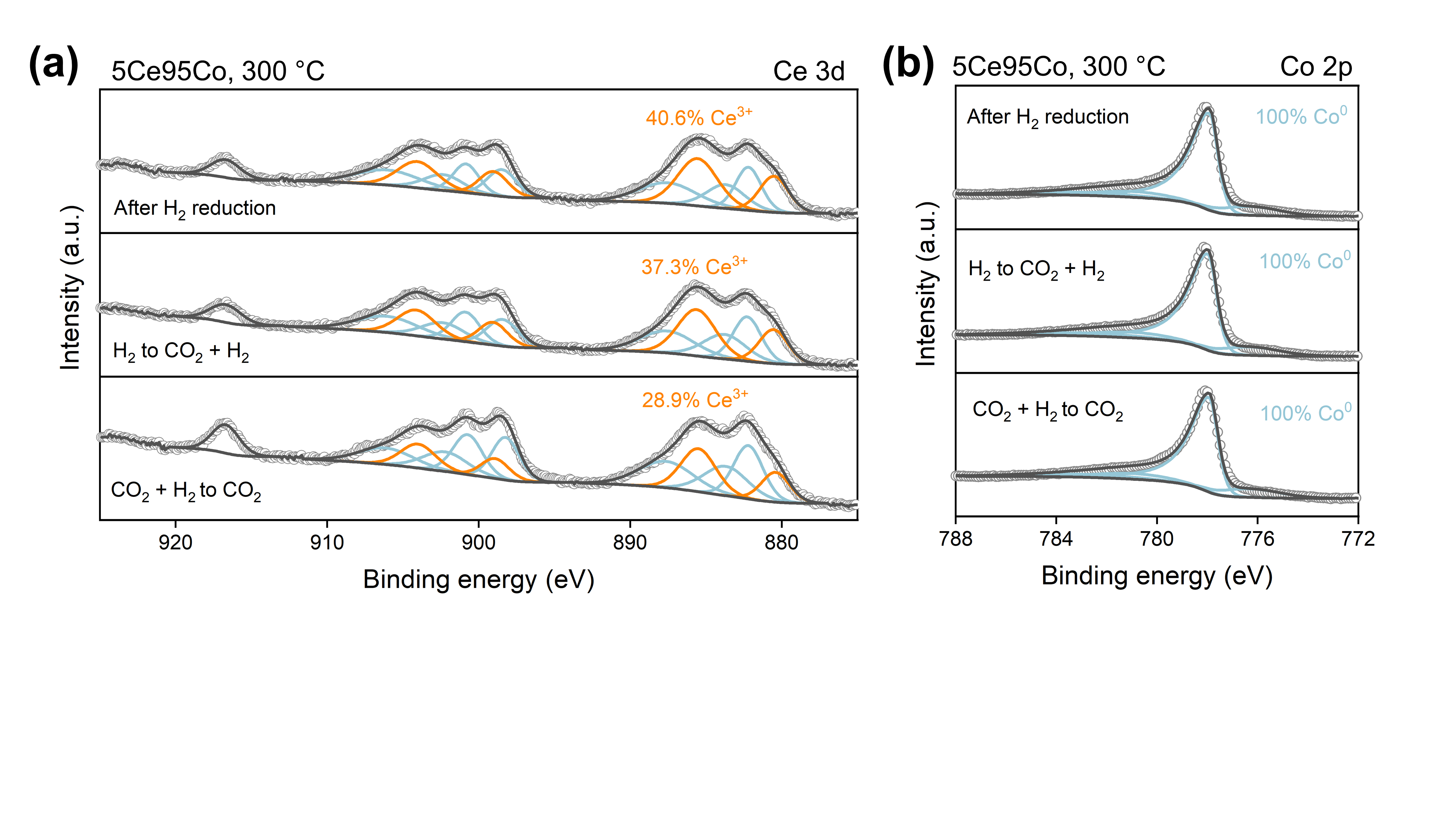


**Figure S27.** (a) Ce 3d core-line and (b) Co 2p core-line NAP-XPS spectra and corresponding fits of 5Ce95Co. The XPS fitting models are shown in **Figure S3**.


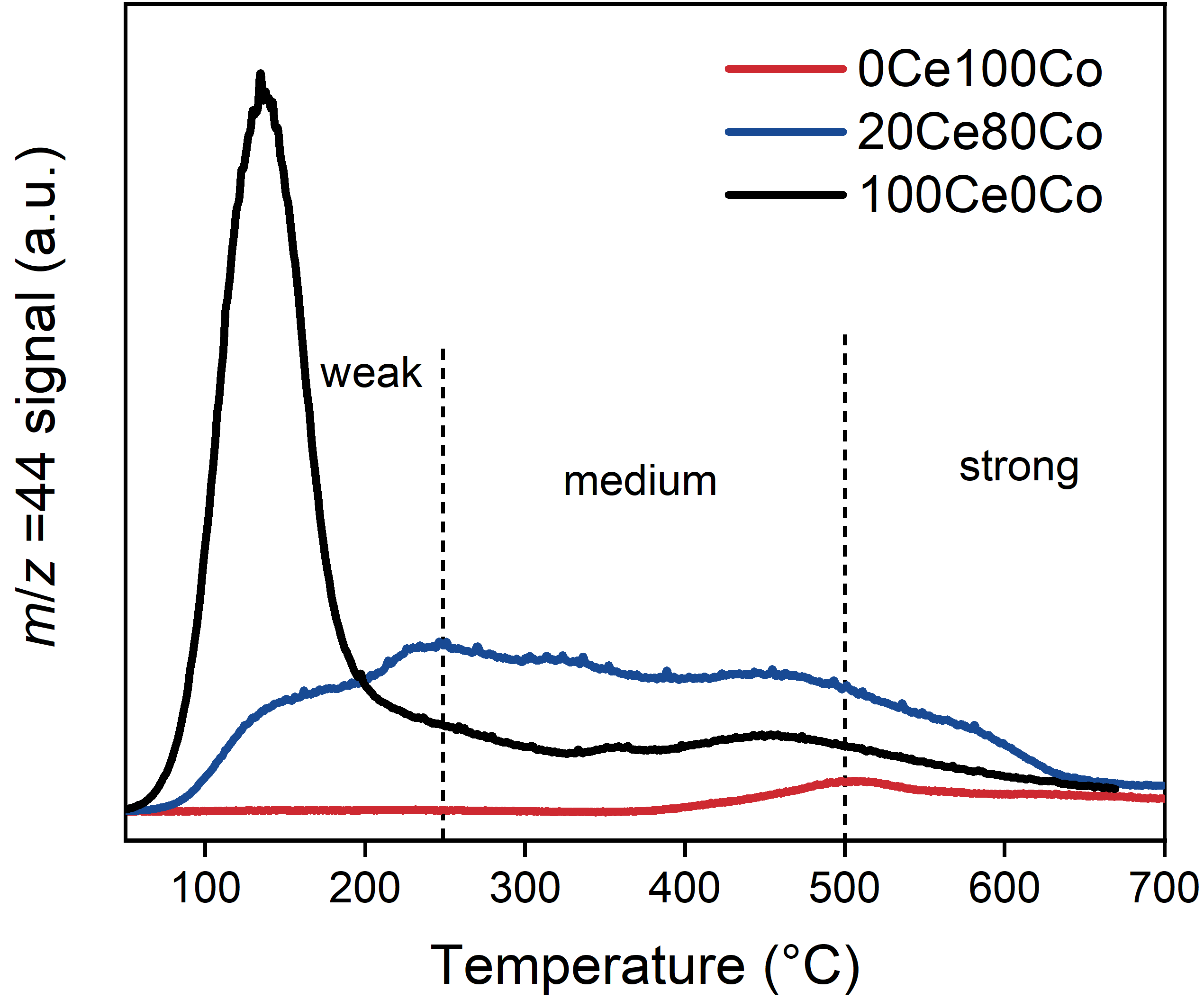


**Figure S28.** CO_2_-TPD profiles of catalysts after reduction in 10 vol% H_2_ in He (50 mL/min) at 300 °C for 4 h.


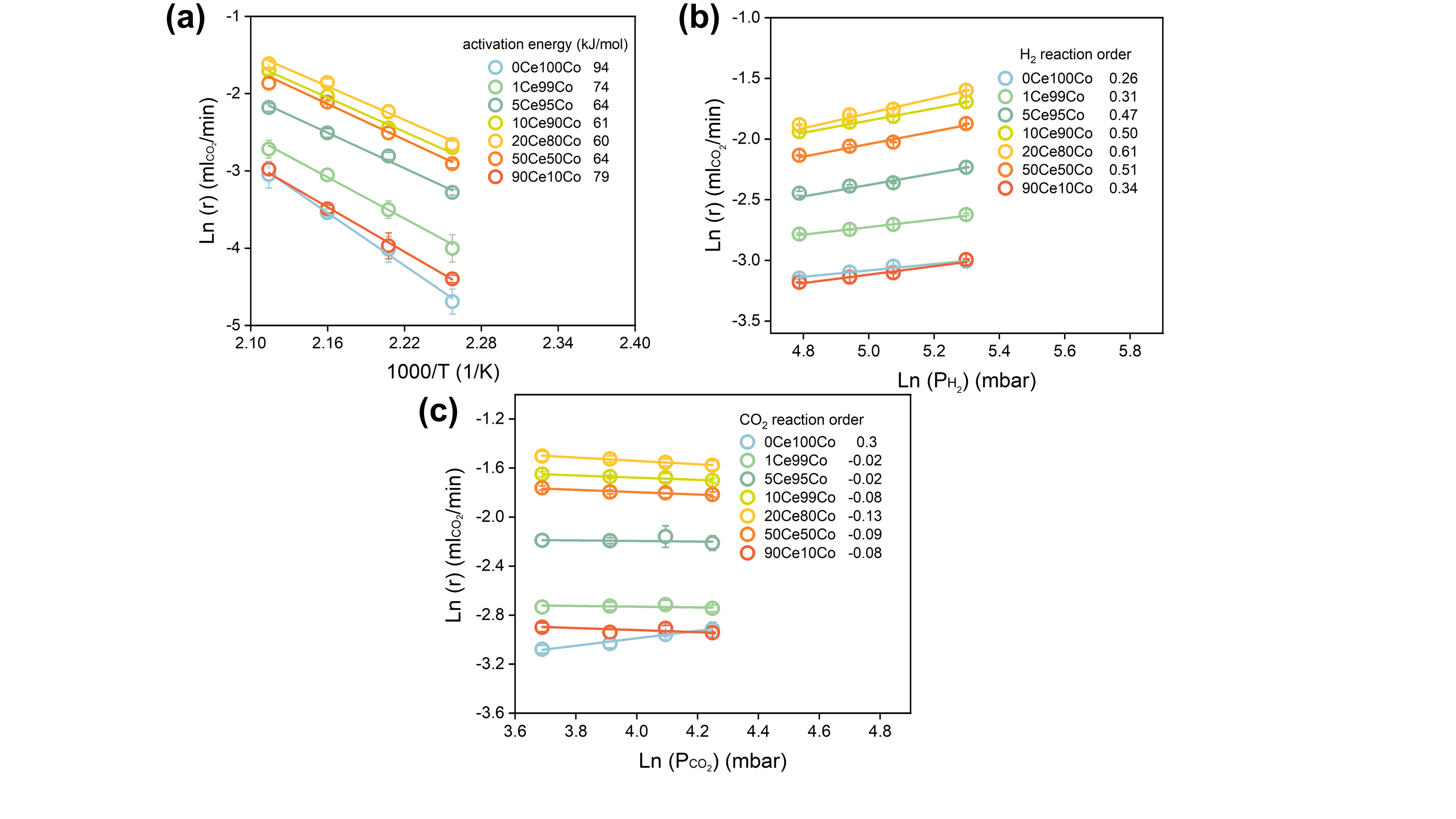


**Figure S29.** Kinetic parameters of CO_2_ hydrogenation: (a) apparent activation energy; (b) H_2_ reaction order and (c) CO_2_ reaction order after reduction in 10 vol% H_2_ in He (50 mL/min) at 300 °C for 4 h.


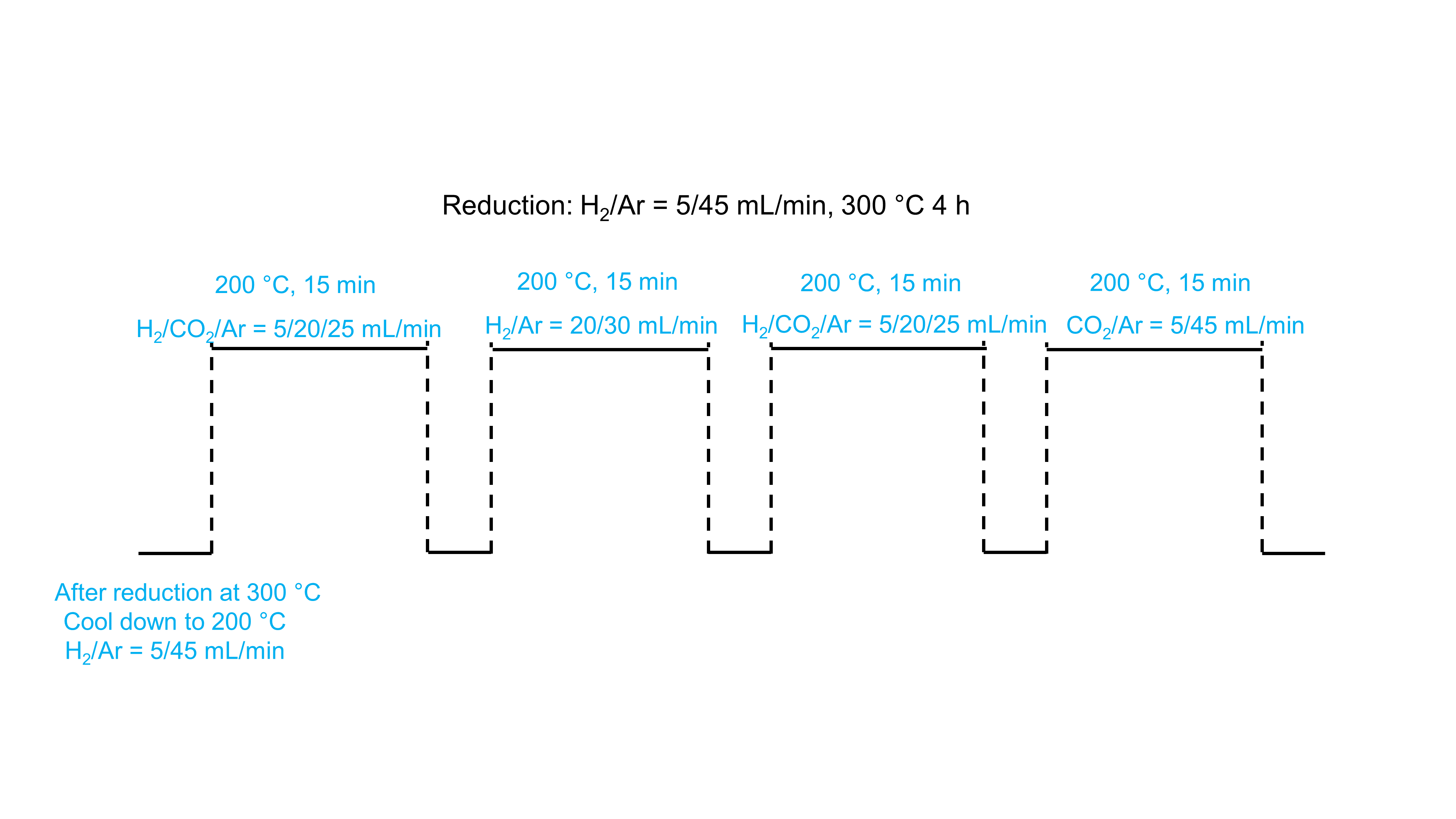


***Scheme S1.*** *Schematic representation of the procedure used in the transient operando DRIFTS-MS experiments.*


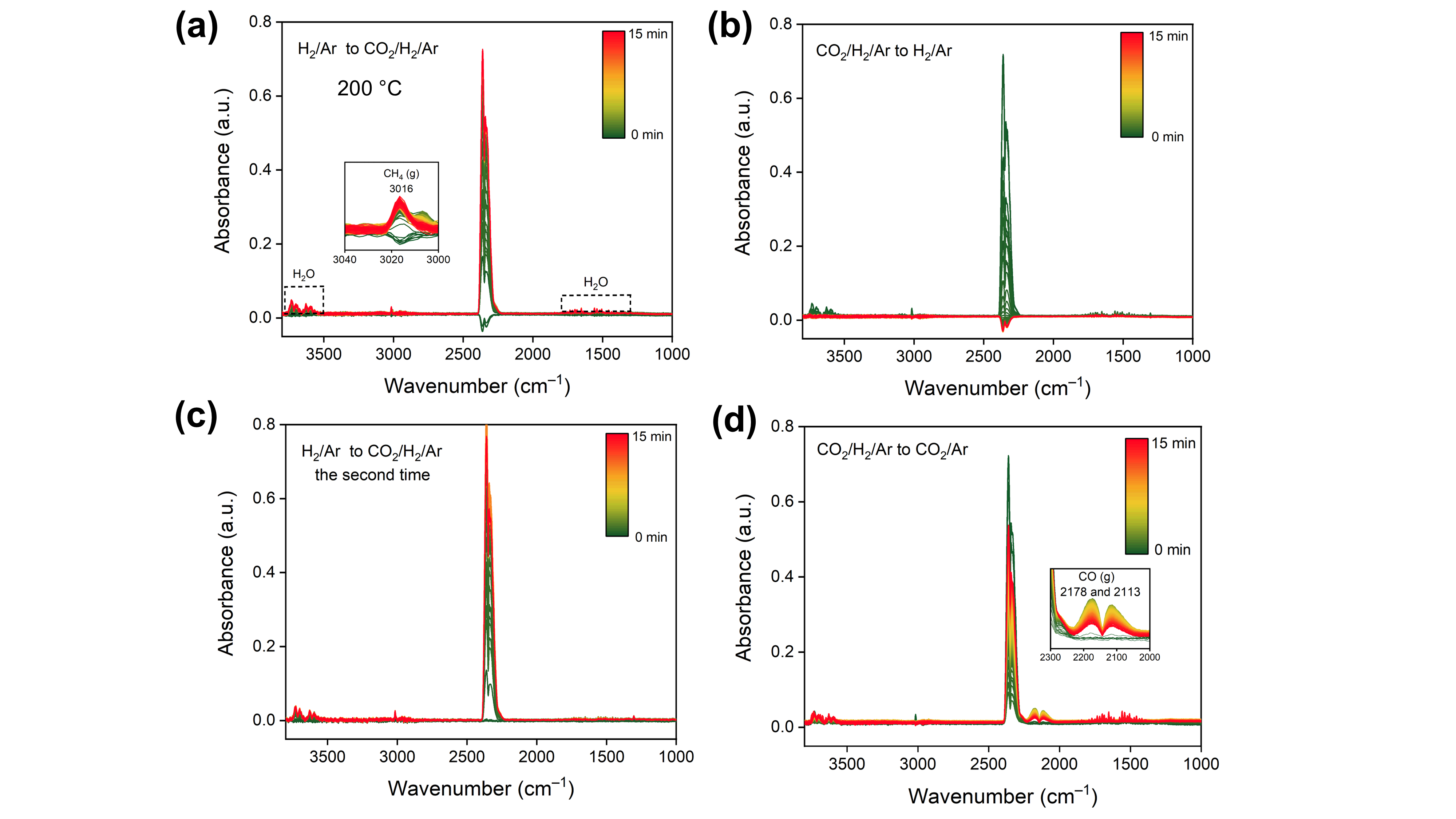


**Figure S30.** Transient operando DRIFTS-MS results of 0Ce100Co after switching from (a) H_2_/Ar (pretreatment) to CO_2_/H_2_/Ar; (b) CO_2_/H_2_/Ar to H_2_/Ar; (c) H_2_/Ar to CO_2_/H_2_/Ar and (d) CO_2_/H_2_/Ar to CO_2_/Ar. Reaction temperature: 200 °C.


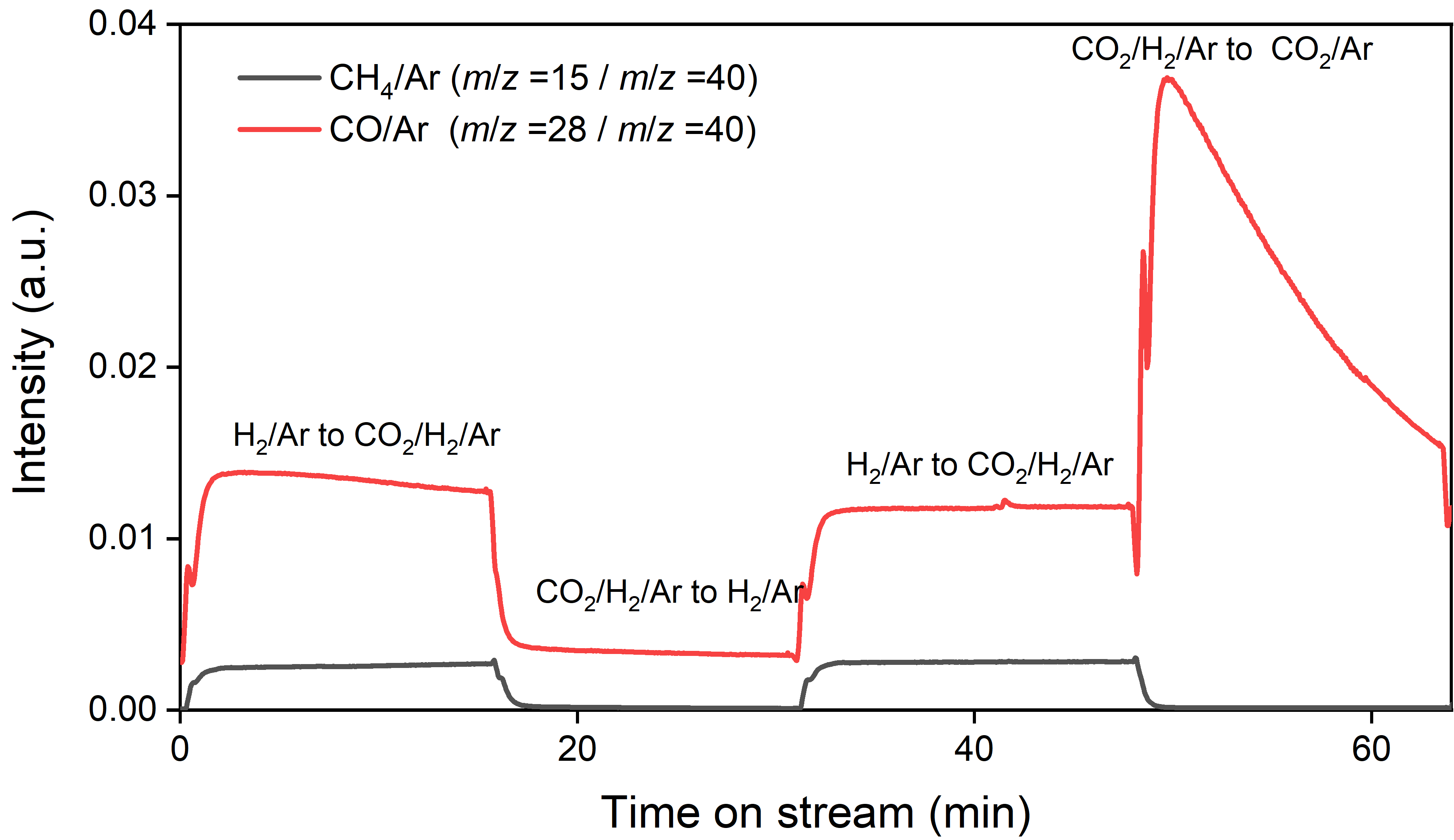


**Figure S31.** Normalized CH_4_ and CO signal in MS for 0Ce100Co during transient operando DRIFTS-MS experiments. Reaction temperature: 200 °C.


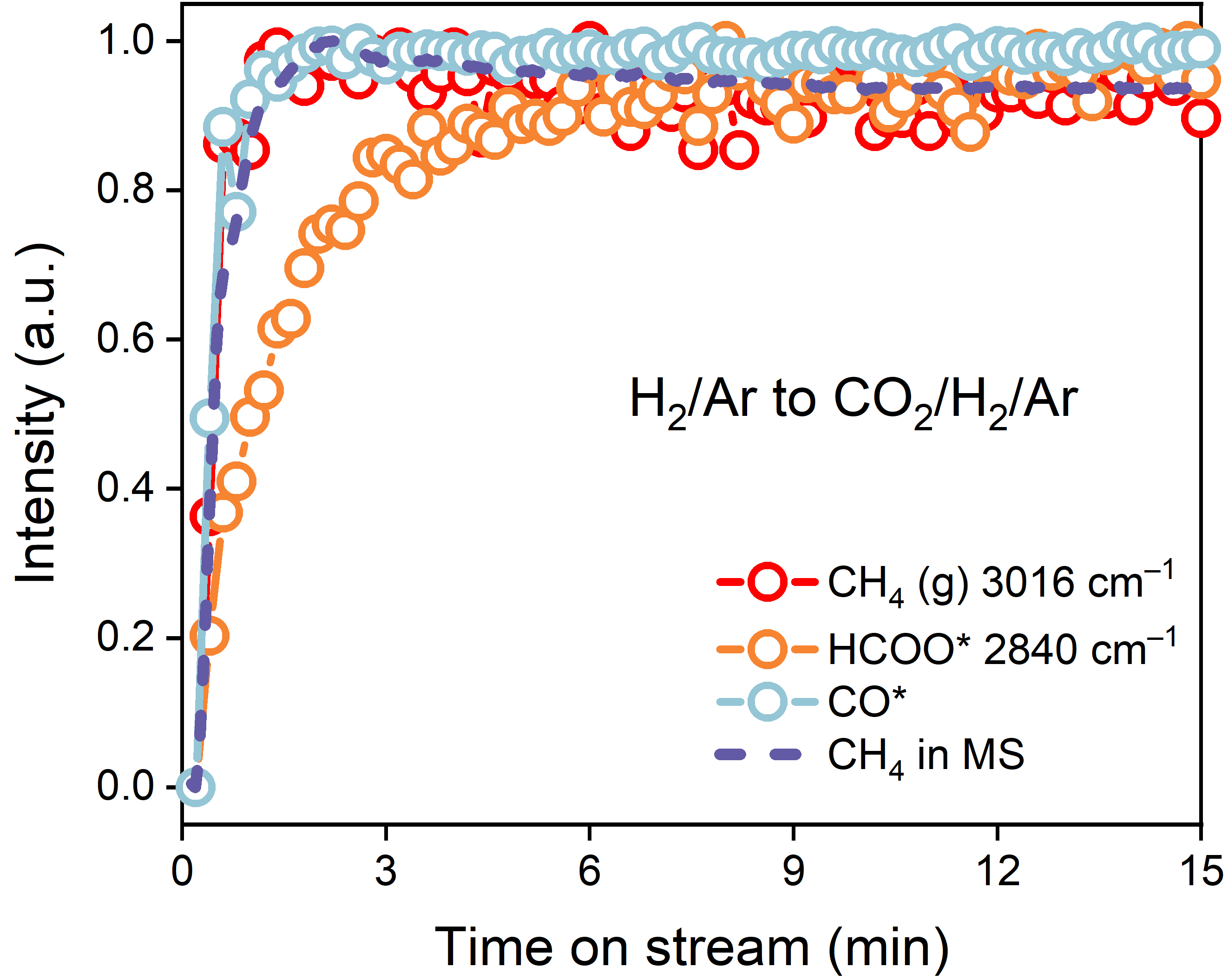


**Figure S32.** Normalized CO*, HCOO*, CH_4_ signal in IR spectra and CH_4_ signal in MS for 20Ce80Co during transient operando DRIFTS-MS experiments. Reaction temperature: 200 °C.


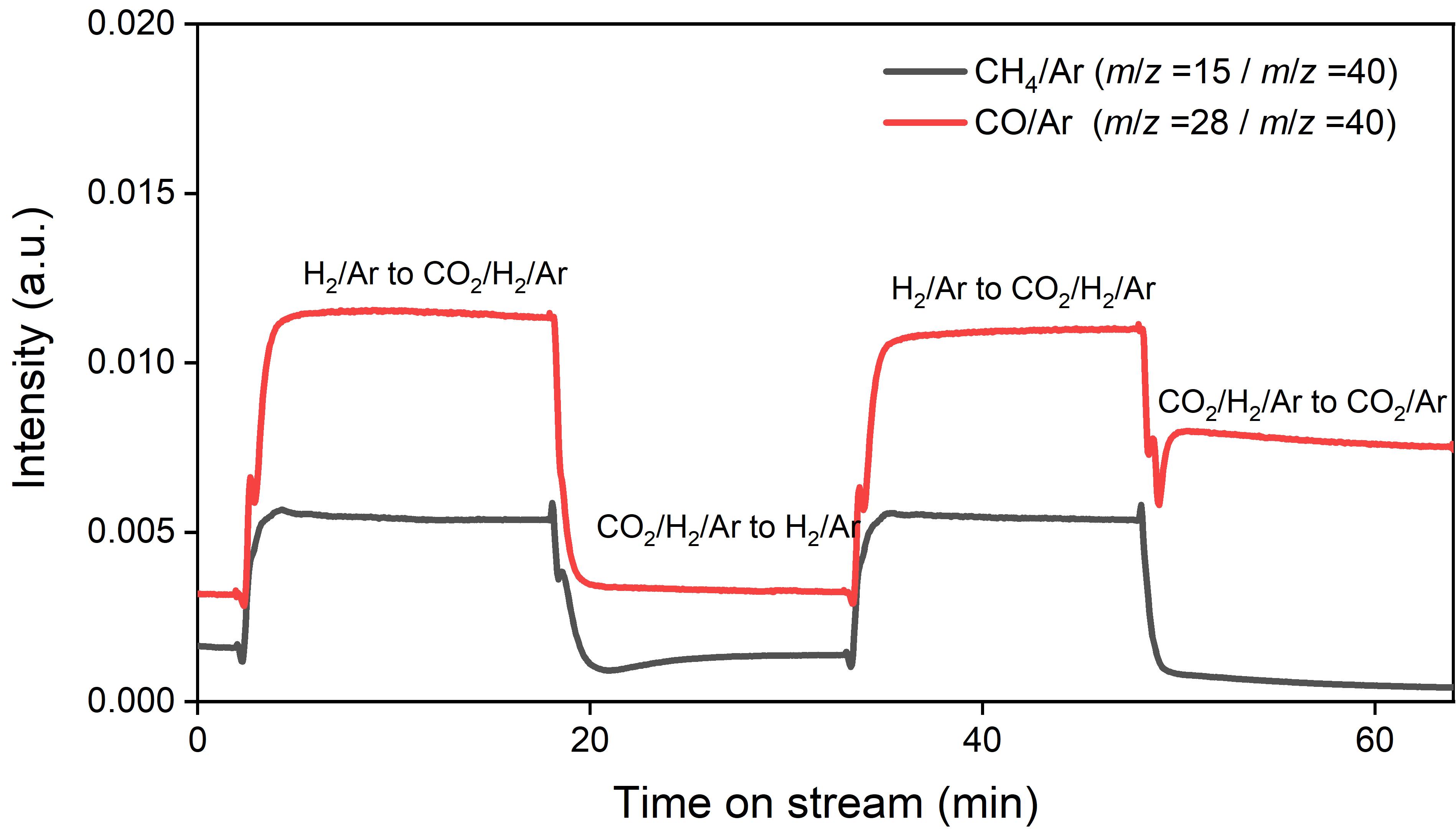


**Figure S33.** Normalized CH_4_ and CO signal in MS for 20Ce80Co during the transient operando DRIFTS-MS experiments. Reaction temperature: 200 °C.


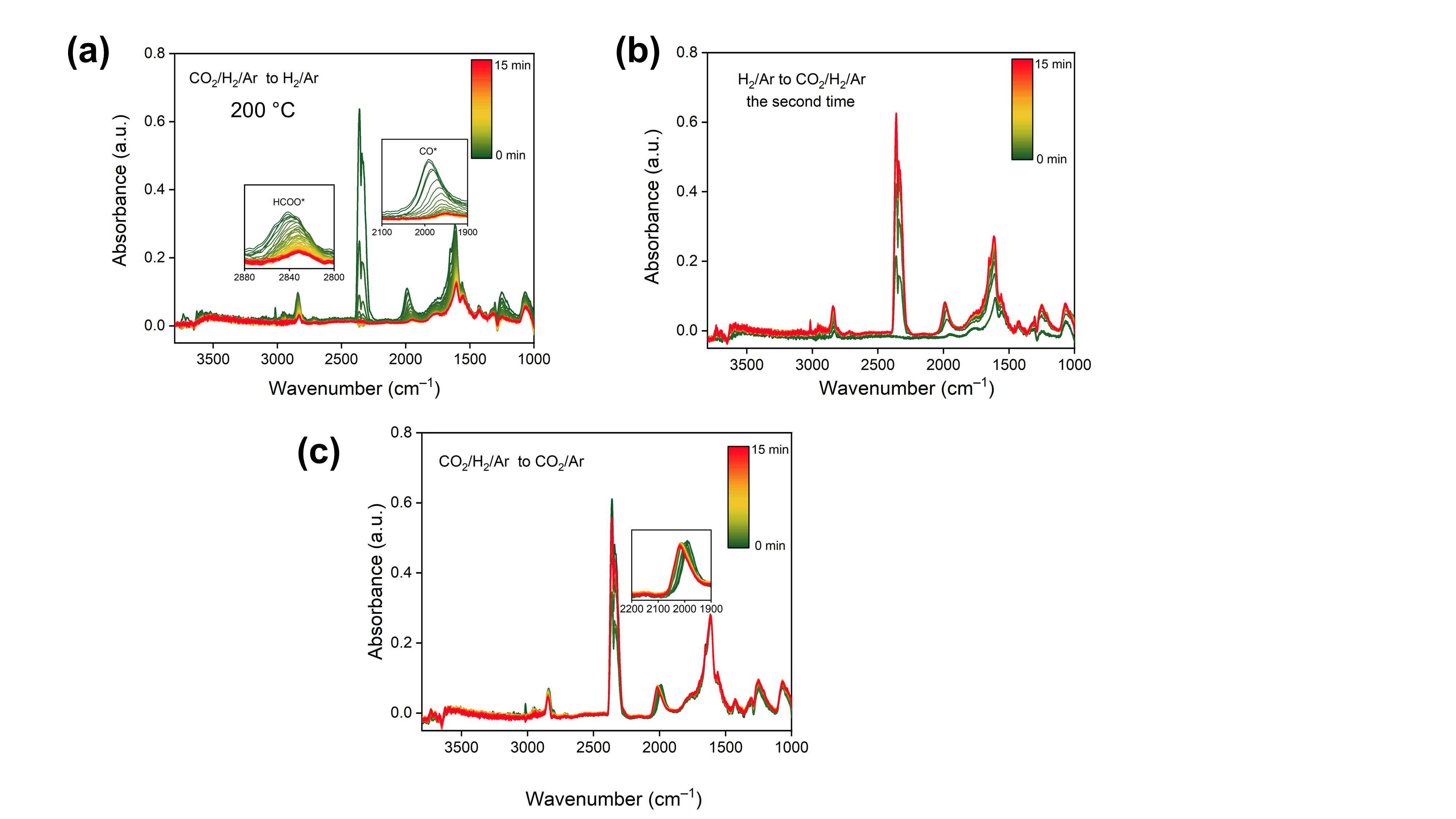


**Figure S34.** Transient operando DRIFTS-MS results of 20Ce80Co after switching from (a) CO_2_/H_2_/Ar to H_2_/Ar; (b) H_2_/Ar to CO_2_/H_2_/Ar and (c) CO_2_/H_2_/Ar to CO_2_/Ar (results of the first switch from H_2_/Ar (pretreatment) to CO_2_/H_2_/Ar shown in **Figure 6e**). Reaction temperature: 200 °C.


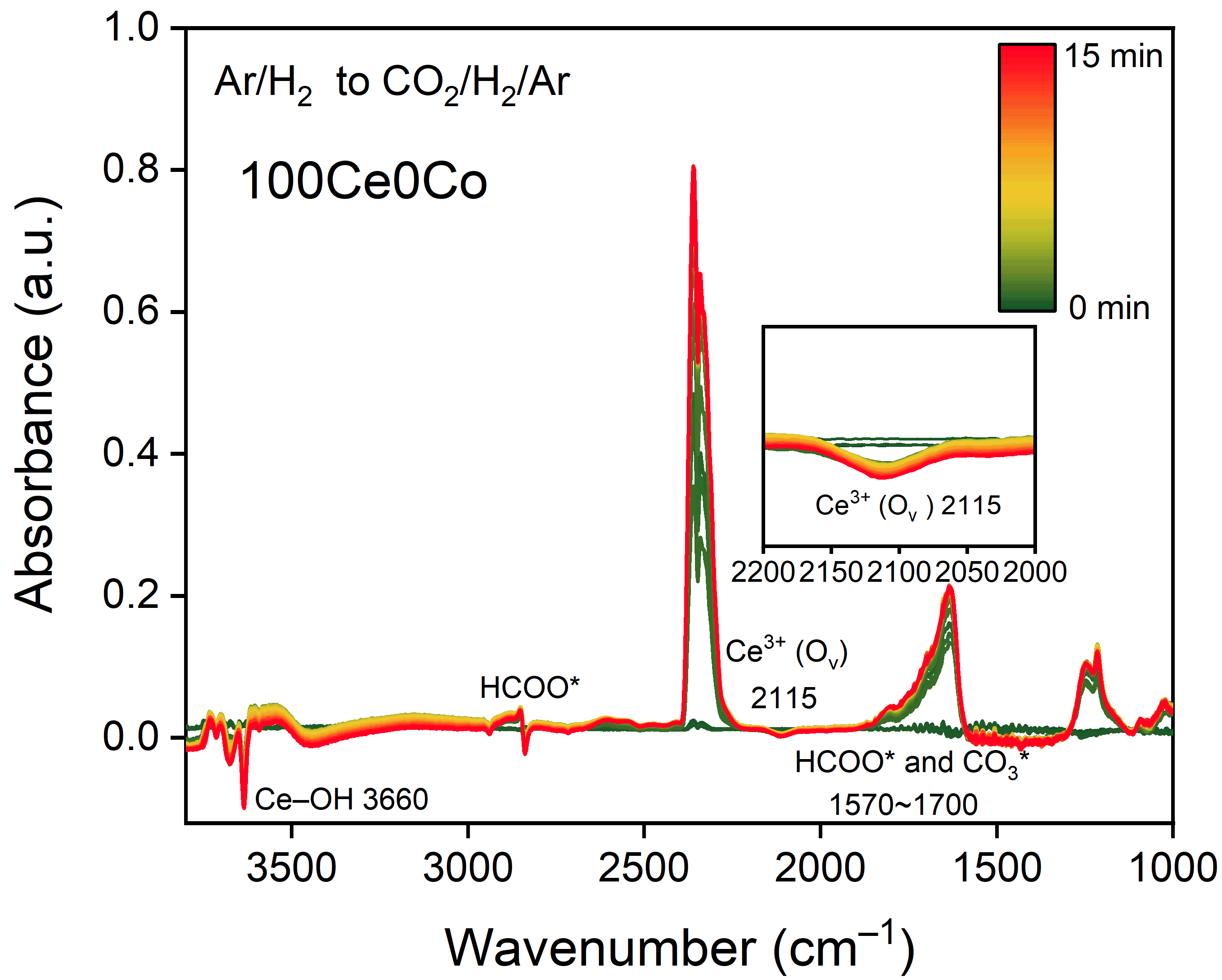


**Figure S35.** Transient operando DRIFTS-MS results of 100Ce0Co after switching from H_2_/Ar (pretreatment) to CO_2_/H_2_/Ar. Reaction temperature: 200 °C.


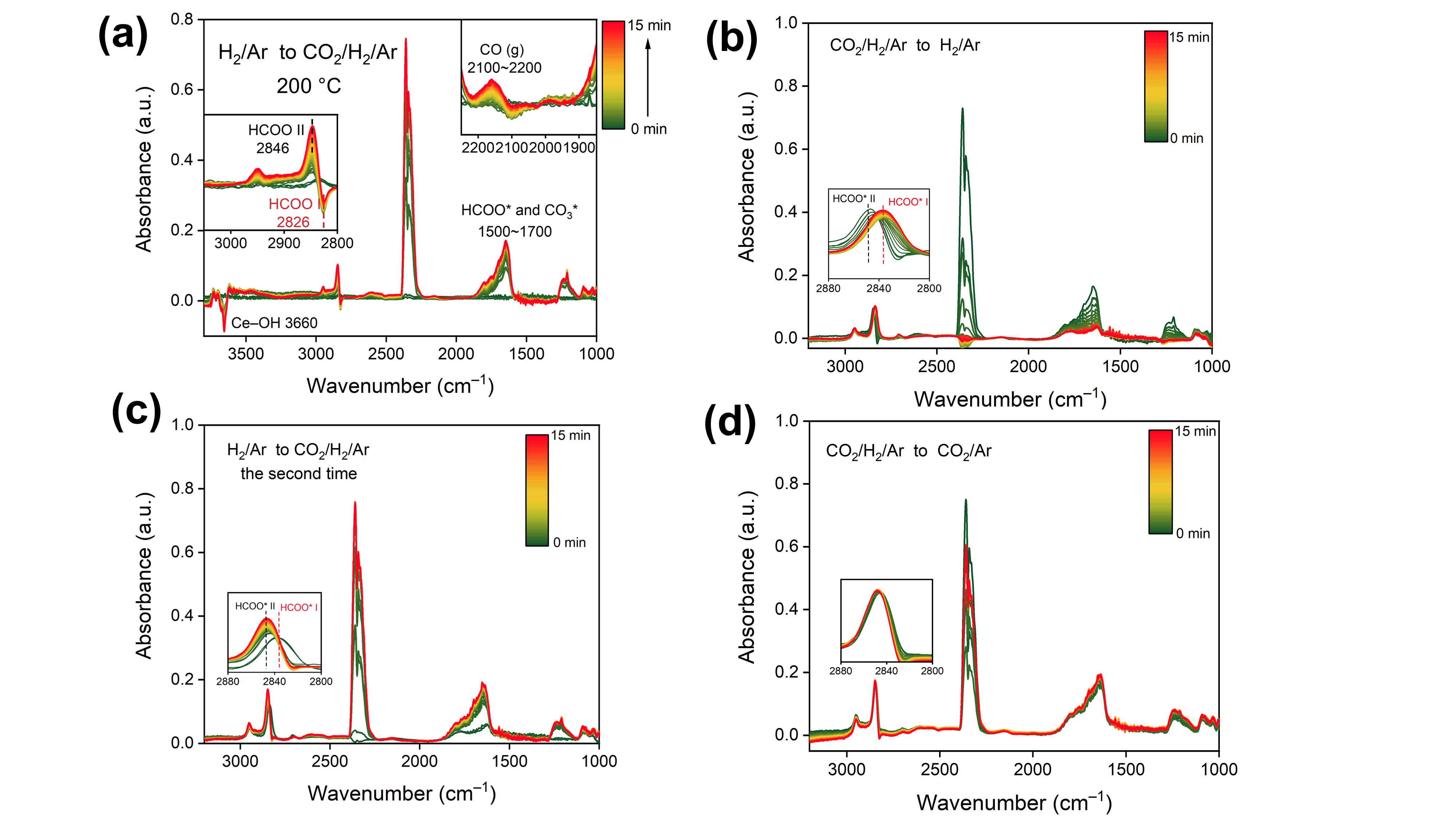


**Figure S36.** Transient operando DRIFTS-MS results of 90Ce10Co after switching from (a) H_2_/Ar (pretreatment) to CO_2_/H_2_/Ar; (b) CO_2_/H_2_/Ar to H_2_/Ar; (c) H_2_/Ar to CO_2_/H_2_/Ar and (d) CO_2_/H_2_/Ar to CO_2_/Ar. Reaction temperature: 200 °C.


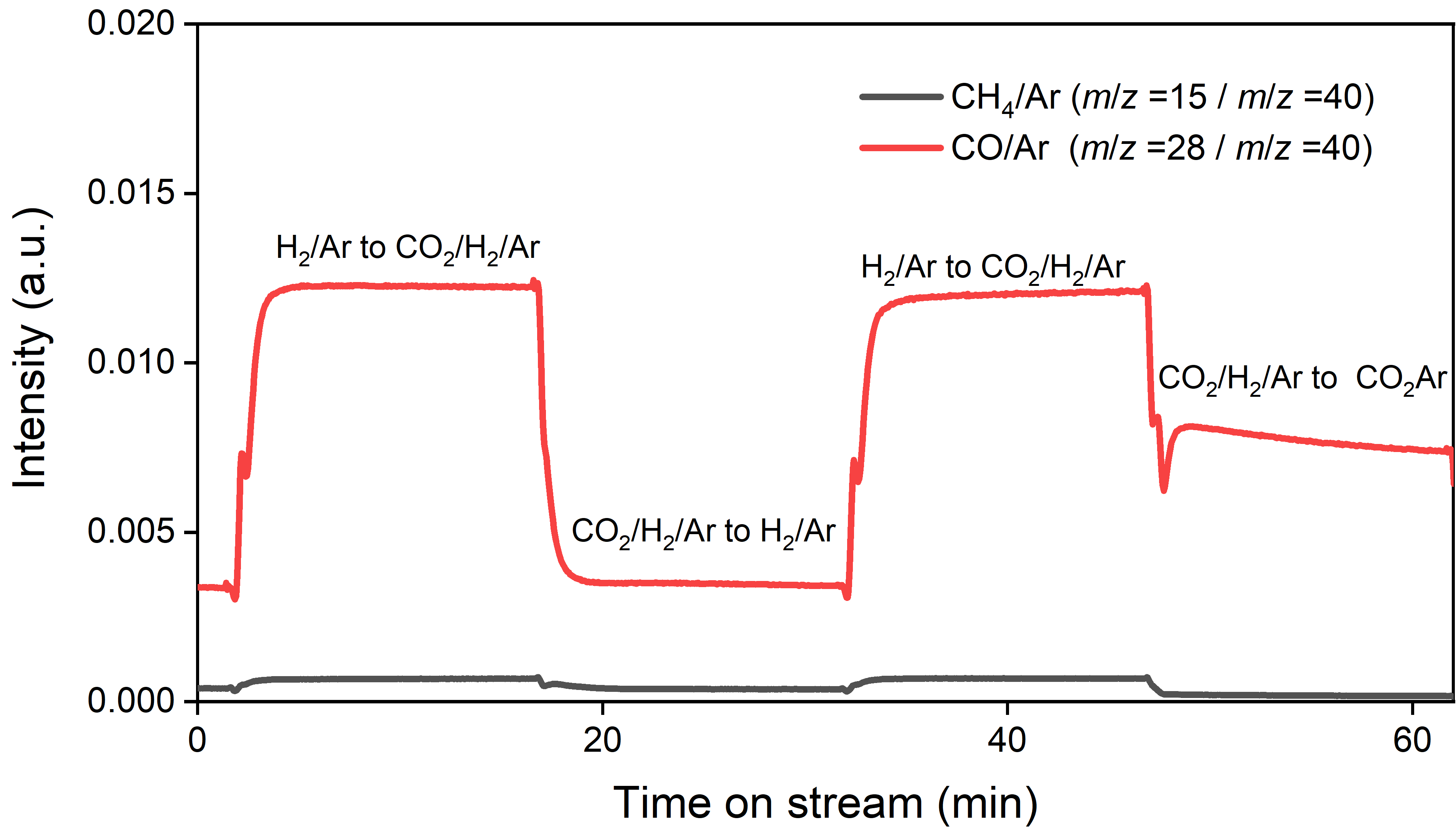
**Figure S37.** Normalized CH_4_ and CO signal in MS for 90Ce10Co during the transient operando DRIFTS-MS experiments. Reaction temperature: 200 °C.


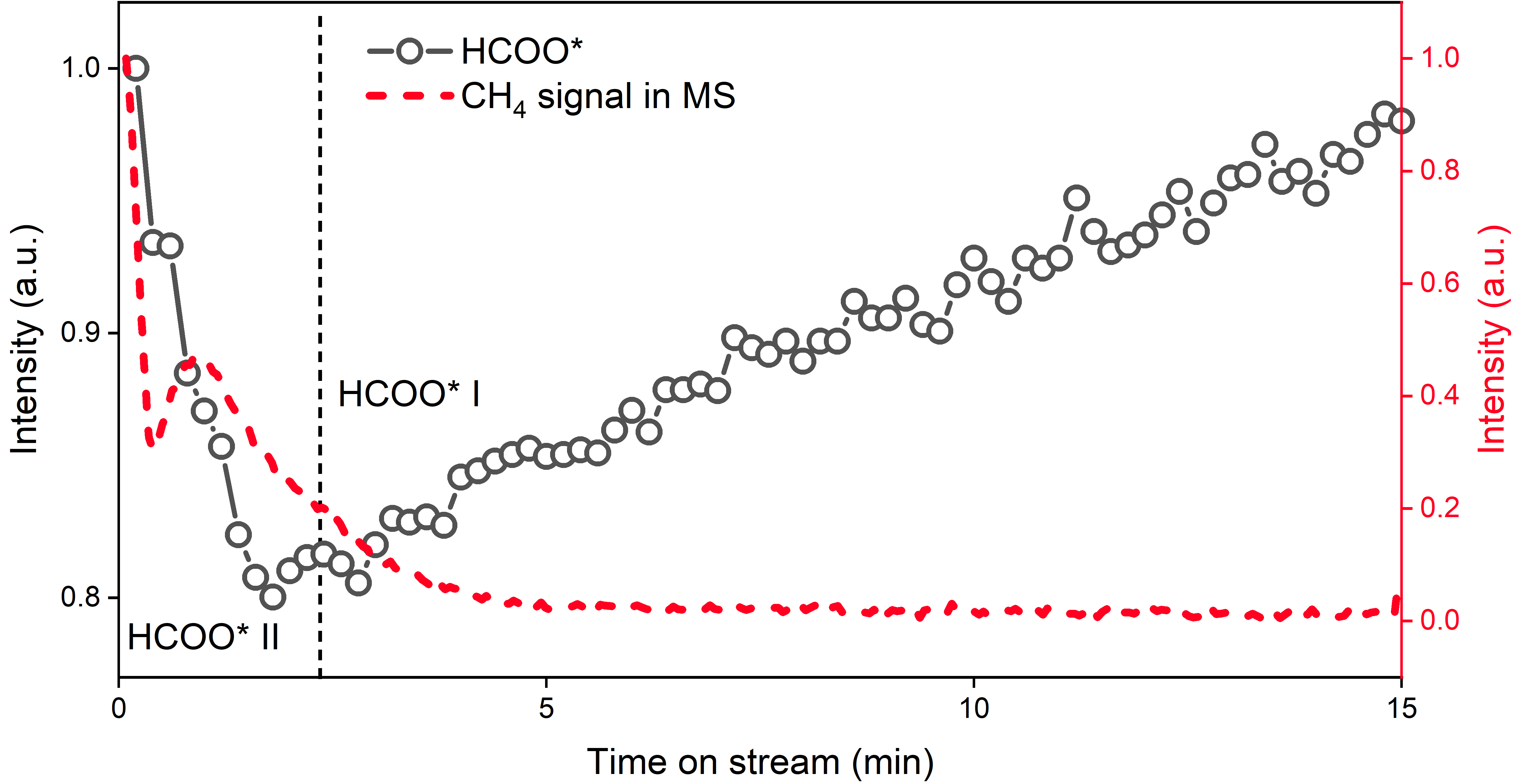


**Figure S38.** Correlation between CH_4_ signal and HCOO* intensity after switching from CO_2_/H_2_/Ar to H_2_/Ar in 10Ce90Co. Reaction temperature: 200 °C.

***Note S2.***

Typically, on reducible supports, (bi-)CO_3_* and HCOO* species can be formed through the reaction of CO_2_ with surface hydroxyl groups and oxygen vacancies, respectively.^[35]^ This is evident from the strongly negative band of Ce−OH (~3660 cm^−1^) and the weakly negative band of the 2F_5/2_ to 2F_7/2_ electronic transition of Ce^3+^ (~2115 cm^−1^) in the CeO_2_ refernence sample 100Ce0Co (**Figure S35**).^[36,37]^ The higher HCOO* intensity in 90Ce10Co as compared to that in 100Ce0Co indicates Co-Ce interface sites promote the formation of HCOO* (**Figure S36**). Furthermore, two types of HCOO* (HCOO* I at 2826 cm^−1^ and HCOO* II at 2845 cm^−1^) can be observed in the spectra. CO* formation was minimal. By normalizing the intensity of HCOO* and CH_4_ signal in MS upon switching from CO_2_/H_2_/Ar to H_2_/Ar, we observed that CH_4_ intensity correlated with the intensity of the HCOO* II band and not with that of the HCOO* I band (**Figure S37** and **S38**). Therefore, the HCOO* II band is most likely involved in the reaction mechanism, involving an associative pathway.^[38,39]^

References

[1] T. Skála, F. Šutara, K. C. Prince, V. Matolín, *J. Electron Spectros. Relat. Phenomena* **2009**, *169*, 20.

[2] S. Kato, M. Ammann, T. Huthwelker, C. Paun, M. Lampimäki, M. T. Lee, M. Rothensteiner, J. A. Van Bokhoven, *Phys. Chem. Chem. Phys.* **2015**, *17*, 5078.

[3] M. C. Biesinger, B. P. Payne, A. P. Grosvenor, L. W. M. Lau, A. R. Gerson, R. S. C. Smart, *Appl. Surf. Sci.* **2011**, *257*, 2717.

[4] A. Parastaev, V. Muravev, E. Huertas Osta, A. J. F. van Hoof, T. F. Kimpel, N. Kosinov, E. J. M. Hensen, *Nat. Catal.* **2020**, *3*, 526.

[5] F. Hu, C. Jin, R. Wu, C. Li, G. Song, T. Z. H. Gani, K. H. Lim, W. Guo, T. Wang, S. Ding, R. Ye, Z. H. Lu, G. Feng, R. Zhang, S. Kawi, *Chem. Eng. J.* **2023**, *461*, 142108.

[6] Y. T. Li, L. Zhou, G. N. Han, W. G. Cui, W. Li, T. L. Hu, *Int. J. Hydrogen Energy* **2024**, *51*, 452.

[7] Y. Wang, H. Arandiyan, J. Scott, H. Dai, R. Amal, *Adv. Sustain. Syst.* **2018**, *2*, 1700119.

[8] A. Efremova, I. Szenti, J. Kiss, Á. Szamosvölgyi, A. Sápi, K. Baán, L. Olivi, G. Varga, Z. Fogarassy, B. Pécz, Á. Kukovecz, Z. Kónya, *Appl. Surf. Sci.* **2022**, *571*, 151326.

[9] Z. Ai, W. Na, J. Li, Z. Huang, H. Huang, Y. Peng, W. Gao, H. Wang, *Catal Lett.* **2024**, *154*, 5110.

[10] G. Varga, I. Szenti, J. Kiss, K. Baán, G. Halasi, L. Óvári, A. Szamosvölgyi, R. Mucsi, E. Dodony, Z. Fogarassy, B. Pécz, L. Olivi, A. Sápi, A. Kukovecz, Z. Kónya, *J. CO2 Util.* **2023**, *75*, 102582.

[11] G. Varga, A. Sápi, T. Varga, K. Baán, I. Szenti, G. Halasi, R. Mucsi, L. Óvári, J. Kiss, Z. Fogarassy, B. Pécz, Á. Kukovecz, Z. Kónya, *J. Catal.* **2020**, *386*, 70.

[12] Y. Zhu, S. Zhang, Y. Ye, X. Zhang, L. Wang, W. Zhu, F. Cheng, F. Tao, *ACS Catal.* **2012**, *2*, 2403.

[13] L. Deng, X. Liu, R. Wang, C. Wang, G. Zhou, *J. Alloys Compd.* **2022**, *918*, 165472.

[14] Y. Zhou, Y. Jiang, Z. Qin, Q. Xie, H. Ji, *Chin. J. Chem. Eng.* **2018**, *26*, 768.

[15] H. H. Shin, L. Lu, Z. Yang, C. J. Kiely, S. McIntosh, *ACS Catal.* **2016**, *6*, 2811.

[16] N. Gholampour, Y. Zhao, F. Devred, C. Sassoye, S. Casale, D. P. Debecker, *ChemCatChem* **2023**, *15*, e202201338.

[17] N. Albeladi, Q. A. Alsulami, K. Narasimharao, *Top. Catal.* **2025**, 68, 229.

[18] D. Jampaiah, D. Damma, A. Chalkidis, P. Venkataswamy, S. K. Bhargava, B. M. Reddy, *Catal. Today* **2020**, *356*, 519.

[19] O. V. Ischenko, A. G. Dyachenko, I. Saldan, V. V. Lisnyak, V. E. Diyuk, A. V. Vakaliuk, A. V. Yatsymyrskyi, S. V. Gaidai, T. M. Zakharova, O. Makota, T. Ericsson, L. Häggström, *Int. J. Hydrogen Energy* **2021**, *46*, 37860.

[20] Z. Qin, X. Wang, L. Dong, T. Su, B. Li, Y. Zhou, Y. Jiang, X. Luo, H. Ji, *Chem. Eng. Sci.* **2019**, *210*, 115245.

[21] G. Zhou, T. Wu, H. Xie, X. Zheng, *Int. J. Hydrogen Energy* **2013**, *38*, 10012.

[22] K. Jiang, Y. Men, S. Liu, J. Wang, W. An, H. Yu, E. W. Shin, *J. CO2 Util.* **2021**, *53*, 101743.

[23] L. Xu, X. Lian, M. Chen, Y. Cui, F. Wang, W. Li, B. Huang, *Int. J. Hydrogen Energy* **2018**, *43*, 17172.

[24] Z. Li, J. Chen, Y. Xie, J. Wen, H. Weng, M. Wang, J. Zhang, J. Cao, G. Tian, Q. Zhang, P. Ning, *J. Energy Chem.* **2024**, *91*, 213.

[25] M. Schubert, S. Pokhrel, A. Thomé, V. Zielasek, T. M. Gesing, F. Roessner, L. Mädler, M. Bäumer, *Catal. Sci. Technol.* **2016**, *6*, 7449.

[26] T. Zhao, Y. Hui, Niamatullah, Z. Li, *Mol. Catal.* **2019**, *474*, 110421.

[27] X. Zou, Z. Shen, X. Li, Y. Cao, Q. Xia, S. Zhang, Y. Liu, L. Jiang, L. Li, L. Cui, Y. Wang, *J. Colloid Interface Sci.* **2022**, *620*, 77.

[28] Z. Gou, S. Chen, D. Zheng, X. Wang, H. Xie, G. Zhou, *ChemistrySelect* **2023**, *8*, e202203627.

[29] G. Zhou, H. Liu, Y. Xing, S. Xu, H. Xie, K. Xiong, *J. CO2 Util.* **2018**, *26*, 221.

[30] H. Liu, S. Xu, G. Zhou, K. Xiong, Z. Jiao, S. Wang, *Fuel* **2018**, *217*, 570.

[31] L. Xu, Y. Cui, M. Chen, X. Wen, C. Lv, X. Wu, C. E. Wu, Z. Miao, X. Hu, *Ind. Eng. Chem. Res.* **2021**, *60*, 8056.

[32] Y. Wu, J. Lin, Y. Xu, G. Ma, J. Wang, M. Ding, *ChemCatChem* **2020**, *12*, 3553.

[33] L. Deng, Y. Tan, C. Chen, F. Li, X. Zhang, *New J. Chem.* **2023**, *47*, 8032.

[34] J. Díez-Ramírez, P. Sánchez, V. Kyriakou, S. Zafeiratos, G. E. Marnellos, M. Konsolakis, F. Dorado, *J. CO2 Util.* **2017**, *21*, 562.

[35] J. C. Lavalley, *Catal. Today* **1996**, *27*, 377.

[36] X. Zou, Z. Shen, X. Li, Y. Cao, Q. Xia, S. Zhang, Y. Liu, L. Jiang, L. Li, L. Cui, Y. Wang, *J. Colloid Interface Sci.* **2022**, *620*, 77.

[37] S. Lin, Z. Li, M. Li, *Fuel* **2023**, *333*, 126369.

[38] J. J. C. Struijs, V. Muravev, M. A. Verheijen, E. J. M. Hensen, N. Kosinov, *Angew. Chem. Int. Ed.* **2023**, *135*, e202214864.

[39] R. P. Ye, Q. Li, W. Gong, T. Wang, J. J. Razink, L. Lin, Y. Y. Qin, Z. Zhou, H. Adidharma, J. Tang, A. G. Russell, M. Fan, Y. G. Yao, *Appl. Catal. B* **2020**, *268*, 118474.
